# Total Fat and Fatty Acid Composition of Preterm Human Milk: A Systematic Review and Meta-analysis

**DOI:** 10.1016/j.advnut.2026.100661

**Published:** 2026-05-26

**Authors:** Mindy A Patterson, Derek C Miketinas, Vanessa K Thyne, Dixie L Carter, Maria Fernanda Nunez, Katie E Niemeier, Jennifer N Kinnaman, Tonya M Bender, Ariana DL Bailey

**Affiliations:** 1 Department of Nutrition and Food Sciences, Texas Woman’s University, Houston, TX, United States; 2 Institute for Women’s Health, Texas Woman’s University, Houston, TX, United States; 3 Global Research & Development, Mead Johnson Nutrition, Evansville, IN, United States

**Keywords:** fat, fatty acids, preterm, human milk, milk, colostrum, transition, mature

## Abstract

Preterm human milk (HM) is abundant in fat and fatty acids (FAs) that contribute to overall energy intake and are vital for growth, cell integrity, and brain development. The objective of this systematic review and meta-analyses is to quantify the amount of total fat and FAs including long-chain FAs in preterm HM. Ebsco, PubMed, and Scopus databases were searched to July 2023 measuring total fat and FAs in preterm HM. Three reviewers (DCM, MAP, & ADLB), working independently, screened all titles and abstracts to identify studies meeting inclusion criteria [preterm <37 wk; Human Development Index >0.8; cross-sectional, case-controlled (*n* > 1), prospective cohort, and randomized clinical trials; English language]. Random effects models were used to estimate mean total fat and FA content across studies. Data were aggregated for studies reporting repeated measures. Heterogeneity was estimated using *I*^*2*^ and publication bias using Kendall tau rank correlation coefficient. Of the 884 articles identified, a total of 69 original studies were included for the meta-analysis comprising an estimated 1871 preterm infants (gestational age range: 23.2–36.6 wk). Mean (95% confidence interval) total fat was 3.76 (3.57, 3.95) g/100 mL, which varied across colostrum [<5 d; 3.04 (2.51, 3.57) g/100 mL], transition [5–14 d; 3.74 (3.47, 4.00) g/100 mL], and mature [>14 d, 3.85 (3.61, 4.08) g/100 mL] preterm HM. The creamatocrit method provided the highest total fat composition followed by infrared, other/not-specified, then gravimetric. Total mean FA content from 23 studies (836 preterm infants) also varied, where the most abundant was oleic acid (18:1*n*–9; 35.7% w/w FA) and palmitic acid (16:0; 22.05% w/w FA). The total mean essential FA concentrations were 13.7% w/w for 18:2 *n*–6 and 1.0% w/w for 18:3*n*–3. This systematic review provides updated estimates of total fat and FA concentrations in preterm HM, although high variability among study design and quality, analytical methodologies, and data reporting were found across studies.

This trial was registered at PROSPERO as CRD42023445191.

**Statement of Significance**

The current manuscript advances the field of preterm human milk (HM) nutrition by providing up-to-date estimates of total fat and fatty acids in colostrum, transition, and mature preterm HM. These values fulfill a critical need for practitioners and researchers to more accurately understand the nutritional needs of preterm infants, as well as any supplementary nutrition that may be necessary for appropriate growth and development.

## Introduction

Lipids are abundant in preterm human milk (HM), accounting for ∼40% to 55% of the digestible energy required for rapid growth and development [1]. Lipids are the most energy-dense macronutrient, providing a greater energy supply in a smaller volume to the preterm infant thereby helping to reduce the risk of high osmolar load and hyperglycemia [2]. In addition to fat-soluble vitamins and complex lipids, HM lipids provide numerous bioactive compounds that play significant roles in metabolism, immune and gastrointestinal function, and neurodevelopment [1].

Total HM fat is dominated by triacylglycerols (98%–99%), which primarily comprise fatty acids (FA) that determine the nutritional and physicochemical properties of HM lipids. It has been estimated that ∼40% to 45% of HM FAs are saturated, 40% to 45% are monounsaturated, and 15% to 20% are polyunsaturated [1,3]. Certain FAs like DHA and arachidonic acid (ARA) are critical for cellular maintenance and integrity, neuronal growth and brain development, and retinal and visual development. However, these FAs are accreted most rapidly during the third trimester, and infants born preterm, especially those born extremely preterm (< 28 wk of gestation) and/or at extremely low body weight (<1000 g), are unable to amass these FAs sufficiently. Additionally, the conversions of precursor FAs, α-linolenic acid (ALA) and linoleic acid (LA), to DHA and ARA, respectively, are limited and may be further reduced if the precursors are prioritized for energy [4,5]. This consequently puts the preterm infant at greater risk of delayed growth and development if these FAs are not supplemented adequately after birth. To support clinically important outcomes like growth, a clearer understanding of the total fat and FA content of preterm HM will enable clinicians to optimize HM fortification and more closely address gaps in lipid-related nutrient delivery.

The primary objective of this systematic review was to estimate the total fat and FA content and variability of preterm HM using a series of meta-analyses, stratified by milk type and analytical method. Others have characterized the total fat and FA content of preterm HM; however, the studies were either published almost a decade or longer ago [[6], [7], [8], [9]] or combined preterm HM with term HM [10,11]. The present study included studies that reported fat and/or FA concentrations of preterm HM exclusively. The secondary objective was to stratify total fat by analytical method. The broader research aim was to conduct a systematic review and a series of meta-analyses to estimate preterm HM nutrient composition and characteristics.

## Methods

This is the second among a series of meta-analyses that utilized 1 systematic review process to identify inclusion criteria to estimate the nutrient composition of preterm HM. These meta-analyses examine total fat and FA composition of HM and share a similar methodology to the first publication in this series [12]. The protocol was registered at PROSPERO (CRD42023445191; https://www.crd.york.ac.uk/PROSPERO/display_record.php?RecordID=445191). The PRISMA checklist of items can be found in Supplementary Table 1.

### Search strategy and eligibility criteria

The search strategy and eligibility criteria were recently published [12]. In summary, the eligibility criteria consisted of studies published in English until 2023 and that reported preterm [defined as gestational age (GA) <37 wk or stated as preterm by authors] HM nutrient composition. Developing countries [defined as a Human Development Index (HDI) <0.8] at the time of publication were excluded, as well as those studies with an *n* = 1.

### Data extraction

Covidence software was used to identify articles meeting inclusion criteria. After duplicates were removed by the software, 2 independent reviewers (DCM and MAP) systematically reviewed the searched titles, abstracts, and full-text articles. Any disagreements were resolved with a third researcher (ADLB).

Three researchers (DCM, VKT, and MAP) extracted the following data from the included studies: name of first author, publication year, country, study design, sample size of infants, GA, age at which HM was collected, milk type (colostrum, transition, and mature if specified by the article), HM collection method, and total fat and/or FA analysis method. If milk type was not explicitly stated, postpartum day of HM collection was used to classify milk type. Any discrepancies in data extraction were reviewed by reviewers and corrected. Reported units were converted to g/100 mL for fat and %w/w for FAs where applicable.

### Statistical analysis

These analyses were restricted to fat and FA composition. Preterm HM samples were treated as the sampling unit unless not provided; otherwise, number of infants was used. For studies that reported total fat and/or FA across subgroups, the total sample mean and SD were estimated. Supplementary Table 2 provides the data handling steps for how missing data were treated. For randomized controlled trials, baseline or control group milk composition estimates were extracted. Units were converted to g/100 mL for total fat and %w/w for FAs when necessary assuming HM density (1.03 g/mL) [13], and FA concentration of fat (88% of total fat) [14]. For studies reporting %creamatocrit, the equation published by Lucas was used [15]. Supplementary Table 3 provides example conversions for these analyses.

These meta-analyses were conducted in R using the metafor package [16] to fit random effects models to estimate total fat and FA content across HM type [colostrum (<5 d), transition (5–14 d), and mature (>14 d)]. The total fat content of mature HM was further stratified by 15 to 42 d, 43 to 84 d, and >84 d [5]. For studies that reported total fat content, subgroup analyses were conducted across analytical methods: infrared, creamatocrit, gravimetric, and other/not-specified. Estimates from studies reporting multiple observations per milk type were aggregated to a single study-level value using inverse-variance weighting. To account for dependence among within-study observations, the variance of the aggregated estimate was inflated using a prespecified correlation parameter. Heterogeneity was tested using Cochrane-*I*^*2*^ statistics. The level of heterogeneity (*I*^*2*^) was measured as a percentage where <40% is low, 40%–75% is moderate, and ≥75% is high heterogeneity. Publication bias was assessed using Kendall’s Tau rank correlation coefficient. Sensitivity analysis was conducted to evaluate the impact of each study on the pooled estimates.

## Results

### Study selection

**FIGURE 1:**
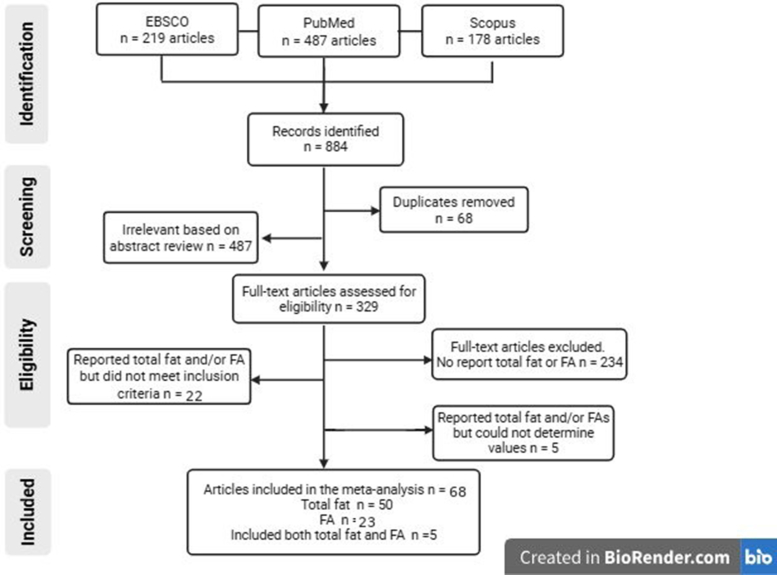
Study selection flowchart. FA, fatty acid.

Of the 884 studies identified, 68 were removed as duplicates, 487 were considered irrelevant based on title and abstract review, leaving 329 studies that involved full-text review (Figure 1). Of those, 234 were excluded due to not reporting total fat and/or FAs. An additional 26 studies reporting total fat and/or FAs were further excluded (21 did not meet the inclusion criteria; 5 provided results in a figure without values). Thus, 68 articles were included in the analysis (50 total fat only, 23 FAs only, and 5 reported both).

### Total fat

Of the 50 articles that reported total fat (Table 1 [[17], [18], [19], [20], [21], [22], [23], [24], [25], [26], [27], [28], [29], [30], [31], [32], [33], [34], [35], [36], [37], [38], [39], [40], [41], [42], [43], [44], [45], [46], [47], [48], [49], [50], [51], [52], [53], [54], [55], [56], [57], [58], [59], [60], [61], [62], [63], [64], [65], [66]]), 15 reported fat content in colostrum milk (<5 d), 32 reported fat content in transition milk (5–14 d) and 39 reported fat content in mature milk (>14 d). Of the studies reporting milk type (e.g., C, T, or M), 37 studies reported >1, 10 studies reported only 1, and 3 studies did not specify. Thus, the number of studies that contributed to each milk type exceeded the number of articles. The HM storage method also differed across studies: room temperature (*n* = 7), refrigerated (*n* = 7), and frozen (*n* = 22). Many studies (*n* = 14) did not specify the storage method of HM before analysis. The HM collection protocol also varied: 24-h pooled (*n* = 25), fore- and hind-milk (*n* = 2), complete emptying of the breast (*n* = 1), and unspecified (*n* = 22). The infant GA range was 23.2 to 36.6 wk, and the range in which the HM was collected was 0 to 368 d.

**TABLE 1:** Summary of the characteristics of studies reporting total fat (*n* = 50)

Author, first	Year	Country	Study design	Infants, *n*	GA, wk^1^	HM age, d^2^	HM type^3^	HM storage method	HM collection protocol	Fat analysis method^4^
Abdulrazzaq, Y [17]	2003	UAE	CS	49	(<37.0)	(0, 21)	C, T, M	Unknown/not-specified	Breast completely emptied	IS
Aceti, A [18]	2009	Italy	CS	17	(24.0, 33.0)	27 (12, 145)	M	Unknown/not-specified	24-h pooled	IS
Anderson, D [19]	1983	United States	L	14	31.0 (28.0, 36.0)	(3, 14)	C, T, M	Frozen	24-h pooled	O
Anderssen, S [20]	2015	Norway	L	47	(26.0, 36.0)	(6, 39)	T, M	Unknown/not-specified	24-h pooled	IS
Bauer, J [21]	2011	Germany	L	102	(23.0, 33.0)	31.5 (7, 56)	T, M	Frozen	24-h pooled	C
Belfort, M [22]	2020	United States	L	37	28.2 (23.6, 31.9)	58.5 (5, 112)	M	Refrigerated	24-h pooled	IS
Brion, L [23]	2020	United States	RCT	58	28.0	—		Unknown/not-specified	Unknown/not-specified	IS
Bulut, Ö [24]	2019	Turkey	L	32	(25.0, 33.0)	(2, 42)	C, T, M	Refrigerated	24-h Pooled	IS
Butte, N [25]	1984	United States	L	8	33.9 (30.0, 36.0)	(14, 84)	T, M	Frozen	Unknown/not-specified	G
Campbell-Yeo, M [26]	2010	Canada	RCT	24	26.8	(0, 14)	C, T	Frozen	24-h pooled	G
Corvaglia, L [27]	2008	Italy	CS	55	(26.0, 32.0)	10	C, T	Refrigerated	Unknown/not-specified	O
Darwish, A [28]	1989	Egypt	L	35	(28.0, 36.0)	(3, 18)	C, T, M	Frozen	Unknown/not-specified	O
de Halleux, V [29]	2013	Belgium	L	28	28.6	28	M	Refrigerated	Unknown/not-specified	IS
de Oliveira, S [30]	2017	France	RCT	12	30.0 (28.1, 31.7)	27	T M	Refrigerated	Unknown/not-specified	O
Ehrenkranz, R [31]	1984	United States	L	21	29.0 (26.0, 33.0)	(2, 42)	C, T, M	Frozen	24-h pooled	G
Erickson, T [32]	2013	United States	L	8	—	(5, 32)	T, M	Frozen	24-h pooled	C
Faerk, J [33]	2001	Denmark	L	101	28.0	(7, 70)	T, M	Unknown/not-specified	24-h pooled	
Gao, C [34]	2020	Australia	L	32	(34.0, 37.0)	(1, 21)	C, T, M	Room temperature	24-h pooled	O
Gates, A [35]	2021	United States	L	38	28.2 (22.9, 33.0)	(7, 28)	T, M	Frozen	24-h pooled	G
Groh-Wargo, S [36]	2016	United States	CS	10	23.9	28 (7, 43)	T, M	Frozen	24-h pooled	G
Gross, S [37]	1980	United States	L	33	31.4 (28.0, 36.0)	(3, 28)	C, T, M	Frozen	24-h pooled	G
Guerrini, P [38]	1981	Italy	L	25	33.3 (29.0, 37.0)	(2, 30)	C, T, M	Frozen	24-h pooled	G
Héon, M [39]	2016	Canada	RCT	20	27.7	(7, 42)	T, M	Frozen	24-h pooled	O
Kociszewska-Najman, B [40]	2012	Poland	L	22	(26.0, 36.0)	(1, 15)	C, T, M	Room temperature	Unknown/not-specified	C
Kreissl, A [41]	2016	Austria	L	76	(23.2, 31.3)	(7, 28)	T, M	Unknown/not-specified	Unknown/not-specified	IS
Lemons, J [42]	1982	United States	L	20	33.0 (27.0, 37.0)	(7, 56)	T, M	Frozen	24-h pooled	O
Lev, H [43]	2014	Israel	L	20	30.6 (25.0, 35.0)	(14, 49)	M	Room temperature	Unknown/not-specified	IS
Lin, H [44]	2011	Taiwan	L	14	(27.0, 36.0)	15	M	Frozen	Unknown/not-specified	C
Lubetzky, R [45]	2007	Israel	L	22	28.8 (26.0, 31.0)	(14, 49)	T, M	Unknown/not-specified	Unknown/not-specified	C
Maas, Y [46]	1998	Netherlands	L	79	(25.0, 29.0)	(7, 55)	T, M	Unknown/not-specified	24-h pooled	IS
Maly, J [47]	2019	Czech Republic	L	225	(24.0, 35.0)	(4, 28)	T, M	Frozen	Unknown/not-specified	IS
McLeod, G [48]	2013	Australia	L	63	(24.0, 32.0)	—		Frozen	24-h pooled	O
Meier, P [49]	2002	United States	L	17	28.4 (24.0, 37.0)	(7, 82)	T, M	Room temperature	Fore- and hind-milk	G
Moltó-Puigmartí, C [50]	2011	Spain	CS	20	30.1 (30.0, 37.0)	(2, 32)	C, T, M	Frozen	Unknown/not-specified	C
Moran-Lev, H [51]	2015	Israel	L	32	30.1 (25.0, 35.0)	(7, 49)	M	Frozen	24-h pooled	IS
Morton, J [52]	2012	United States	L	52	(<31.0)	(7, 56)	T, M	Frozen	24-h pooled	O
Norrgrann, M [53]	2023	Sweden	CS	12	28.1 (24.5, 31.0)	(6, 13)	T	Unknown/not-specified	Unknown/not-specified	IS
Paulaviciene, I [54]	2020	Lithuania	CS	27	30.2 (24.0, 36.0)	(14, 16)	M	Refrigerated	Unknown/not-specified	IS
Perrella, S [55]	2015	Australia	RCT	23	(28.0, 32.9)	—		Unknown/not-specified	Unknown/not-specified	C
Phillip, R [56]	2023	Ireland	CS	6	(<28.0)	(7, 28)	T, M	Room temperature	Unknown/not-specified	O
Radmacher, P [57]	2013	United States	CS	83	—	(0, 35)	C, T, M	Room temperature	Unknown/not-specified	IS
Sahin, S [58]	2020	Turkey	L	39	29.7	(3, 28)	C, T, M	Unknown/not-specified	Unknown/not-specified	IS
Sann, L [59]	1981	France	L	41	32.0 (26.0, 35.0)	(6, 15)	T, M	Refrigerated	24-h pooled	O
Sauer, C [60]	2017	United States	L	18	31	(1, 21)	C, T	Unknown/not-specified	Unknown/not-specified	G
Silber, G [61]	1988	United States	L	5	29.6	(0, 4)	C	Unknown/not-specified	24-h pooled	G
Smilowitz, J [62]	2014	United States	L	5	(26.0, 36.0)	(2, 368)	C, T, M	Room temperature	Unknown/not-specified	G
Stoltz Sjöström, E [63]	2014	Sweden	L	256	25.3	(4, 112)	T, M	Unknown/not-specified	24-h pooled	IS
Thomas, M [64]	1986	United States	L	8	(30.0, 34.0)	(14, 18)	M	Frozen	Fore- and hind-milk	O
Whyte, R [65]	1983	Canada	L	9	30 (28.0, 33.0)	(1, 13)	T, M	Frozen	24-h pooled	IS
Zachariassen, G [66]	2013	Denmark	L	214	(<32.0)	(14, 84)	T, M	Frozen	Unknown/not-specified	IS

Abbreviations: C, colostrum; CS, cross-sectional; GA, gestational age; HM, human milk; L, longitudinal; M, mature; RCT, randomized controlled trial; T, transitional.

1 Mean GA. Parentheses indicate upper and lower ranges.

2 Mean age of HM was collected. Parentheses indicate upper and lower ranges.

3 Determined using day HM was collected (C = ≤4 d; T = 5–14 d; M = ≥15 d).

4 Fat analysis method: C, creamatocrit; IS, infrared spectroscopy; G, gravimetric; O, other/not-specified.

**FIGURE 2:**
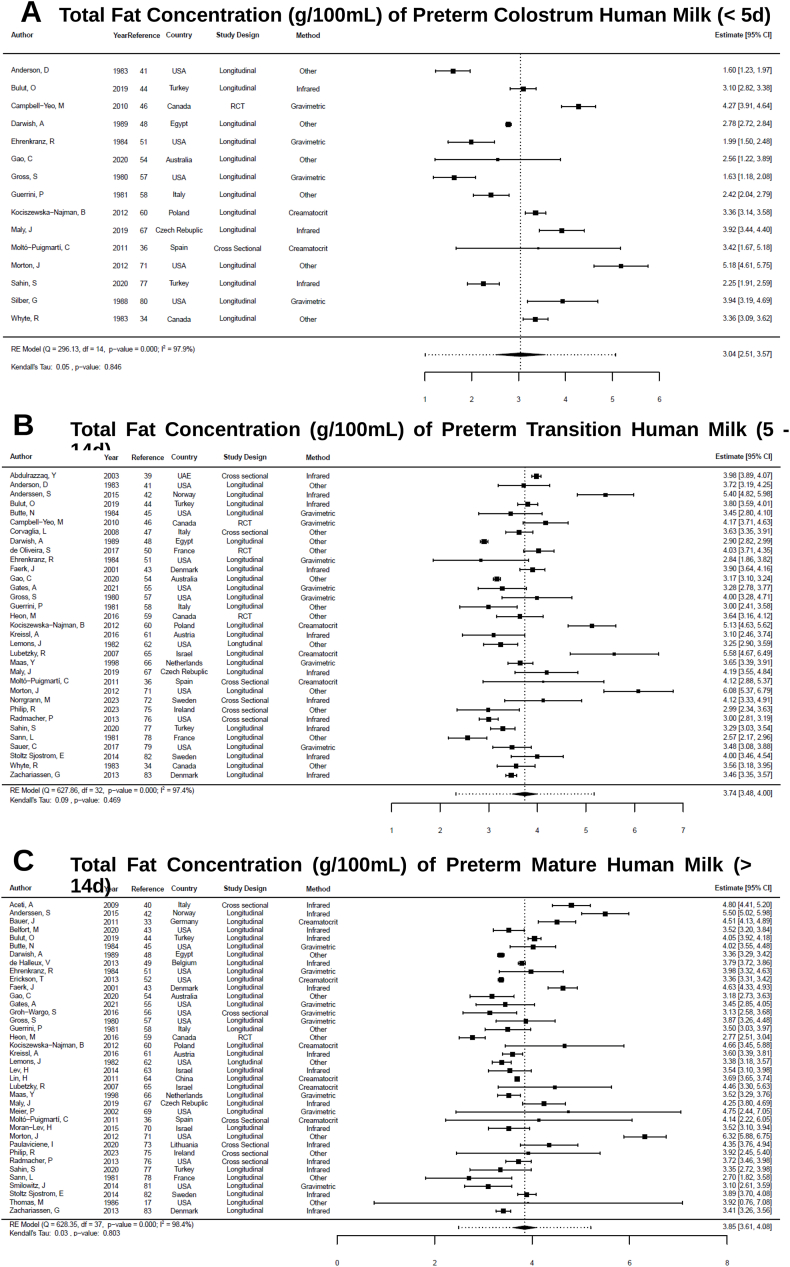
Forest plots for total fat content (g/100 mL) in preterm HM across milk type. (A) Colostrum HM (<5 d), RE model (*Q* = 296.13, df = 14, *P* value < 0.001; *I*^2^ = 97.9%); Kendall’s Tau: 0.05, *P* value = 0.846. (B) Transition HM (5–14 d), RE model (Q = 627.86, df = 32, *P* value < 0.001, *I*^2^ = 97.4%); Kendall’s Tau: 0.9, *P* value = 0.469. (C) Mature HM (>14 d), RE model (*Q* = 628.35, df = 37, *P* value < 0.001, *I*^2^ = 98.4%); Kendall’s Tau: 0.03, *P* value = 0.803. CI, confidence interval; df, degrees of freedom; HM, human milk; RE, regression effects.

Figure 2 represents total fat content (g/100 mL) in preterm HM across milk type (panel A = colostrum; panel B = transition; panel C = mature). The meta-analysis showed the mean for total fat concentration of preterm HM samples as: 3.76 (3.57, 4.95) g/100 mL with a high heterogeneity among studies (*I*^*2*^ = 97.5%). When stratified by milk type, colostrum HM was estimated at 3.04 (2.51, 3.57) g/100 mL, *I*^*2*^ = 97.9%, transition HM overall estimate was 3.74 (3.48, 4.00) g/100 mL, *I*^*2*^ = 97.4%, and mature HM had an overall estimate of 3.85 (3.61, 4.08) g/100 mL, *I*^*2*^ = 98.4%. The high heterogeneity among the studies reflected age at HM collection (range: 1 d–37 wk), feeding phase, storage method (room temperature: 4°C, –70°C), and duration of storage (range: 1–4 d at 4°C).

**FIGURE 3:**
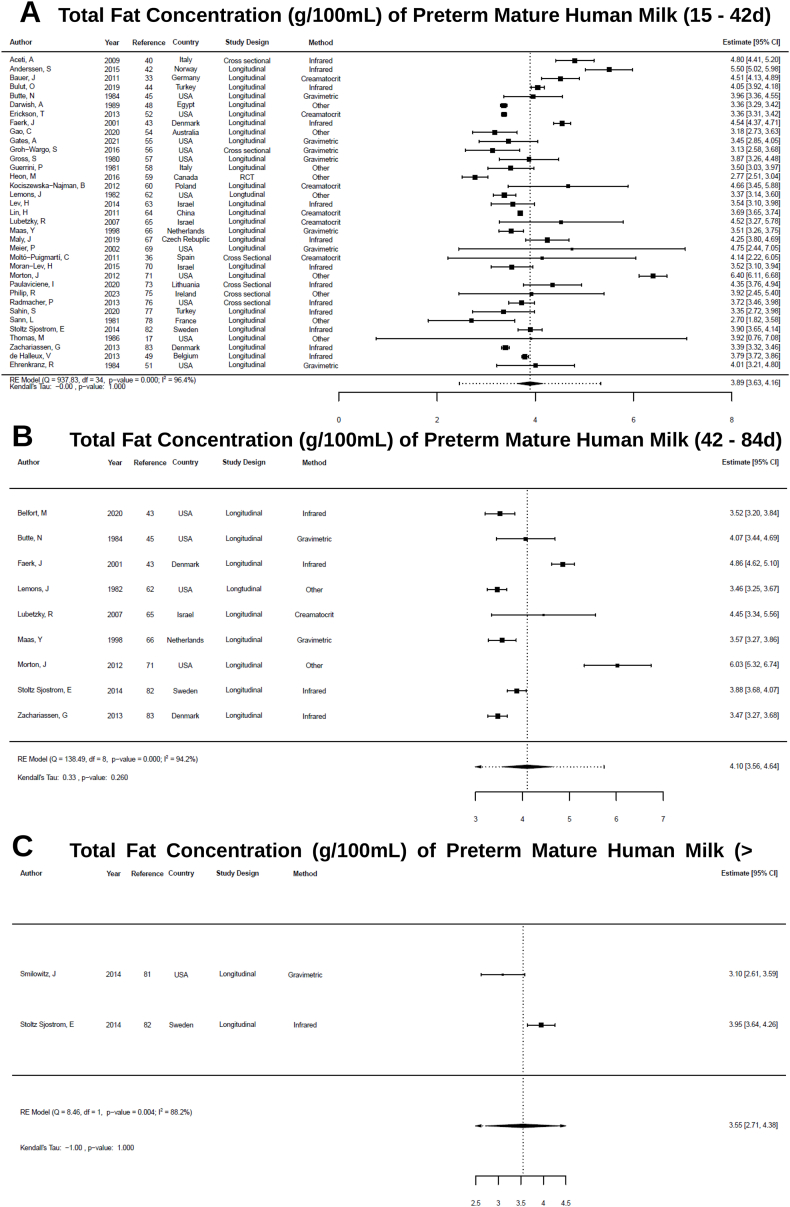
Forest plots for total fat content (g/100 mL) in mature preterm HM across time. (A) Mature HM (15–42 d), RE model (*Q* = 937.83 df = 34, *P* value < 0.001; *I*^*2*^ = 96.4%); Kendall’s Tau: 0.00, *P* value = 1.000. (B) Mature HM (43–84 d), RE model (*Q* = 138.49, df = 8, *P* value < 0.001, *I*^2^ = 94.2%); Kendall’s Tau: 0.33, *P* value = 0.260. (C) Mature HM (>84 d), RE model (*Q* = 8.46, df = 1, *P* value = 0.004, *I*^2^ = 88.2%); Kendall’s Tau: –1.00, *P* value = 1.00. CI, confidence interval; df, degrees of freedom; HM, human milk; RE, regression effects.

Figure 3 represents a total fat content in mature preterm HM across time. Panel B reports the highest fat content, 4.10 (3.56, 4.64) g/100 mL, *I*^*2*^ = 94.2%, which was observed during the 43 to 84 d postbirth phase (*n* = 9), followed by 3.89 (3.63, 4.16) g/100 mL, *I*^*2*^ = 96.4% representing 15 to 42 d postbirth (panel A; *n* = 35). The lowest total fat content occurred when preterm HM was collected >84 d postbirth (panel C) and is estimated to be 3.55 (2.71, 4.38) g/100 mL, *I*^*2*^ = 88.2%. Only 2 studies reported total fat over 84 d. Sensitivity analyses did not show any noticeable differences from the overall effect size (Supplementary Figures 1–4).

### Fatty acids

A summary of study characteristics reporting FAs (*n* = 23) can be found in Table 2 [30,31,42,50,61,[67], [68], [69], [70], [71], [72], [73], [74], [75], [76], [77], [78], [79], [80], [81], [82], [83], [84], [85]]. Among these articles reporting FAs, 7 reported FAs in colostrum (<5 d), 16 reported FA content in transition milk (5–14 d), and 18 reported FA content in mature milk (>14 d). Most studies reported data across >1 lactation phase (*n* = 13), whereas others (*n* = 10) collected data in only 1 lactation phase. The infant GA range was 24 to 36 wk, and the range in which the preterm HM was collected was 1 to 182 d. All studies that reported FA composition used gas chromatography.

**TABLE 2:** Summary of the characteristics of studies reporting fatty acids in preterm HM (*n* = 24)^1^

Author, first	Year	Country	Study design	Infants, *n*	GA, wk^2^	HM age, d^3^	HM type	Fatty acid
Arsić, A [67]	2012	Serbia	L	23	(34.0, 36.0)	(14, 28)	T, M	10:0, 12:0, 14:0, 15:0, 16:0, 16:1, 16:1-*n*9, 17:0, 18:0, 18:1, 18:2-*n*6, 18:3-*n*3, 18:3-*n*6, 20:1-*n*9, 20:2, 20:3-*n*3, 20:3-*n*6, 20:4-*n*6, 20:5-*n*3, 22:4-*n*6, 22:5-*n*3, 22:6-*n*3, 24:1, 24:1-*n*9
Aydin, I [68]	2014	Turkey	L	15	34.1	(3, 28)	C, T, M	10:0, 12:0, 14:0, 14:1, 15:0, 16:0, 16:1, 17:0, 17:1, 18:0, 18:1, 18:2-*n*6, 18:3-*n*3, 18:3-*n*6, 20:0, 20:1, 20:2, 20:3-*n*3, 20:4-*n*6, 20:5-*n*3, 22:0, 22:1*n*9, 22:6-*n*3, 23:0, 24:0, 24:1
Berenhauser, A [69]	2012	Brazil	L	10	32.3	(3, 16)	T, M	12:0, 14:0, 15:0, 16:0, 16:1, 18:0, 18:1, 18:2-*n*6, 18:3-*n*3, 20:0, 20:1-*n*9, 20:4-*n*6, 20:5-*n*3, 22:6-*n*3
Bitman, J [70]	1983	United States	L	46	(26.0, 36.0)	42	M	10:0, 12:0, 14:0, 15:0, 16:0, 16:1, 17:0, 18:0, 18:1, 20:0, 20:2, 22:5-*n*3, 22:5-*n*6
Bobiński, R [71]	2013	Poland	L	32	34.7	(6, 60)	T, M	10:0, 12:0, 14:0, 14:1, 15:0, 16:0, 16:1, 17:0, 17:1, 18:0, 18:1, 18:2-*n*6, 18:3-*n*3, 18:3-*n*6, 20:0, 20:1, 20:2, 20:3-*n*3, 20:3-*n*6, 20:4-*n*6, 20:5-*n*3, 22:0, 22:1n9, 22:5-*n*3, 22:6-*n*3, 23:0, 24:0, 24:1
Castillo, F [72]	2021	Spain	L	118	28.8	(4, 29)	C, T, M	12:0, 14:0, 16:0, 18:0, 18:1, 18:2-*n*6, 18:3-*n*3, 20:4-*n*6, 22:5-*n*3, 22:6-*n*3
De Oliveira, S [30]	2017	France	RCT	12	30	9	T	8:0, 10:0, 12:0, 14:0, 14:1-*n*5, 16:0, 18:0, 18:1, 18:2-*n*6, 18:3-*n*3, 18:3-*n*6, 20:0, 20:1-*n*9, 20:2, 20:3-*n*6, 20:4-*n*6, 20:5-*n*3, 22:0, 22:1*n*9, 22:5-*n*3, 22:6-*n*3, 24:0, 24:1-*n*9
Ehrenkranz, R [31]	1984	United States	L	21	29	(2, 42)	C, T, M	10:0, 12:0, 14:0, 16:0, 16:1-*n*9, 18:0, 18:1, 18:2-*n*6
Fares, S [73]	2018	Tunisia	CS	99	31.1	(4, 4)	C	14:0, 16:0, 16:1, 18:0, 18:1, 18:2-*n*6, 18:3-*n*3, 18:3-*n*6, 20:2, 20:3-*n*3, 20:3-*n*6, 20:4-*n*6, 20:5-*n*3, 22:5-*n*3, 22:6-*n*3
Genzel-Boroviczény, O [74]	1997	Germany	L	19	28.6	(5, 30)	T, M	10:0, 12:0, 14:0, 16:0, 18:0, 18:1, 18:2-*n*6, 20:2, 20:3-*n*3, 20:3-*n*6, 20:4-*n*6, 20:5-*n*3, 22:4-*n*6, 22:5-*n*3
Hossain, Z [75]	2016	Canada	L	32	27.8	62	M	12:0, 14:0, 16:0, 18:0, 18:1, 18:2-*n*6, 18:3-*n*3, 20:4-*n*6, 20:5-*n*3, 22:6-*n*3
Innis, S [76]	1990	Canada	L	9	29.4	14	T	10:0, 12:0, 14:0, 16:0, 18:0, 18:1, 18:2-*n*6, 18:3-*n*3, 20:4-*n*6, 20:5-*n*3, 22:4-*n*6, 22:5-*n*3, 22:6-*n*3, 8:0
Jang, S [77]	2011	Korea	L	104	31.7	(7, 84)	T, M	10:0, 12:0, 14:0, 14:1, 16:0, 16:1-*n*9, 18:0, 18:1, 18:2-*n*6, 18:3-*n*3, 18:3-*n*6, 20:0, 20:1-*n*9, 20:3-*n*3, 20:3-*n*6, 20:4-*n*6, 20:5-*n*3, 22:0, 22:1n9, 22:4-*n*6, 22:5-*n*3, 22:6-*n*3, 24:0, 24:1-*n*9
Kovács, A [78]	2005	Hungary	L	8	(23.8, 32.2)	(1, 21)	C, T, M	12:0, 14:0, 16:0, 16:1-*n*7, 18:0, 18:1-*n*7, 18:1-*n*9, 18:2-*n*6, 20:1-*n*9, 20:3-*n*3, 20:3-*n*6, 20:4-*n*6, 22:4-*n*6, 22:5-*n*3, 22:5-*n*6
Lemons, J [42]	1982	United States	L	20	33	(7, 56)	T, M	16:0, 18:0, 18:1, 18:2-*n*6
Lepage, G [79]	1984	Canada	CS	32	(26.0, 36.0)	17.5	M	10:0, 12:0, 14:0, 8:0
Luukkainen, P [80]	1994	Finland	L	23	30	(7, 182)	T, M	14:0, 16:0, 16:1, 18:0, 18:1, 18:2-*n*6, 18:3-*n*3, 20:3-*n*6, 20:4-*n*6, 20:5-*n*3, 22:5-*n*3, 22:6-*n*3
Maas, C [81]	2017	Germany	CS	35	(25.4, 32.0)	27.5	M	12:0, 14:0, 16:0, 16:1-*n*7, 18:0, 18:1, 18:2-*n*6, 20:0, 20:3-*n*6, 20:4-*n*6, 20:5-*n*3, 22:0, 22:4-*n*6, 22:5-*n*3
Marc, I [82]	2011	Canada	L	24	27.7	49	M	10:0, 12:0, 14:0, 14:1-*n*5, 16:0, 16:1-*n*7, 18:0, 18:1-*n*7, 18:1-*n*9, 18:2-*n*6, 18:3-*n*3, 20:1-*n*9, 20:2-*n*6, 20:3-*n*6, 20:4-*n*6, 22:5-*n*3, 22:6-*n*3
Moltó-Puigmartí, C [50]	2011	Spain	CS	20	30.1	(3, 30)	C, T, M	10:0, 12:0, 14:0, 14:1, 15:0, 16:0, 16:1-*n*7, 16:1-*n*9, 17:0, 17:1, 18:0, 18:1, 18:1-*n*7, 18:1-*n*9, 18:2-*n*6, 18:3-*n*6, 20:0, 20:1-*n*9, 20:2-*n*6, 20:3-*n*6, 20:4-*n*6, 22:0, 22:1n9, 22:5-*n*3, 24:0, 24:1, 8:0
Nilsson, A [83]	2018	Sweden	CS	78	25.5	(7, 102)	T, M	12:0, 14:0, 14:1-*n*5, 15:0, 16:0, 16:1-*n*7, 17:0, 18:0, 18:1-*n*7, 18:1-*n*9, 18:2-*n*6, 20:0, 20:3-*n*3, 20:3-*n*6, 22:0, 23:0, 24:0
Sabel, K [84]	2009	Sweden	CS	51	(24.0, 36.0)	7	T	12:0, 14:0, 16:0, 16:1, 18:0, 18:1, 18:2-*n*6, 18:3-*n*3, 18:3-*n*6, 20:3-*n*6, 20:4-*n*6, 20:5-*n*3, 22:6-*n*3, 24:1, 24:1-*n*9
Silber, G [61]	1988	United States	L	5	29.6	(0, 4)	C	10:0, 12:0, 14:0

Abbreviations: C, colostrum; CS, cross-sectional; GA, gestational age; HM, human milk; L, longitudinal; M, mature; RCT, randomized controlled trial; T, transition.

1 Values reported as mean or (range). Gas chromatography was used to quantify fatty acids.

2 Mean gestational age. Parentheses indicate upper and lower ranges.

3 Mean age of HM was collected. Parentheses indicate upper and lower ranges.

**FIGURE 4:**
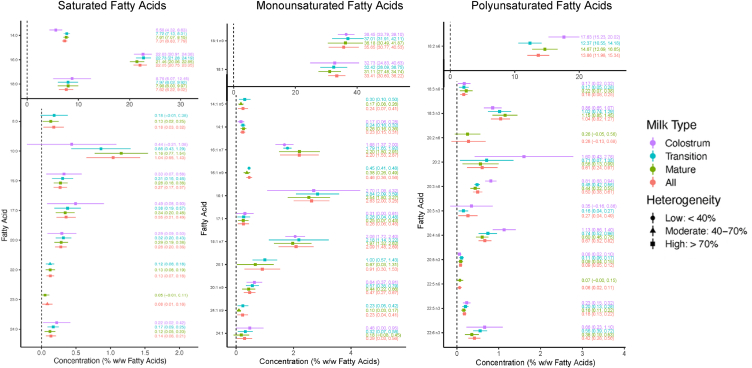
Summary of individual fatty acid (% w/w) forest plots across milk type presented by fatty acid classification (SFA, MUFA, and PUFA). Milk type was denoted by color: colostrum (), transition (), mature (), and all ().

Figure 4 presents FA concentrations in preterm HM, separated by class, milk type, and arranged from lowest to highest across 3 panels (A–C). A total of 11 SFA, 6 MUFA, and 10 PUFA are reported, with an additional 7 unsaturated FAs that did not specify double bond location. Most FAs reported exhibited high heterogeneity across studies when stratified by milk type.

Panel A shows SFA; although the majority of SFAs were below a concentration of 1.5%, 16:0 (palmitic acid) had the highest concentration with 22.1% (colostrum = 22.6%, transition = 22.7%, and mature = 21.5%) in total preterm HM. This was followed by 18:0 (stearic acid) with 7.6% (colostrum = 8.8%, transition = 8.0%, and mature = 8.0%), and 14:0 (myristic acid) at a concentration of 7.3% (colostrum = 5.6%, transition = 7.7%, and mature = 7.3%), in total preterm HM.

Panel B illustrates MUFAs that were analyzed in this meta-analysis. Concentration varied widely, from 0.2% to 33.4%. The highest concentration was seen with FA 18:1*n*9 (oleic acid), at a concentration of 35.7% (colostrum = 36.4%, transition = 37.0%, and mature = 36.2%) in total preterm HM. Few studies also reported 18:1 without any distinguishing isomers. This was reported as a total concentration of 33.4% (colostrum = 32.7%, transition = 32.4%, and mature = 31.1%) in total preterm HM.

Panel C displays pooled concentrations of PUFAs in preterm HM. The highest concentration was observed for 18:2*n*6 (LA) with a concentration of 13.7% (colostrum = 17.6%, transition = 12.4%, and mature = 14.7%) in preterm HM. Other notable FA concentrations were 20:4*n*6 (ARA) with a concentration of 0.7% (colostrum = 1.1%, transition = 0.7%, and mature = 0.6%), and 18:3*n*3 (ALA) with a concentration of 1.0% (colostrum = 0.9%, transition = 1.0%, and mature = 1.2%) in preterm HM. Other minor FAs detected at low levels were the long-chain PUFAs (LCPUFA) 20:5*n*3 (EPA) with a concentration of 0.1% (colostrum = *undetected,* transition = 0.1%, and mature = 0.1%), and 22:6*n*3 (DHA) with a concentration of 0.4% (colostrum = 0.7%, transition = 0.6%, and mature = 0.4%) in preterm HM. Complete estimates and forest plots for all FAs are included in Supplementary Figure 5.

### Total fat content by analytical methodology

Differences were observed across the type of analytical method used to quantify total fat in preterm HM. The infrared (*n* = 18 studies) analysis method produced the highest mean estimate for total fat [3.92 (3.65, 4.20) g/100 mL], followed by creamatocrit [*n* = 7; 3.86 (3.46, 4.26) g/100 mL] in preterm HM. Gravimetric (*n* = 11) and other/nonspecified (*n* = 14) methods produced the lowest total fat values albeit were very similar [3.58 (3.31, 3.85) and 3.59 (3.07, 4.10) g/100 mL], respectively. Table 3 provides total fat content across methods and milk types.

**TABLE 3:** Total fat composition in preterm human milk meta-analysis results stratified by analytical method and milk type

Method	Milk type	Estimate^1^	SE^1^	95% Confidence interval		Cochran’s *Q*	df	*P* value^2^	*I*^2^	Kendall’s Tau	*P* value^3^
				Lower^1^	Upper^1^						
Gravimetric											
	Colostrum	2.95	0.67	1.63	4.27	105	3	<0.001	97.2	0	1
	Transition	3.61	0.13	3.35	3.87	12	6	0.073	48.1	–0.24	0.562
	Mature	3.58	0.14	3.31	3.85	13	7	0.065	47.5	0.29	0.399
Infrared											
	Colostrum	3.08	0.48	2.14	4.01	33	2	<0.001	94	0.33	1
	Transition	3.81	0.19	3.44	4.18	157	10	<0.001	93.7	0.16	0.542
	Mature	3.99	0.15	3.69	4.28	163	14	<0.001	91.4	0.16	0.435
Creamatocrit											
	Colostrum	3.36	0.11	3.15	3.58	0.005	1	0.945	0	1	1
	Transition	5.11	0.22	4.67	5.54	3	2	0.178	42.1	–0.33	1
	Mature	4.00	0.26	3.49	4.5	111	5	<0.001	95.5	–0.33	0.469
Other/not-specified											
	Colostrum	2.99	0.5	2	3.97	129	5	<0.001	96.1	0.2	0.719
	Transition	3.52	0.24	3.05	3.99	162	11	<0.001	93.2	0.06	0.841
	Mature	3.65	0.4	2.87	4.43	200	8	<0.001	96	0.28	0.358

Abbreviation: df, degrees of freedom.

1 Estimates are presented as g/100 mL.

2 Cochran’s Q test for heterogeneity.

3 Begg’s rank correlation test for publication bias.

## Discussion

In the present study, SFAs and **MUFAs** were observed to comprise ∼39% and 44% of total FAs, respectively, which is similar to previous reports that SFAs and MUFAs make up ∼40% to 45% of FAs in preterm milk [1,3]. Similarly, PUFAs were observed to represent ∼18% of total FAs, which is also within the range (15%–20%) previously reported. With the variance in total fat content of HM, it is critical to better characterize how fat and FA content vary throughout lactation to help inform the standard of care for premature infants and to help support optimal development, especially since preterm infants are often born before they have had the opportunity to accrete FAs that are critical to their neural and visual development.

### Total fat

Total fat content of HM is variable over the course of a single feed and fluctuates over a 24-h period. Additionally, it increases in the first couple of weeks of lactation and then stabilizes throughout the remainder of milk production. The mean total fat content of preterm HM has previously been estimated to contain 3.5 to 4.5 g lipids/100 mL with evidence to suggest variability outside of this range [2]. The present analysis found that transition and mature preterm HM fell within this range (mean estimates of 3.74 and 3.85 g/100 mL, respectively); however, colostrum total fat content of preterm HM was observed to steadily increase across lactation stages, with colostrum having the lowest fat content and mature preterm HM having the highest. This agrees with previous reviews and meta-analyses of preterm HM fat content [7,8], which is expected as the increase in fat content corresponds to the increased energy needs of the growing infant.

There was considerable heterogeneity in the studies included in the present meta-analysis, with several studies reflecting wide confidence intervals. The largest of which was a study by Thomas et al. [64], in which fore- and hind-milk samples across morning, noon, and evening were collected. As fat content is known to vary widely according to the time of day and time at which it was collected during the expression, this may help to explain some of the variability and heterogeneity observed in total fat content. Additionally, sample handling and collection techniques (e.g., if/how the milk sample was mixed before analysis, whether the sample represented a full breast expression, and/or if the sample was collected before or after a feed) impact the amount of fat available for analysis [86,87], which may further enhance the variability observed. Because the lipid and aqueous components of HM separate during storage, appropriate mixing (e.g., ultrasonic homogenizer or vortex before fat analysis) is necessary to provide accurate results. For example, studies that do not report mixing HM before analysis can see ≥5% difference in fat content, which is likely due to inadequate mixing [86,88]. Furthermore, the FA content of preterm HM, which is discussed in more detail below, is particularly sensitive to maternal diet [89], which likely also helps to explain the high heterogeneity observed in the current analysis.

### SFAs

Palmitic acid (16:0) is the most abundant SFA in HM. In preterm HM, the majority of palmitic acid is esterified at the sn-2 position of triacylglycerols [90]. This specific configuration facilitates its absorption as 2-monoacylglycerol, which prevents the formation of insoluble calcium soaps, which enhances calcium absorption [91]. Additionally, the sn-2 esterification is more compatible with the limited activity of pancreatic lipase in preterm infants, promoting efficient digestion and the release of FAs and monoacylglycerols that are readily absorbed and utilized as an energy source [92].

Stearic acid (18:0) remains fairly consistent across lactation stages, with total stearic acid concentration at 7.62% w/w across all milk types. Interestingly, both myristic (14:0) and capric (10:0) acids were lowest in colostrum, increasing during transition and mature milk production from 5.58 to 7.61 and from 0.44% to 1.16% w/w for myristic and capric, respectively. As these FAs are produced primarily in the mammary gland, the increase may be reflective of further development of the mammary gland to allow for increased total fat incorporation into milk over time [10,93].

### MUFAs

Oleic acid (18:1) is the most abundant FA in HM [11], which was also true across colostrum, transition, and mature milk analyses in the present study. Oleic acid may be particularly important for fluidity properties of milk fat globules, allowing for lipid transport to baby [14]. Although oleic acid provides an available source of energy, other functions of the FA during infancy are not well understood [94,95]. The concentration of oleic acid remained relatively consistent over time in the present study, with total oleic acid across all milk types reported at 35.7% w/w. Following oleic acid, palmitoleic acid (16:1*n*-7) and vaccenic acid (18:1*n*-7) were the next most abundant MUFAs, each contributing ∼2% w/w across all milk types.

### PUFAs and LCPUFAs

The essential PUFAs LA (18:2*n*–6) and ALA (18:3*n*–3) are the most abundant omega-6 (*n*–6) and ω-3 FAs in HM, with LA being the most abundant compared with ALA. In the present analysis, LA was highest in colostrum (17.6% w/w) and relatively stabilized across transition and mature milk stages (12.4% to 14.7% w/w, respectively). Conversely, ALA was lowest in colostrum (0.9% w/w) and increased across the remaining stages of lactation (1.0% and 1.2% w/w). It should be noted, however, the ALA had the largest interstudy variability of the colostrum values across all PUFAs, which may impact the interpretation of the estimate as future research is published. Additionally, the variability seen in these FAs and their metabolites may also be attributed to the influence of maternal nutrition, with differences in their concentrations due to higher and more consistent fatty fish intake in some populations and poorer diet fat quality in others [89].

The LCPUFA metabolites, ARA (20:4*n*–6) and DHA (22:6*n*–3), were both highest in colostrum and then decreased over time; EPA (20:5*n*–3) was undetected in colostrum in the samples included in the present analysis, but its concentration was stable across transition and mature stages. As LA and ALA compete for desaturation and elongation, their metabolites are impacted by the proportional availability in the diet. The higher proportion of DHA and ARA observed in colostrum may correspond to their important roles in immune function and development as well as nervous system and brain development. As a result of the evidence for the critical role of DHA and ARA in these domains, experts recommend that preterm infants receive DHA in a range of 0.5% to 1.0% of total FAs with a corresponding DHA to ARA ratio of 0.5 to 1.0. On the basis of the results of the present analysis, only colostrum and transition milk provide DHA and ARA at the lower end of this range, with mature preterm HM being observed as inadequate to provide a sufficient amount of DHA and ARA for optimal development. Increased volumes of HM may help reach some recommendations for these FAs; however, this has to be tailored to the infant based on their body weight, which impacts the feeding volume they can tolerate. Although intravenous lipids may be 1 option to meet these goals, enteral feeding is preferred; thus, using products such as HM fortifiers or modulars allows for targeted nutrition to provide additional nutrients like DHA and ARA to these infants to support their growth and development while also staying within a tolerable feeding volume.

A preferential increase in the LCPUFA C20:2*n*6 (eicosadienoic acid) was also observed in this analysis. Positive associations between eicosadienoic acid and birth weight as well as GA have been reported. There is also a positive correlation between growth velocity in preterm infants and eicosadienoic acid in breast milk [96]. This may be attributed to the apparent role of *n*–6 FAs in mitigating inflammation and protecting against intestinal injury [95].

### Total fat by analytical methodology

The forest plots examining total fat by analytical methodology are located in Supplementary Figures 6 to 9. The infrared method provided the highest estimate of total fat content of preterm HM followed by creamatocrit and gravimetric, respectively. The gravimetric method is considered the method of reference for analyzing HM fat [97]. Most studies using the gravimetric method utilized Mojonnier or Roese–Gottleib extraction methods. Creamatocrit has been noted to become less reliable when HM has been stored for long periods and when the composition is <2 g/dL [98]. Infrared has been reported to exhibit strong agreement with the gravimetric reference method when samples are appropriately homogenized and when sample volumes are >4 mL [98]. In the present analysis, the “Other/Not-Specified” methods provided the widest confidence interval (Supplementary Figure 9). However, it is important to note that the estimates provided by the subanalysis of all 3 methodologies were within the range that has previously been reported in the literature for preterm HM fat content of 3.5 to 4.5 g/100 mL.

### Strengths and limitations

Some limitations were observed among the studies included in this systematic review and meta-analysis. First, we were unable to distinguish total fat and FA concentrations across the feeding cycle due to inconsistent reporting of the time of day the HM was collected. Most studies, when reported, analyzed 24-h pooled samples, which do not capture fat and FA changes that occur during the transition from foremilk to hind-milk. These data are important as fat typically increases during the end of a feeding cycle. Next, the lack of details regarding the storage methods of HM before analyses is another limiting factor which can impact the stability and quality of total fat and FAs. For example, total fat can decrease with prolonged storage (4°C for >28 d and freezing ≤90 d), pasteurization, and repeated freeze-thaw cycles; however, the main driver appears to be inadequate mixing before analysis [99,100]. Interestingly, FAs do not appear to be impacted [99]. Additionally, a wide variation among analytical methods used to quantify total fat and FAs was found. We observed a higher total fat concentration when the creamatocrit method was used compared with infrared and gravimetric methods. The gravimetric method has produced lower amounts of total fat in other studies that may not be reflective of a true reduction in the HM sample. Fat globules can also adhere to the surface of the collection containers, and the HM must be homogenized sufficiently before testing [99]. Many studies did not report the methods used to mix the HM samples. If so, the description was often vague (e.g., “well mixed then centrifuged” [21], used a “domestic blender” [65], and “mixing and sonication” [23]). Thus, we did not report the methods used to mix the samples. Also, reporting percent of total FAs can be highly variable depending on the type and number of FAs being included in the analyses. Many studies also did not report GA or the age at which the HM sample was collected. Other studies found that fat content was higher in very preterm HM, especially in colostrum and transition HM, compared with preterm samples, and fat decreased with GA [50]. Also, maternal characteristics, such as BMI and income, which can impact fat composition, were often missing. Previous studies found that mothers with a higher BMI had higher total HM fat and higher proportions of SFAs and lower DHA [101]. Furthermore, this analysis only used studies that provided fat and FA estimates across countries with an HDI > 0.8, so this analysis may not be representative of the nutritional composition of HM from women in developing countries. In addition, maternal diet was not addressed in the studies included in the present study, which also impacts the total fat and FA composition of HM [102]. Although studies from developed countries were used to minimize maternal undernourishment or malnourishment, without true dietary intake data, the certainty of this statement cannot be upheld. Lastly, the quality of studies in this systematic review was not determined as poor, fair, or good because the assessment tools included questions of exposure, which very few studies included in the present analysis.

In conclusion, current nutrient recommendations for preterm infants include targeted levels for total lipids, LA, ALA, DHA, and DHA to ARA ratio to support neurodevelopment and optimal growth. The results of the present analysis reflect that transition and mature preterm HM are within the current expert guidelines for total fat content. Conversely, the present analysis reflects that DHA and ARA meet expert guidance for colostrum only. Coupling these data with current preterm nutrient recommendations allows clinicians to develop more informed feeding plans.

## Author contributions

The authors’ responsibilities were as follows – DCM, ADLB, TMB: designed the research (project conception, development of overall research plan, and study oversight); DCM, MAP: conducted the research (hands-on conduct of the experiments and data collection) and provided essential databases necessary for the research; DCM: analyzed data or performed statistical analysis and primary responsible for final content; DCM, MAP, VKT, MFN, ADLB: wrote paper (only authors who made a major contribution); DLC: provided analytical methodology expertise; DLC, MFN, JNK, TMB, KEN, ADLB: provided technical expertise; and all authors: read and approved the final manuscript.

## Data availability

Data described in the manuscript, code book, and analytic code will be made available on request pending application and approval.

## Declaration of Generative AI and AI-Assisted Technologies in the Writing Process

The authors declare that no generative AI or AI-assisted technologies were used in the writing of this manuscript.

## Funding

Project partially funded by Mead Johnson Nutrition. ADLB, TMB, DLC, JNK, and MFN are employees of Mead Johnson Nutrition. DCM and MAP received funding from Mead Johnson Nutrition to perform the methods and results. The employees of Mead Johnson Nutrition provided input on the study objectives, interpretation of results, and final manuscript, but they did not provide input on the data extraction or statistical analysis.

## Conflict of interest

DCM and MAP report that financial support and writing assistance were provided by Mead Johnson Nutrition. DCM reports a relationship with Mead Johnson Nutrition that includes: consulting or advisory and funding grants. ADLB, DLC, JNK, MFN, KEN, and TMB report a relationship with Mead Johnson Nutrition that includes: employment. Consultant for Reckitt | Mead Johnson as an independent contractor on a separate project—DCM. If there are other authors, they declare that they have no known competing financial interests or personal relationships that could have appeared to influence the work reported in this paper.
