# Supplementary material for: Total Fat and Fatty Acid Composition of Preterm Human Milk: A Systematic Review and Meta-analysis
Source: Adv Nutr. 2026 May 26;17(7):100661. doi: 10.1016/j.advnut.2026.100661 (PMC13315656; doi:10.1016/j.advnut.2026.100661)

Total Fat and Fatty Acid Composition of Preterm Human Milk: A Systematic Review and Meta- Analysis

Corresponding Author, Ariana Bailey

Supplementary Appendices

Contents

Supplementary Table 1. Prisma 2020 Checklist 3

Supplementary Table 2. Data Handling Steps 4

Supplementary Table 3. Example conversion factors and calculations 5

Supplementary Figure 1. Sensitivity Analysis, Fat Concentration of All Milk Types 6

Supplementary Figure 2. Sensitivity Analysis, Fat Concentration, Colostrum 7

Supplementary Figure 3. Sensitivity Analysis, Fat Concentration, Transition 8

Supplementary Figure 4. Sensitivity Analysis, Fat Concentration, Mature 9

Supplementary Figure 5. Fatty Acid Forest Plots, w/w% concentration in preterm human milk 10

8:0 10

10:0 11

12:0 12

14:0 13

14:1 14

14:1-n5 15

15:0 16

16:0 17

16:1 18

16:1n-7 19

16:1n-9 20

17:0 21

17:1 22

18:0 23

18:1 24

18:1n-7 25

18:1n-9 26

18:2n-6 27

18:3n-3 28

18:3n-6 29

20:0 30
20:1 31
20:1n-9 32
20:2 33
20:2 n-6 34
20:3 n-3 35
20:3 n-6 36
20:4 n-6 37
20:5 n-3 38
22:0 39
22:1 n-9 40
22:5 n-3 41
22:5 n-6 42
22:6 n-3 43
23:0 44
24:0 45
24:1 46
24:1 n-9 47
Supplementary Figure 6. Fat Analysis by Method, Creamatocrit 48
Supplementary Figure 7. Fat Analysis by Method, Gravimetric 48
Supplementary Figure 8. Fat Analysis by Method, Infrared 50
Supplementary Figure 9. Fat Analysis by Method, Other 51

Total Fat and Fatty Acid Composition of Preterm Human Milk: A Systematic Review and Meta- Analysis

Corresponding Author, Ariana Bailey

Supplementary Table 1. Prisma 2020 Checklist

Section and Topic	Item #	Checklist item	Location where item is reported
**TITLE**			1
Title	1	Identify the report as a systematic review.	
**ABSTRACT**			
Abstract	2	See the PRISMA 2020 for Abstracts checklist.	1-2
**INTRODUCTION**			
Rationale	3	Describe the rationale for the review in the context of existing knowledge.	3
Objectives	4	Provide an explicit statement of the objective(s) or question(s) the review addresses.	3-4
**METHODS**			
Eligibility criteria	5	Specify the inclusion and exclusion criteria for the review and how studies were grouped for the syntheses.	5
Information sources	6	Specify all databases, registers, websites, organisations, reference lists and other sources searched or consulted to identify studies. Specify the date when each source was last searched or consulted.	4-5
Search strategy	7	Present the full search strategies for all databases, registers and websites, including any filters and limits used.	4-5
Selection process	8	Specify the methods used to decide whether a study met the inclusion criteria of the review, including how many reviewers screened each record and each report retrieved, whether they worked independently, and if applicable, details of automation tools used in the process.	4-5
Data collection process	9	Specify the methods used to collect data from reports, including how many reviewers collected data from each report, whether they worked independently, any processes for obtaining or confirming data from study investigators, and if applicable, details of automation tools used in the process.	4-5
Data items	10a	List and define all outcomes for which data were sought. Specify whether all results that were compatible with each outcome domain in each study were sought (e.g. for all measures, time points, analyses), and if not, the methods used to decide which results to collect.	4-5
	10b	List and define all other variables for which data were sought (e.g. participant and intervention characteristics, funding sources). Describe any assumptions made about any missing or unclear information.	4-5<br>Appendix 2
Study risk of bias assessment	11	Specify the methods used to assess risk of bias in the included studies, including details of the tool(s) used, how many reviewers assessed each study and whether they worked independently, and if applicable, details of automation tools used in the process.	5-6
Effect measures	12	Specify for each outcome the effect measure(s) (e.g. risk ratio, mean difference) used in the synthesis or presentation of results.	6
Synthesis methods	13a	Describe the processes used to decide which studies were eligible for each synthesis (e.g. tabulating the study intervention characteristics and comparing against the planned groups for each synthesis (item #5)).	6
	13b	Describe any methods required to prepare the data for presentation or synthesis, such as handling of missing summary statistics, or data conversions.	5-6<br>Appendix 2
	13c	Describe any methods used to tabulate or visually display results of individual studies and syntheses.	6
	13d	Describe any methods used to synthesize results and provide a rationale for the choice(s). If meta-analysis was performed, describe the model(s), method(s) to identify the presence and extent of statistical heterogeneity, and software package(s) used.	6
	13e	Describe any methods used to explore possible causes of heterogeneity among study results (e.g. subgroup analysis, meta-regression).	6
	13f	Describe any sensitivity analyses conducted to assess robustness of the synthesized results.	6
Reporting bias assessment	14	Describe any methods used to assess risk of bias due to missing results in a synthesis (arising from reporting biases).	N/A
Certainty assessment	15	Describe any methods used to assess certainty (or confidence) in the body of evidence for an outcome.	N/A
**RESULTS**			
Study selection	16a	Describe the results of the search and selection process, from the number of records identified in the search to the number of studies included in the review, ideally using a flow diagram.	6-7 and Figure 1
	16b	Cite studies that might appear to meet the inclusion criteria, but which were excluded, and explain why they were excluded.	Figure 1
Study characteristics	17	Cite each included study and present its characteristics.	Tables 1 & 2
Risk of bias in studies	18	Present assessments of risk of bias for each included study.	6, and Figures 2 & 3
Results of individual studies	19	For all outcomes, present, for each study: (a) summary statistics for each group (where appropriate) and (b) an effect estimate and its precision (e.g. confidence/credible interval), ideally using structured tables or plots.	Figures 2-4
Results of syntheses	20a	For each synthesis, briefly summarise the characteristics and risk of bias among contributing studies.	6, and Tables 1 & 2
	20b	Present results of all statistical syntheses conducted. If meta-analysis was done, present for each the summary estimate and its precision (e.g. confidence/credible interval) and measures of statistical heterogeneity. If comparing groups, describe the direction of the effect.	6, and Figures 2 & 3
	20c	Present results of all investigations of possible causes of heterogeneity among study results.	6, and Figures 2 & 3
	20d	Present results of all sensitivity analyses conducted to assess the robustness of the synthesized results.	Appendices 8-11
Reporting biases	21	Present assessments of risk of bias due to missing results (arising from reporting biases) for each synthesis assessed.	6, and Figures 2 & 3
Certainty of evidence	22	Present assessments of certainty (or confidence) in the body of evidence for each outcome assessed.	6, and Figures 2 & 3
**DISCUSSION**			
Discussion	23a	Provide a general interpretation of the results in the context of other evidence.	11-15
	23b	Discuss any limitations of the evidence included in the review.	15-16
	23c	Discuss any limitations of the review processes used.	15-16
	23d	Discuss implications of the results for practice, policy, and future research.	16
**OTHER INFORMATION**			
Registration and protocol	24a	Provide registration information for the review, including register name and registration number, or state that the review was not registered.	2
	24b	Indicate where the review protocol can be accessed, or state that a protocol was not prepared.	2
	24c	Describe and explain any amendments to information provided at registration or in the protocol.	Online at PROSPERO
Support	25	Describe sources of financial or non-financial support for the review, and the role of the funders or sponsors in the review.	17
Competing interests	26	Declare any competing interests of review authors.	17
Availability of data, code and other materials	27	Report which of the following are publicly available and where they can be found: template data collection forms; data extracted from included studies; data used for all analyses; analytic code; any other materials used in the review.	17

Supplementary Table 2. Data Handling Steps

Outcome	Statistic	Preferred	If missing
Fat and Fatty Acids	Location	Mean	1. Used median if reported<br>2. Otherwise exclude
	Sample Size	n (HM samples)	1. Used n (infants) if reported<br>2. Otherwise exclude
	Variability	SD	1. Calculate SD from SEM<br>2. Estimate SD from min and max<br>3. Impute missing SD from available studies
Gestational Age			
	Location	Mean	1. Use median if reported<br>2. Mean of min and max values
Age at collection			
	Location	Mean	1. Use median if reported<br>2. Mean of min and max values

HM: Human Milk

Statistic denotes the class of output summary statistics from the studies included for analysis

Supplementary Table 3. Example conversion factors and calculations

Authors	Year	Total Fat	Units	Conversion	Total Fat, g/100mL
Lin, H. Y.	2011	5.98	% CREAMATOCRIT	(x - 0.59)/1.46	3.69
Campbell-Yeo, M.	2010	4.15	G/100G	1.03	4.27
Kreissl, A.	2016	3.1	G/100ML	1	3.1
Maas, Y.G.	1998	35.8	G/KG	0.103	3.69
Morton, J.	2012	51.8	G/L	0.1	5.18
Heon, M.	2016	36.4	MG/ML	0.1	3.64

Supplementary Figure 1. Sensitivity Analysis, Fat Concentration of All Milk Types

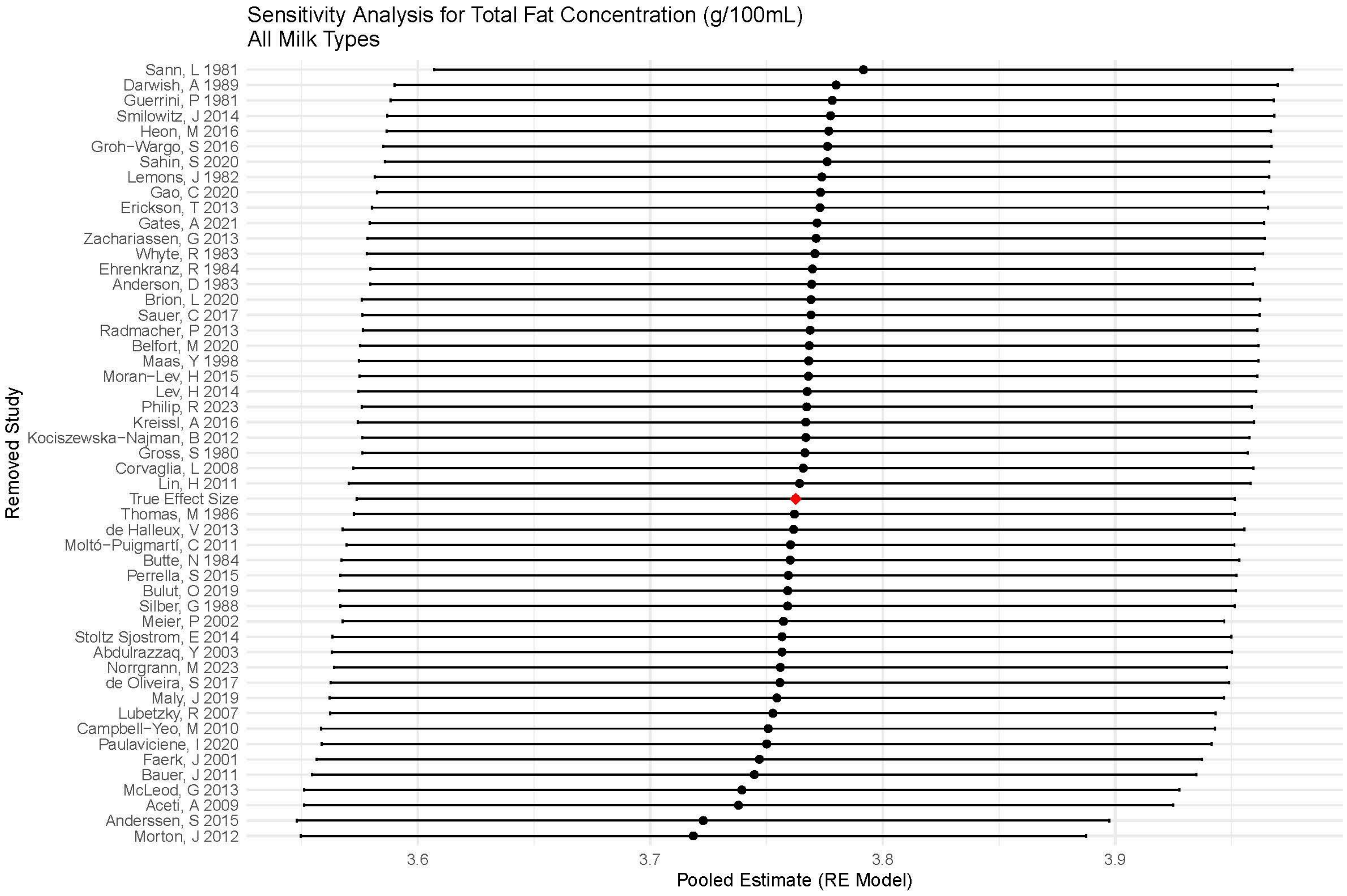

Supplementary Figure 2. Sensitivity Analysis, Fat Concentration, Colostrum

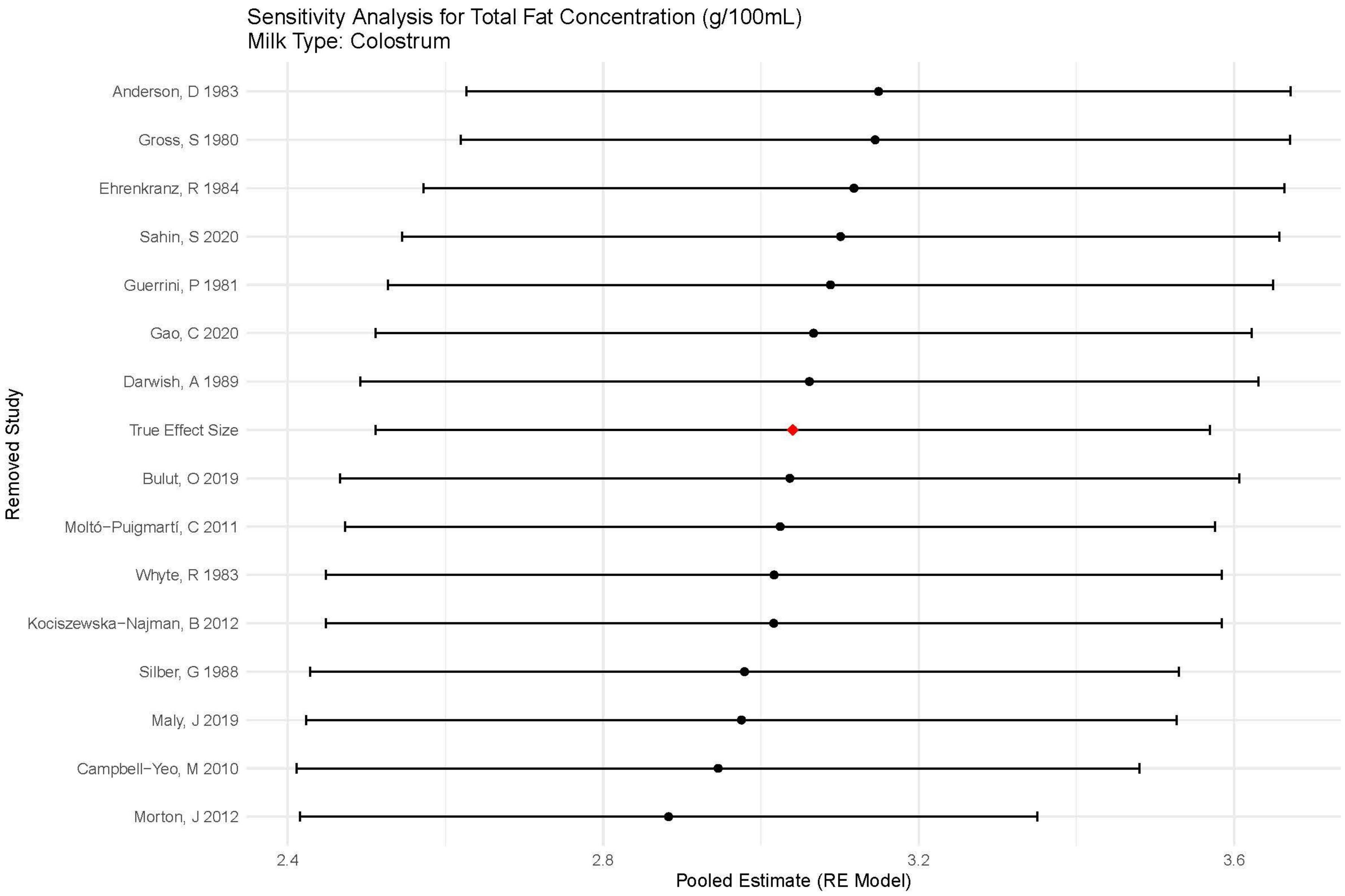

Supplementary Figure 3. Sensitivity Analysis, Fat Concentration, Transition

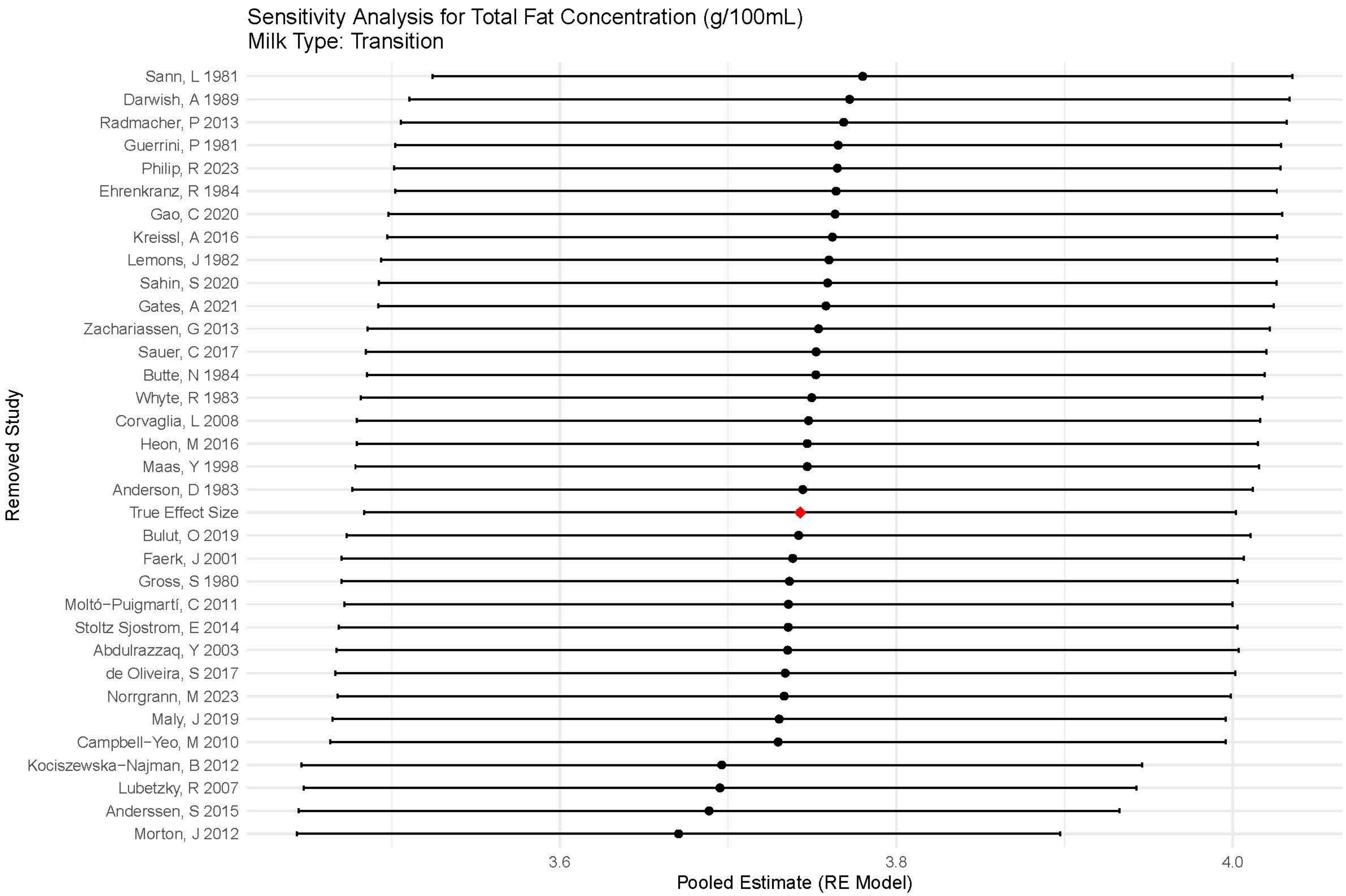

Supplementary Figure 4. Sensitivity Analysis, Fat Concentration, Mature

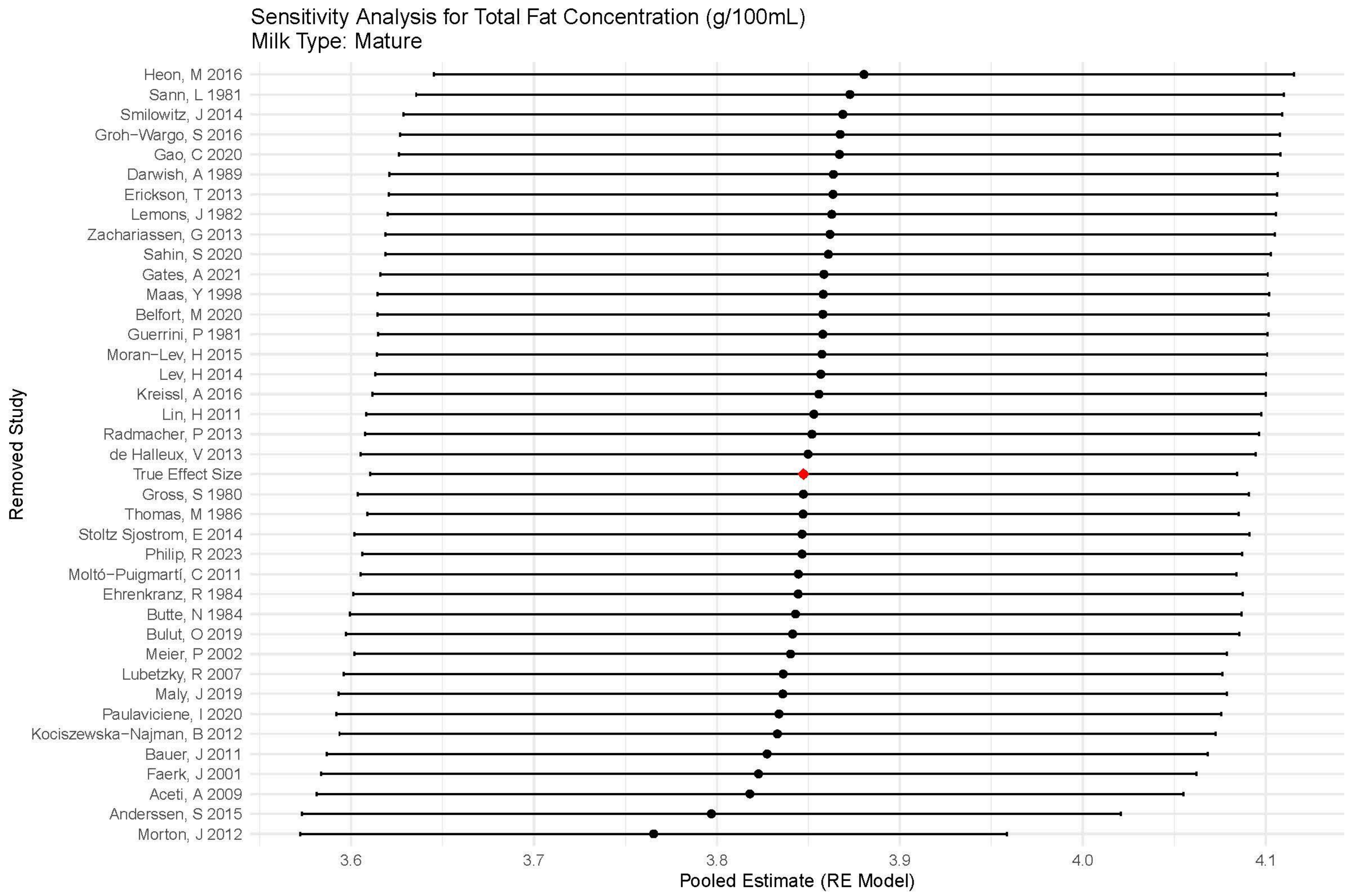

Supplementary Figure 5. Fatty Acid Forest Plots, w/w% concentration in preterm human milk

8:0

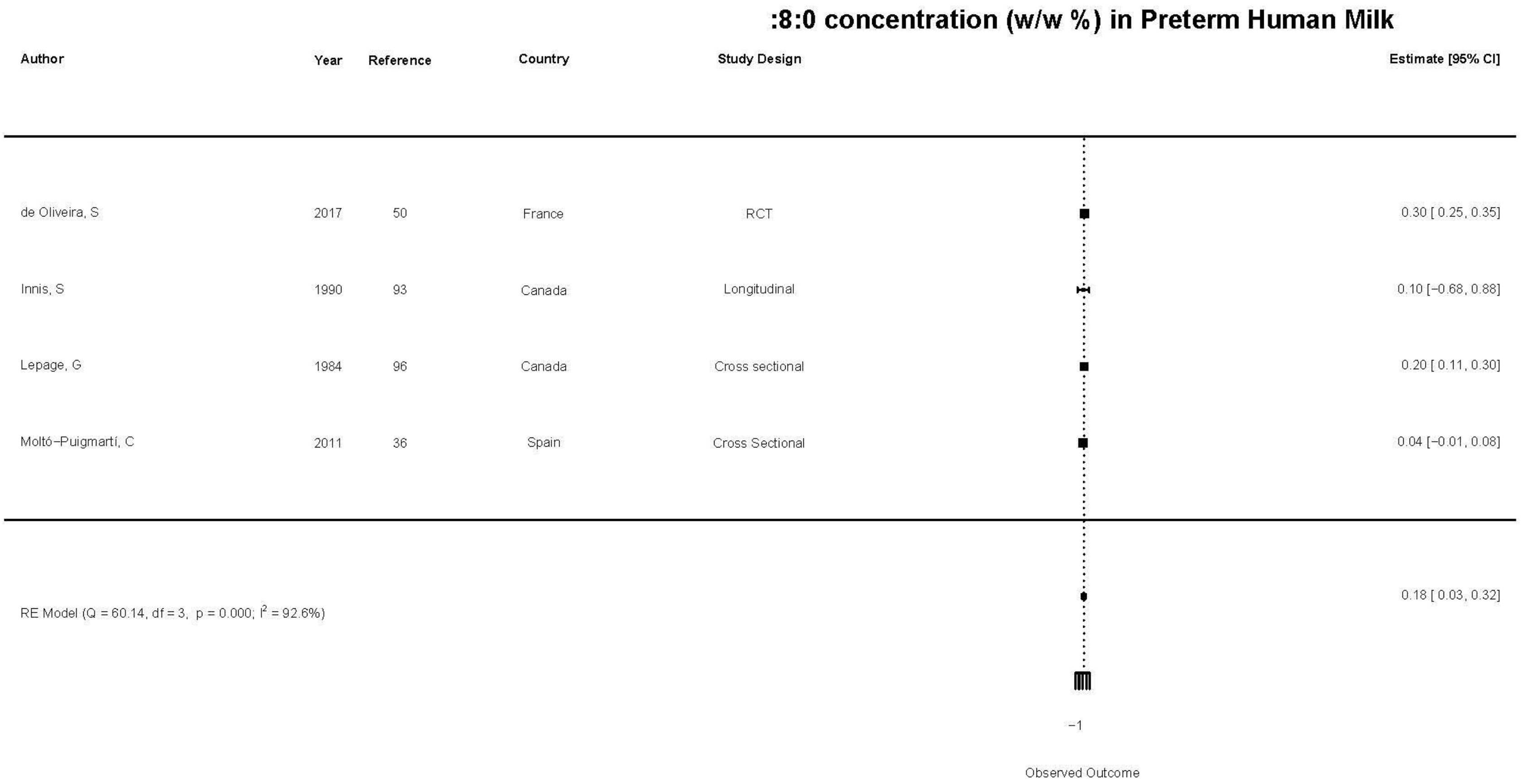

10:0

:10:0 concentration (w/w %) in Preterm Human Milk

Author	Year	Reference	Country	Study Design	Estimate [95% CI]
Arsic, A	2012	84	Serbia	Longitudinal	0.84 [0.34, 1.34]
Aydin, I	2014	85	Turkey	Longitudinal	0.11 [−0.10, 0.31]
Bitman, J	1983	87	USA	Longitudinal	1.32 [1.06, 1.59]
Bobinski, R	2013	88	Poland	Longitudinal	0.68 [0.07, 1.29]
de Oliveira, S	2017	50	France	RCT	1.80 [1.70, 1.90]
Ehrenkranz, R	1984	51	USA	Longitudinal	0.19 [−0.83, 1.20]
Genzel−Boroviczeny, O	1997	91	Germany	Longitudinal	0.96 [0.47, 1.44]
Innis, S	1990	93	Canada	Longitudinal	0.30 [0.14, 0.46]
Jang, S	2011	94	Korea	Longitudinal	1.63 [1.25, 2.02]
Lepage, G	1984	96	Canada	Cross sectional	2.41 [2.29, 2.54]
Marc, I	2011	99	Canada	Longitudinal	1.00 [0.75, 1.25]
Moltó−Puigmartí, C	2011	36	Spain	Cross Sectional	0.37 [−0.14, 0.89]
Silber, G	1988	80	USA	Longitudinal	1.48 [1.09, 1.86]
RE Model ($Q = 689.84$, $df = 12$, $p = 0.000$; $I^2 = 97.2\%$)					1.04 [0.65, 1.43]

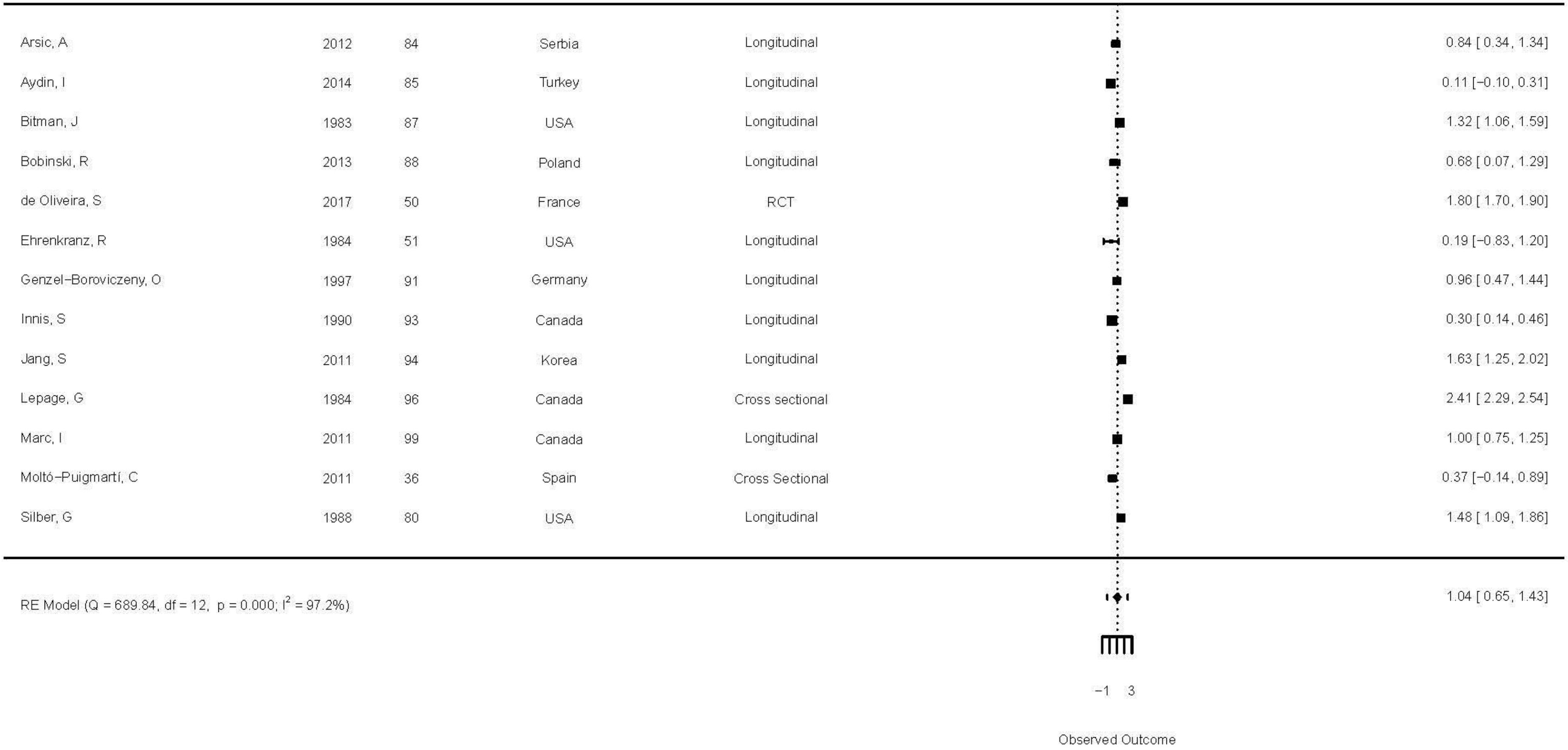

12:0

:12:0 concentration (w/w %) in Preterm Human Milk

Author	Year	Reference	Country	Study Design	Estimate [95% CI]
Arsic, A	2012	84	Serbia	Longitudinal	6.46 [4.52, 8.40]
Aydin, I	2014	85	Turkey	Longitudinal	4.60 [2.99, 6.21]
Berenhauser, A	2012	86	Brazil	Longitudinal	2.25 [−1.73, 6.23]
Bitman, J	1983	87	USA	Longitudinal	7.04 [5.61, 8.47]
Bobinski, R	2013	88	Poland	Longitudinal	3.82 [1.58, 6.07]
Castillo, F	2021	89	Spain	Longitudinal	4.74 [3.09, 6.39]
de Oliveira, S	2017	50	France	RCT	6.50 [5.99, 7.01]
Ehrenkranz, R	1984	51	USA	Longitudinal	1.60 [−1.27, 4.48]
Genzel−Boroviczeny, O	1997	91	Germany	Longitudinal	5.28 [3.37, 7.20]
Hossain, Z	2016	92	Canada	Longitudinal	5.54 [4.66, 6.42]
Innis, S	1990	93	Canada	Longitudinal	6.00 [3.90, 8.10]
Jang, S	2011	94	Korea	Longitudinal	5.09 [1.39, 8.78]
Kovacs, A	2005	95	Hungary	Longitudinal	5.37 [2.64, 8.10]
Lepage, G	1984	96	Canada	Cross sectional	5.84 [4.31, 7.36]
Maas, C	2017	98	Germany	Cross Sectional	7.40 [6.96, 7.83]
Marc, I	2011	99	Canada	Longitudinal	7.50 [6.12, 8.88]
Moltó−Puigmartí, C	2011	36	Spain	Cross Sectional	3.03 [0.58, 5.47]
Nilsson, A	2018	100	Sweden	Cross Sectional	3.64 [3.36, 3.92]
Sabel, K	2009	101	Sweden	Cross sectional	3.58 [3.06, 4.10]
Silber, G	1988	80	USA	Longitudinal	5.52 [2.86, 8.18]
RE Model (Q = 304.19, df = 19, p = 0.000; I^2 = 89.4%)					5.24 [4.53, 5.95]

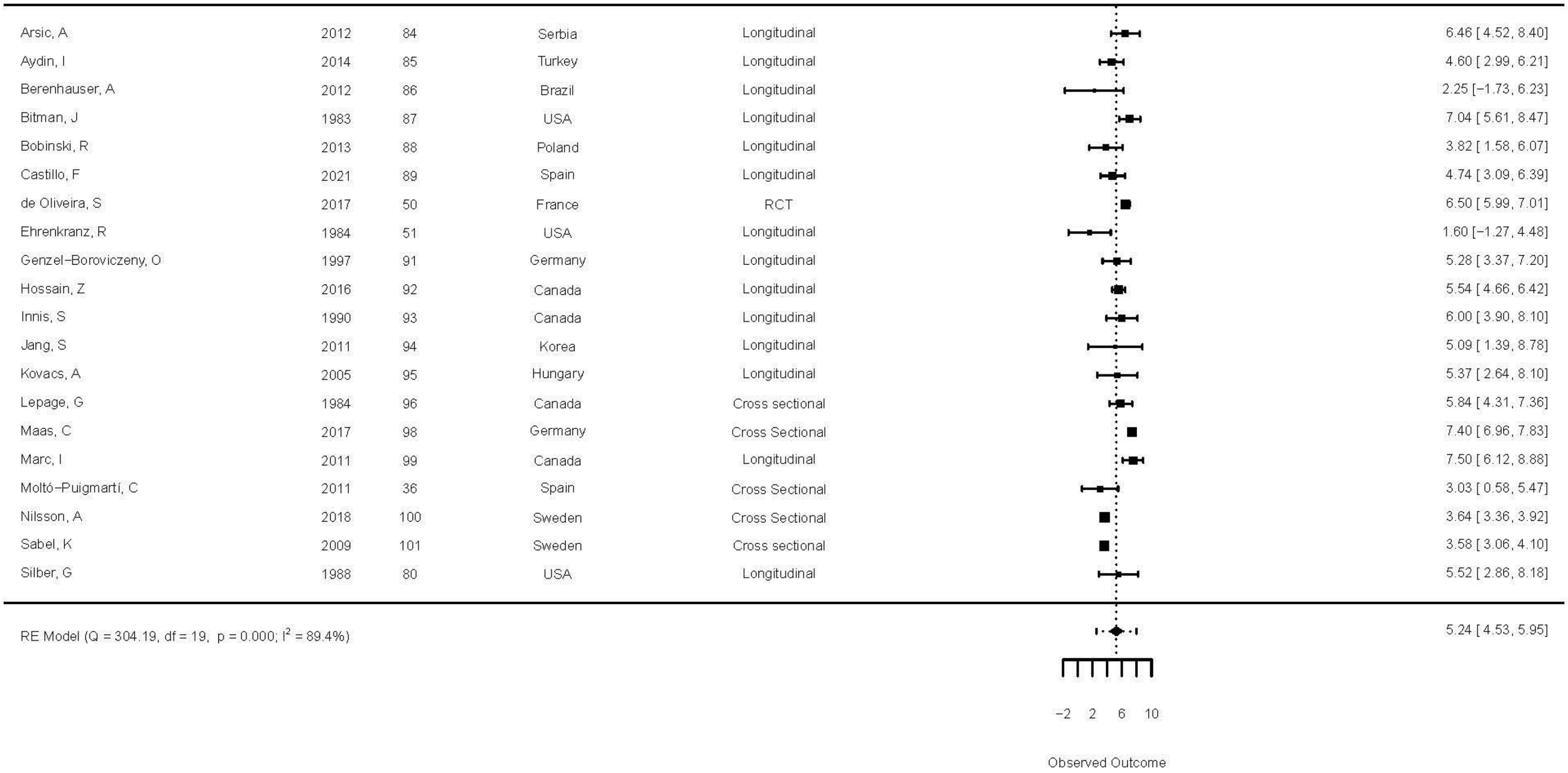

14:0

:14:0 concentration (w/w %) in Preterm Human Milk

Author	Year	Reference	Country	Study Design	Estimate [95% CI]
Arsic, A	2012	84	Serbia	Longitudinal	6.81 [5.07, 8.55]
Aydin, I	2014	85	Turkey	Longitudinal	9.33 [7.77, 10.88]
Berenhauser, A	2012	86	Brazil	Longitudinal	5.40 [1.73, 9.08]
Bitman, J	1983	87	USA	Longitudinal	8.01 [6.50, 9.52]
Bobinski, R	2013	88	Poland	Longitudinal	7.14 [6.48, 7.80]
Castillo, F	2021	89	Spain	Longitudinal	6.15 [4.83, 7.48]
de Oliveira, S	2017	50	France	RCT	7.60 [7.09, 8.11]
Ehrenkranz, R	1984	51	USA	Longitudinal	6.17 [3.34, 8.99]
Fares, S	2018	90	Tunisia	Cross sectional	6.66 [6.06, 7.25]
Genzel–Boroviczeny, O	1997	91	Germany	Longitudinal	8.38 [7.28, 9.49]
Hossain, Z	2016	92	Canada	Longitudinal	5.90 [4.96, 6.84]
Innis, S	1990	93	Canada	Longitudinal	6.70 [4.74, 8.66]
Jang, S	2011	94	Korea	Longitudinal	8.47 [7.41, 9.53]
Kovacs, A	2005	95	Hungary	Longitudinal	6.65 [3.50, 9.80]
Lepage, G	1984	96	Canada	Cross sectional	6.68 [4.97, 8.39]
Luukkainen, P	1994	97	Finland	Longitudinal	8.15 [6.65, 9.65]
Maas, C	2017	98	Germany	Cross Sectional	8.17 [7.88, 8.46]
Marc, I	2011	99	Canada	Longitudinal	9.40 [7.64, 11.16]
Moltó–Puigmartí, C	2011	36	Spain	Cross Sectional	4.52 [2.82, 6.23]
Nilsson, A	2018	100	Sweden	Cross Sectional	8.04 [7.05, 9.04]
Sabel, K	2009	101	Sweden	Cross sectional	7.12 [6.41, 7.84]
Silber, G	1988	80	USA	Longitudinal	5.32 [2.14, 8.49]
RE Model ($Q = 79.87$, $df = 21$, $p = 0.000$; $I^2 = 78.2\%$)					7.31 [6.83, 7.79]

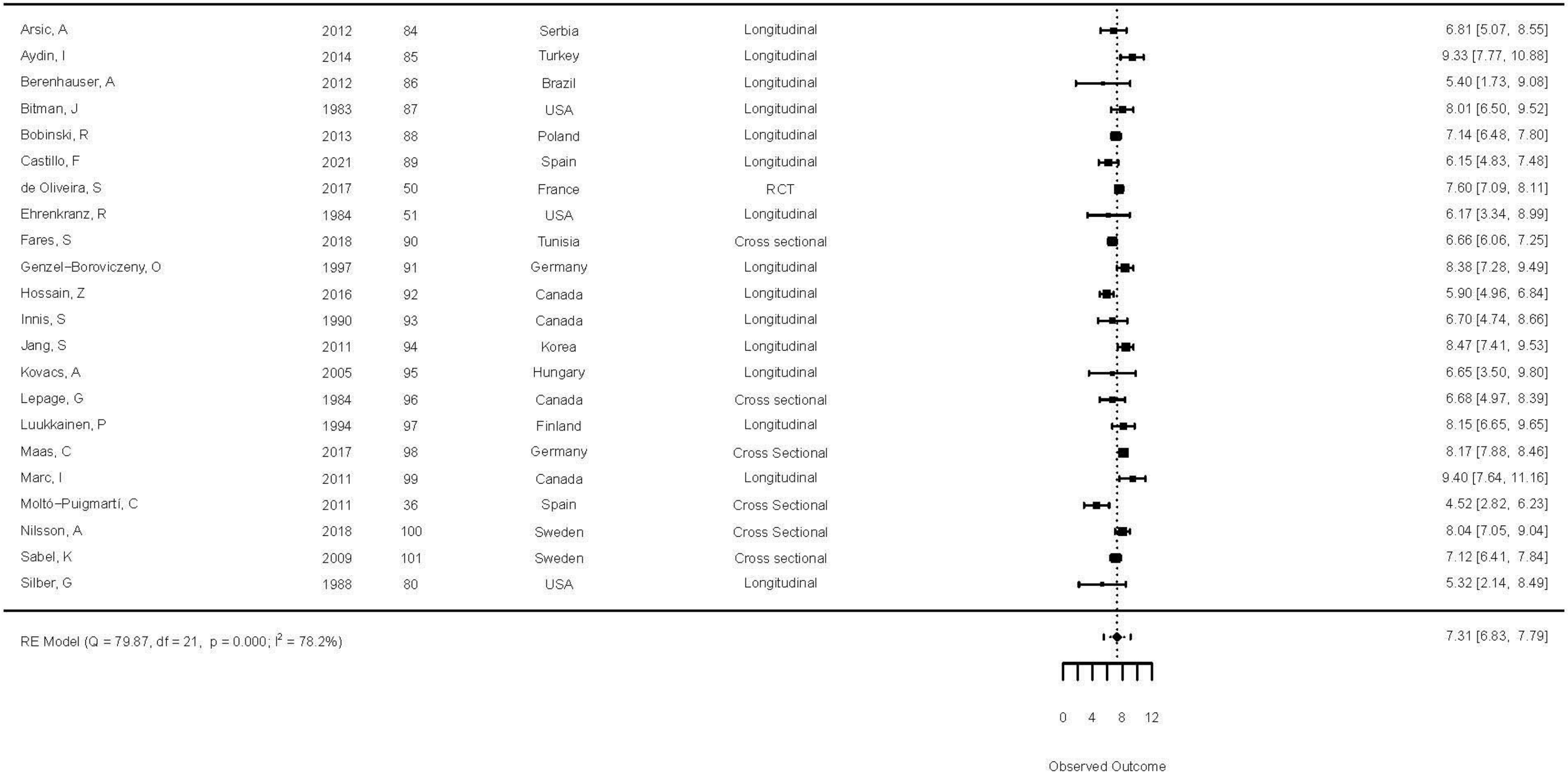

14:1

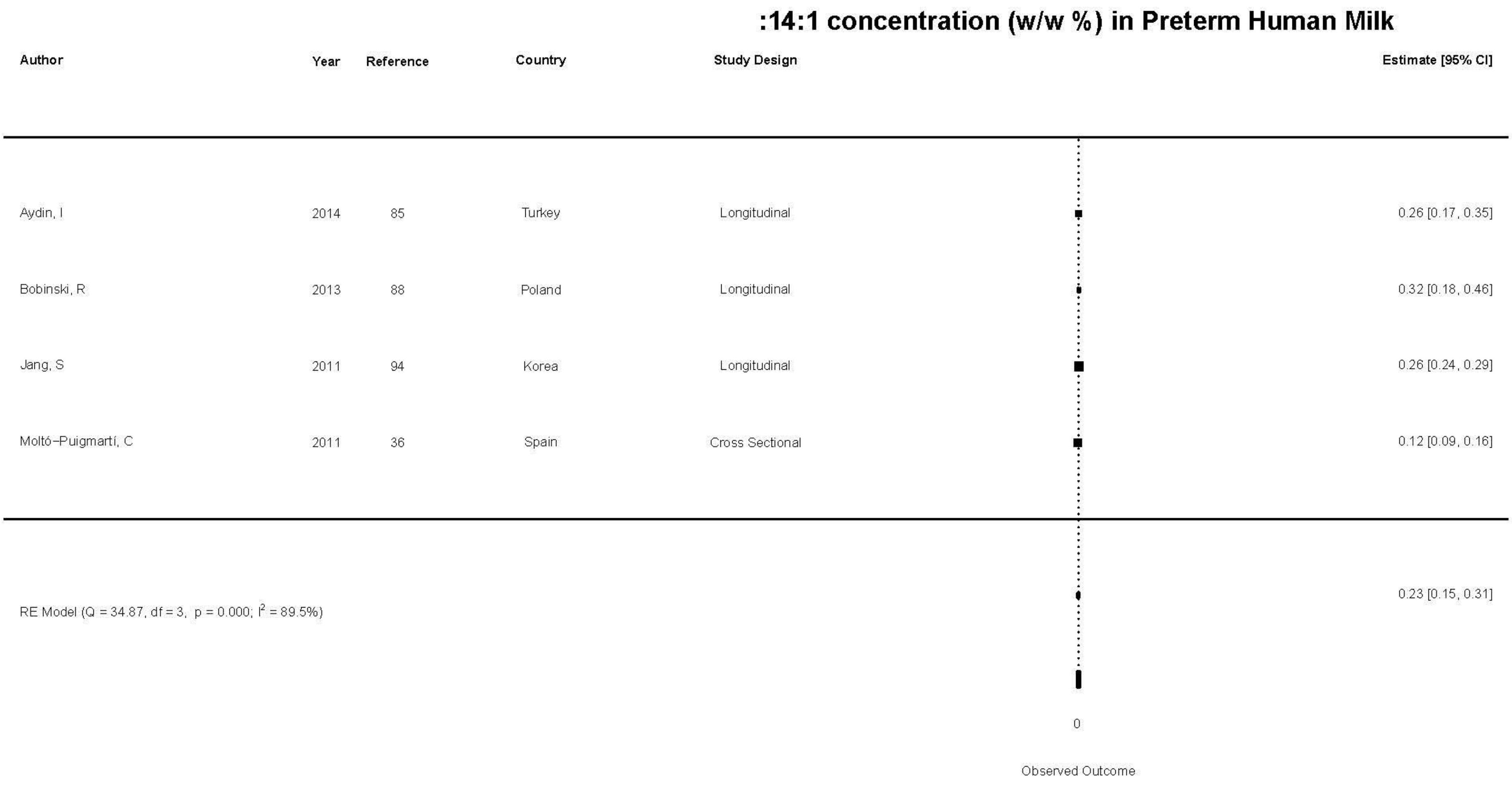
:14:1 concentration (w/w %) in Preterm Human Milk
Author
Year
Reference
Country
Study Design
Estimate [95% CI]
Aydin, I
2014
85
Turkey
Longitudinal
0.26 [0.17, 0.35]
Bobinski, R
2013
88
Poland
Longitudinal
0.32 [0.18, 0.46]
Jang, S
2011
94
Korea
Longitudinal
0.26 [0.24, 0.29]
Moltó−Puigmartí, C
2011
36
Spain
Cross Sectional
0.12 [0.09, 0.16]
RE Model (Q = 34.87, df = 3, p = 0.000; I² = 89.5%)
0.23 [0.15, 0.31]
0
Observed Outcome

Corresponding Author, Ariana Bailey

14:1-n5

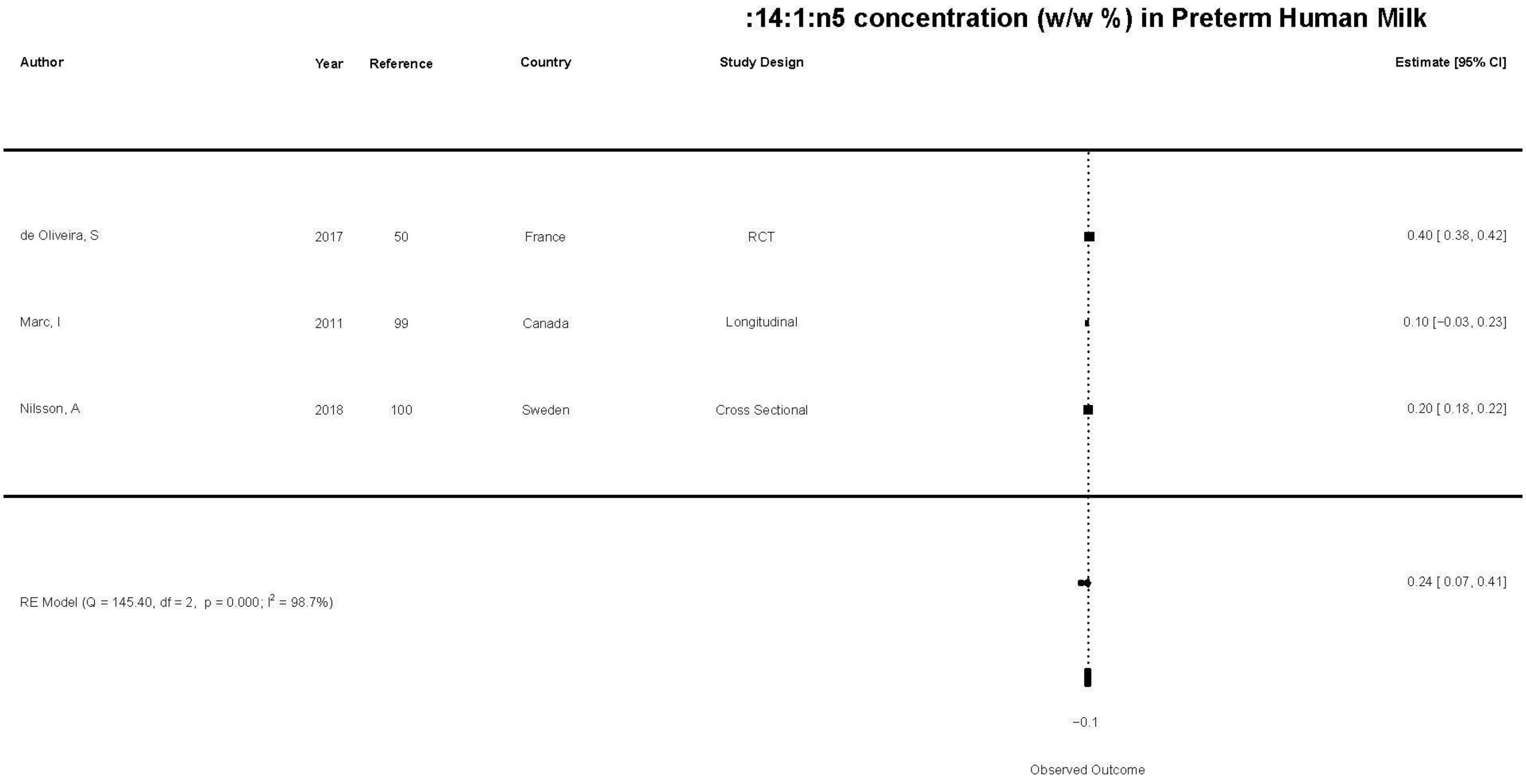

15:0

:15:0 concentration (w/w %) in Preterm Human Milk

Author	Year	Reference	Country	Study Design	Estimate [95% CI]
Arsic, A	2012	84	Serbia	Longitudinal	0.21 [−0.18, 0.60]
Aydin, I	2014	85	Turkey	Longitudinal	0.55 [0.41, 0.69]
Berenhauser, A	2012	86	Brazil	Longitudinal	0.12 [0.05, 0.20]
Bitman, J	1983	87	USA	Longitudinal	0.25 [0.17, 0.32]
Bobinski, R	2013	88	Poland	Longitudinal	0.31 [0.23, 0.38]
Moltó−Puigmartí, C	2011	36	Spain	Cross Sectional	0.20 [0.17, 0.23]
Nilsson, A	2018	100	Sweden	Cross Sectional	0.27 [0.26, 0.28]
RE Model (Q = 47.36, df = 6, p = 0.000; I^2 = 96.1%)					0.27 [0.17, 0.37]

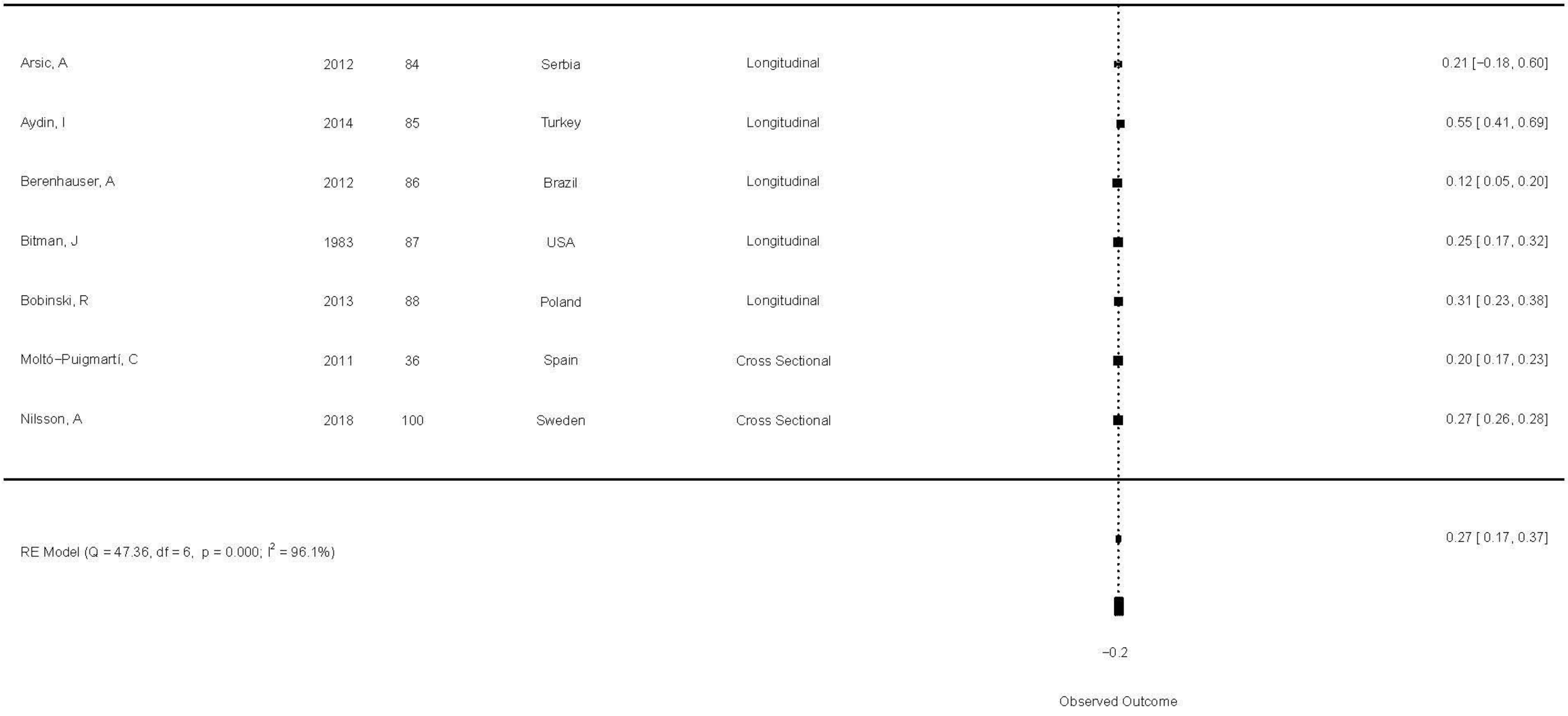

16:0

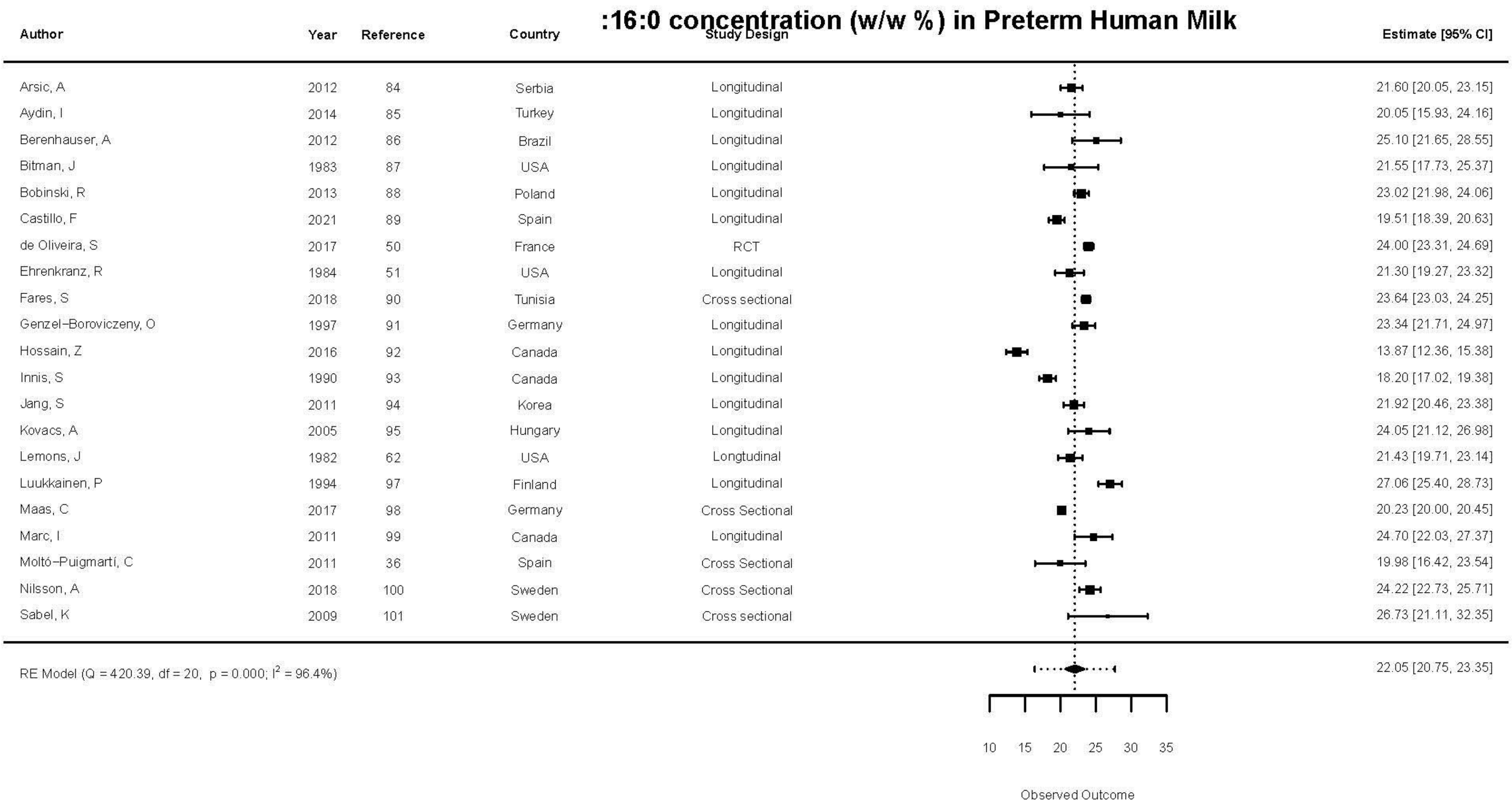
:16:0 concentration (w/w %) in Preterm Human Milk
Author Year Reference Country Study Design Estimate [95% CI]
Arsic, A 2012 84 Serbia Longitudinal 21.60 [20.05, 23.15]
Aydin, I 2014 85 Turkey Longitudinal 20.05 [15.93, 24.16]
Berenhauser, A 2012 86 Brazil Longitudinal 25.10 [21.65, 28.55]
Bitman, J 1983 87 USA Longitudinal 21.55 [17.73, 25.37]
Bobinski, R 2013 88 Poland Longitudinal 23.02 [21.98, 24.06]
Castillo, F 2021 89 Spain Longitudinal 19.51 [18.39, 20.63]
de Oliveira, S 2017 50 France RCT 24.00 [23.31, 24.69]
Ehrenkranz, R 1984 51 USA Longitudinal 21.30 [19.27, 23.32]
Fares, S 2018 90 Tunisia Cross sectional 23.64 [23.03, 24.25]
Genzel−Boroviczeny, O 1997 91 Germany Longitudinal 23.34 [21.71, 24.97]
Hossain, Z 2016 92 Canada Longitudinal 13.87 [12.36, 15.38]
Innis, S 1990 93 Canada Longitudinal 18.20 [17.02, 19.38]
Jang, S 2011 94 Korea Longitudinal 21.92 [20.46, 23.38]
Kovacs, A 2005 95 Hungary Longitudinal 24.05 [21.12, 26.98]
Lemons, J 1982 62 USA Longtudinal 21.43 [19.71, 23.14]
Luukkainen, P 1994 97 Finland Longitudinal 27.06 [25.40, 28.73]
Maas, C 2017 98 Germany Cross Sectional 20.23 [20.00, 20.45]
Marc, I 2011 99 Canada Longitudinal 24.70 [22.03, 27.37]
Moltó−Puigmartí, C 2011 36 Spain Cross Sectional 19.98 [16.42, 23.54]
Nilsson, A 2018 100 Sweden Cross Sectional 24.22 [22.73, 25.71]
Sabel, K 2009 101 Sweden Cross sectional 26.73 [21.11, 32.35]
RE Model (Q = 420.39, df = 20, p = 0.000; I² = 96.4%) 22.05 [20.75, 23.35]
10 15 20 25 30 35
Observed Outcome

16:1

:16:1 concentration (w/w %) in Preterm Human Milk

Author	Year	Reference	Country	Study Design	Estimate [95% CI]
Arsic, A	2012	84	Serbia	Longitudinal	1.72 [1.28, 2.17]
Aydin, I	2014	85	Turkey	Longitudinal	2.16 [1.50, 2.82]
Berenhauser, A	2012	86	Brazil	Longitudinal	0.54 [0.49, 0.59]
Bitman, J	1983	87	USA	Longitudinal	2.73 [2.21, 3.24]
Bobinski, R	2013	88	Poland	Longitudinal	2.92 [2.53, 3.32]
Ehrenkranz, R	1984	51	USA	Longitudinal	4.12 [3.22, 5.02]
Fares, S	2018	90	Tunisia	Cross sectional	3.40 [3.11, 3.68]
Jang, S	2011	94	Korea	Longitudinal	2.76 [2.54, 2.99]
Luukkainen, P	1994	97	Finland	Longitudinal	3.48 [3.25, 3.70]
Sabel, K	2009	101	Sweden	Cross sectional	2.65 [2.50, 2.81]
RE Model (Q = 1846.49, df = 9, p = 0.000; I^2 = 98.7%)					2.63 [2.01, 3.25]

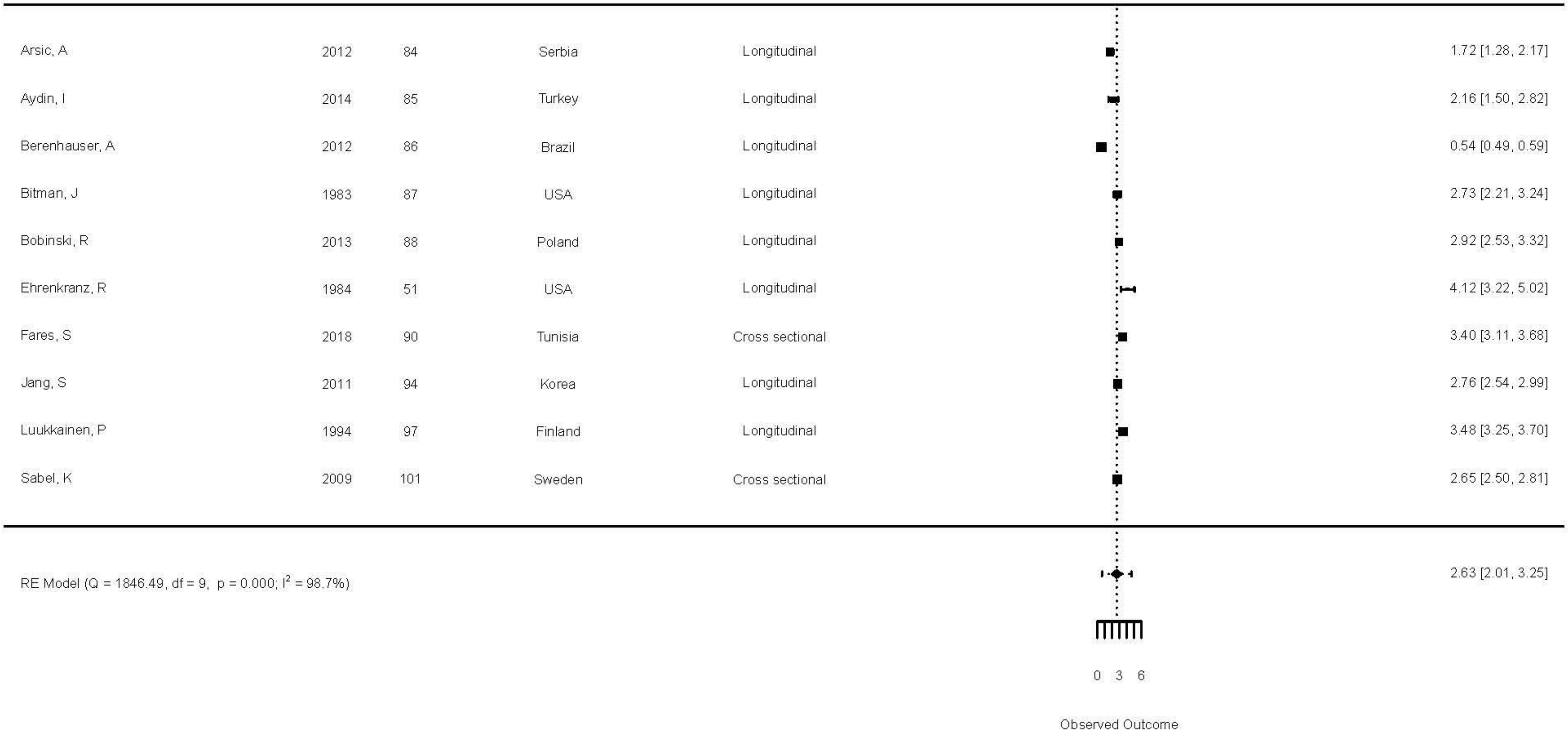

16:1n-7

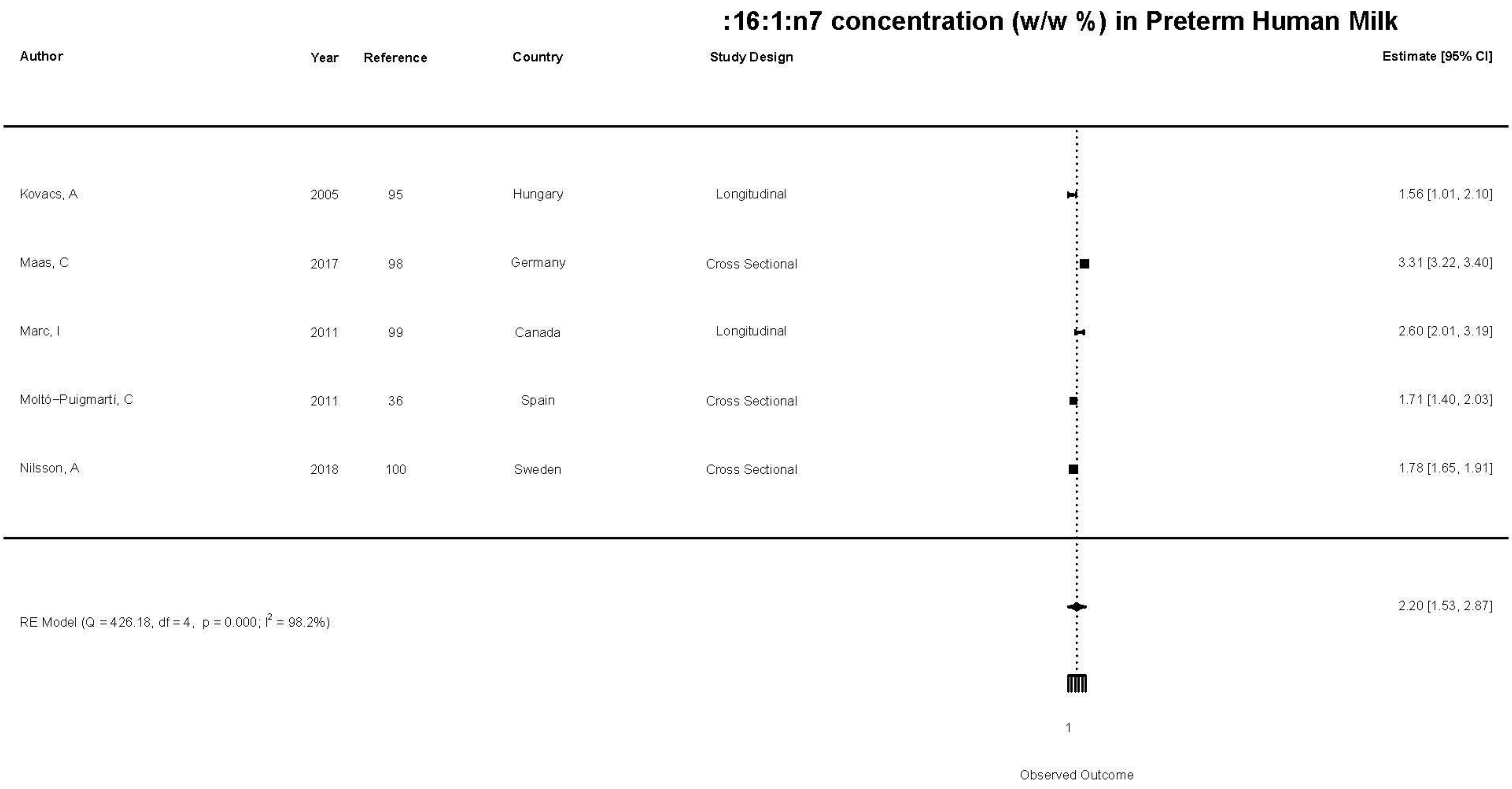
:16:1:n7 concentration (w/w %) in Preterm Human Milk
Author
Year
Reference
Country
Study Design
Estimate [95% CI]
Kovacs, A 2005 95 Hungary Longitudinal 1.56 [1.01, 2.10]
Maas, C 2017 98 Germany Cross Sectional 3.31 [3.22, 3.40]
Marc, I 2011 99 Canada Longitudinal 2.60 [2.01, 3.19]
Moltó−Puigmartí, C 2011 36 Spain Cross Sectional 1.71 [1.40, 2.03]
Nilsson, A 2018 100 Sweden Cross Sectional 1.78 [1.65, 1.91]
RE Model (Q = 426.18, df = 4, p = 0.000; I^2 = 98.2%) 2.20 [1.53, 2.87]
1
Observed Outcome

16:1n-9

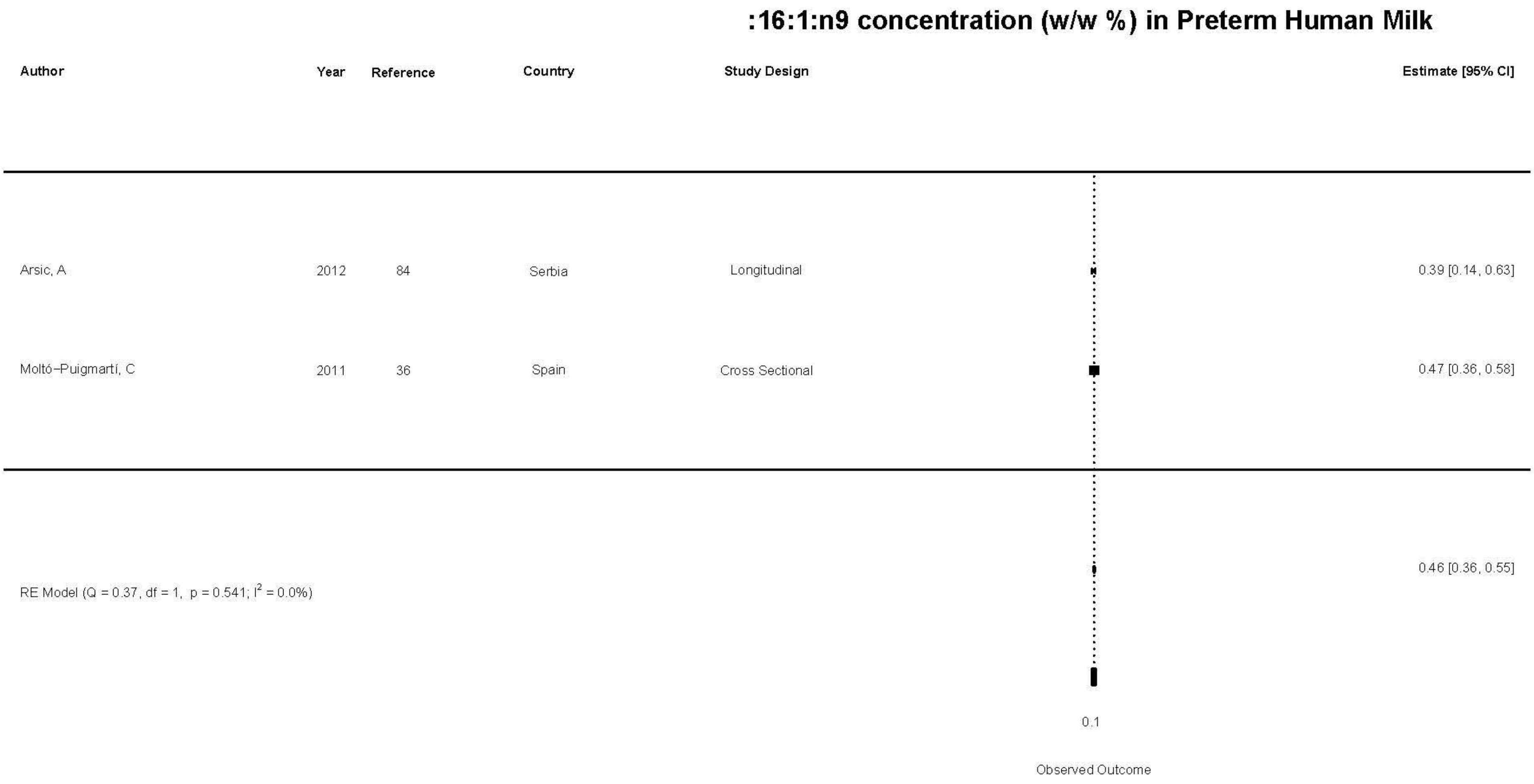
:16:1:n9 concentration (w/w %) in Preterm Human Milk
Author
Year
Reference
Country
Study Design
Estimate [95% CI]
Arsic, A
2012
84
Serbia
Longitudinal
0.39 [0.14, 0.63]
Moltó−Puigmartí, C
2011
36
Spain
Cross Sectional
0.47 [0.36, 0.58]
RE Model (Q = 0.37, df = 1, p = 0.541; I² = 0.0%)
0.46 [0.36, 0.55]
0.1
Observed Outcome

17:0

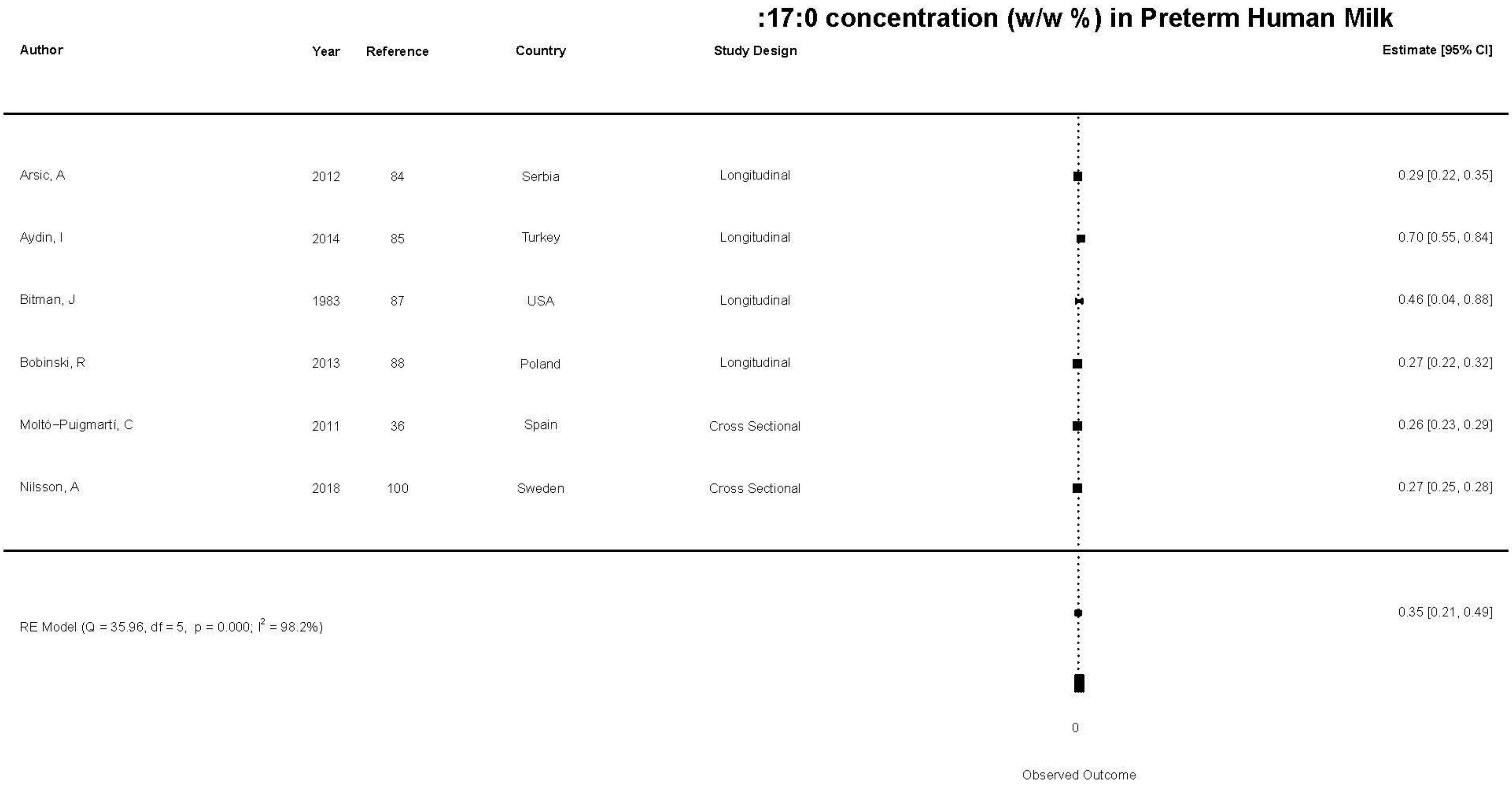
:17:0 concentration (w/w %) in Preterm Human Milk
Author
Year
Reference
Country
Study Design
Estimate [95% CI]
Arsic, A 2012 84 Serbia Longitudinal 0.29 [0.22, 0.35]
Aydin, I 2014 85 Turkey Longitudinal 0.70 [0.55, 0.84]
Bitman, J 1983 87 USA Longitudinal 0.46 [0.04, 0.88]
Bobinski, R 2013 88 Poland Longitudinal 0.27 [0.22, 0.32]
Moltó–Puigmartí, C 2011 36 Spain Cross Sectional 0.26 [0.23, 0.29]
Nilsson, A 2018 100 Sweden Cross Sectional 0.27 [0.25, 0.28]
RE Model (Q = 35.96, df = 5, p = 0.000; I² = 98.2%) 0.35 [0.21, 0.49]
0
Observed Outcome

17:1

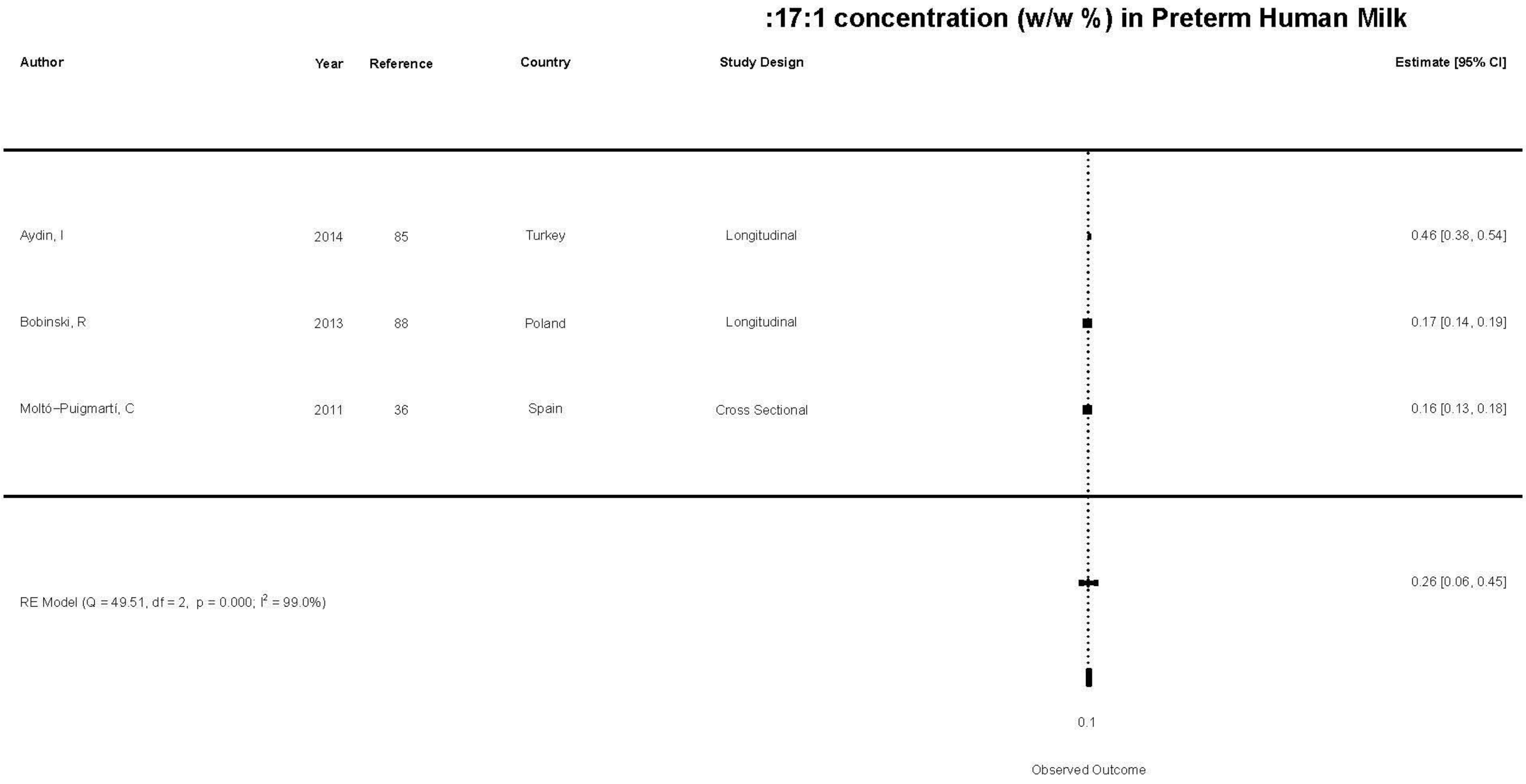
:17:1 concentration (w/w %) in Preterm Human Milk
Author
Year
Reference
Country
Study Design
Estimate [95% CI]
Aydin, I
2014
85
Turkey
Longitudinal
0.46 [0.38, 0.54]
Bobinski, R
2013
88
Poland
Longitudinal
0.17 [0.14, 0.19]
Moltó–Puigmartí, C
2011
36
Spain
Cross Sectional
0.16 [0.13, 0.18]
RE Model (Q = 49.51, df = 2, p = 0.000; I² = 99.0%)
0.26 [0.06, 0.45]
0.1
Observed Outcome

18:0

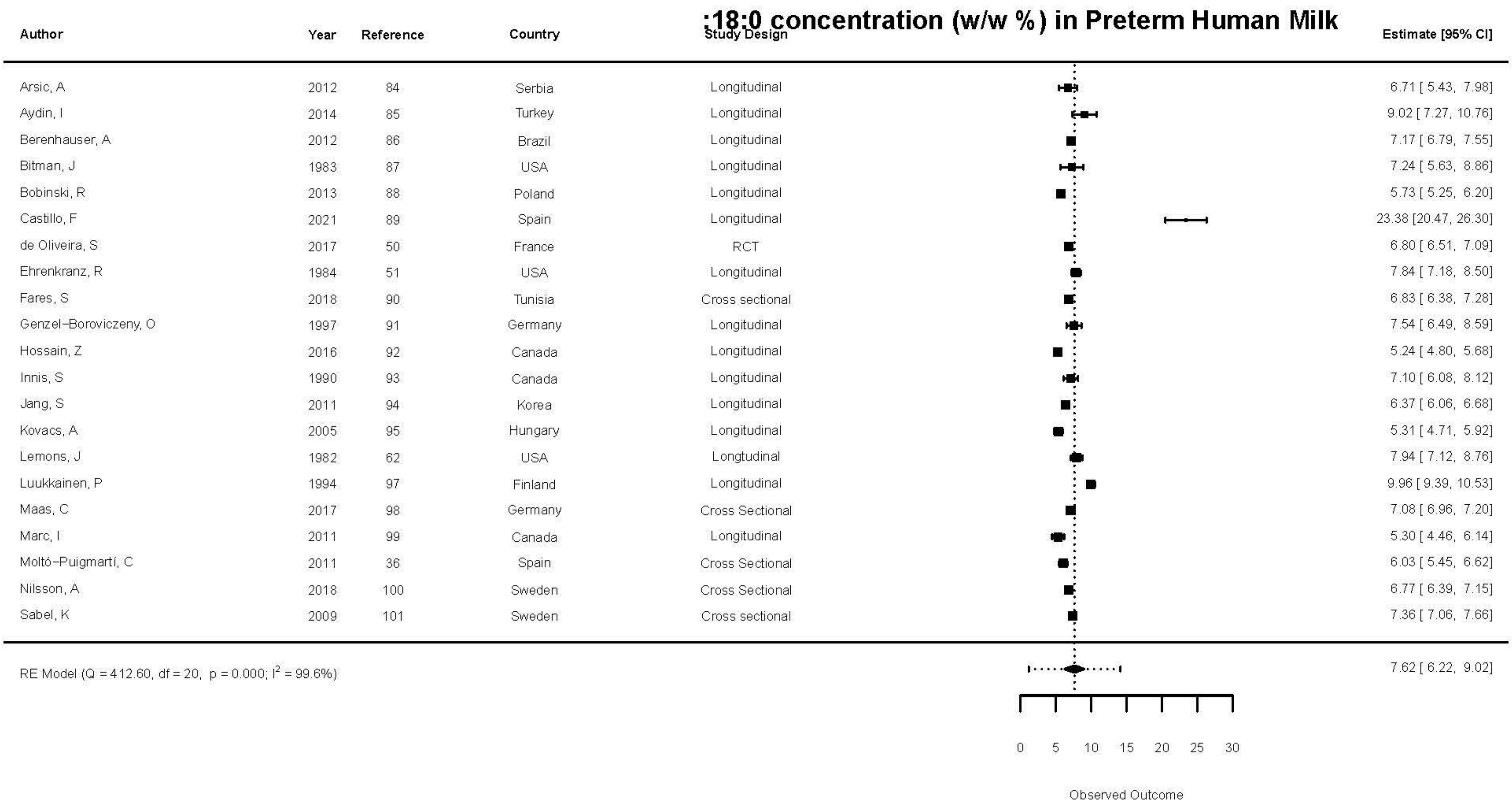
:18:0 concentration (w/w %) in Preterm Human Milk
Author | Year | Reference | Country | Study Design | Estimate [95% CI]
Arsic, A | 2012 | 84 | Serbia | Longitudinal | 6.71 [5.43, 7.98]
Aydin, I | 2014 | 85 | Turkey | Longitudinal | 9.02 [7.27, 10.76]
Berenhauser, A | 2012 | 86 | Brazil | Longitudinal | 7.17 [6.79, 7.55]
Bitman, J | 1983 | 87 | USA | Longitudinal | 7.24 [5.63, 8.86]
Bobinski, R | 2013 | 88 | Poland | Longitudinal | 5.73 [5.25, 6.20]
Castillo, F | 2021 | 89 | Spain | Longitudinal | 23.38 [20.47, 26.30]
de Oliveira, S | 2017 | 50 | France | RCT | 6.80 [6.51, 7.09]
Ehrenkranz, R | 1984 | 51 | USA | Longitudinal | 7.84 [7.18, 8.50]
Fares, S | 2018 | 90 | Tunisia | Cross sectional | 6.83 [6.38, 7.28]
Genzel−Boroviczeny, O | 1997 | 91 | Germany | Longitudinal | 7.54 [6.49, 8.59]
Hossain, Z | 2016 | 92 | Canada | Longitudinal | 5.24 [4.80, 5.68]
Innis, S | 1990 | 93 | Canada | Longitudinal | 7.10 [6.08, 8.12]
Jang, S | 2011 | 94 | Korea | Longitudinal | 6.37 [6.06, 6.68]
Kovacs, A | 2005 | 95 | Hungary | Longitudinal | 5.31 [4.71, 5.92]
Lemons, J | 1982 | 62 | USA | Longtudinal | 7.94 [7.12, 8.76]
Luukkainen, P | 1994 | 97 | Finland | Longitudinal | 9.96 [9.39, 10.53]
Maas, C | 2017 | 98 | Germany | Cross Sectional | 7.08 [6.96, 7.20]
Marc, I | 2011 | 99 | Canada | Longitudinal | 5.30 [4.46, 6.14]
Moltó−Puigmartí, C | 2011 | 36 | Spain | Cross Sectional | 6.03 [5.45, 6.62]
Nilsson, A | 2018 | 100 | Sweden | Cross Sectional | 6.77 [6.39, 7.15]
Sabel, K | 2009 | 101 | Sweden | Cross sectional | 7.36 [7.06, 7.66]
RE Model (Q = 412.60, df = 20, p = 0.000; I² = 99.6%) | 7.62 [6.22, 9.02]
0 5 10 15 20 25 30
Observed Outcome

18:1

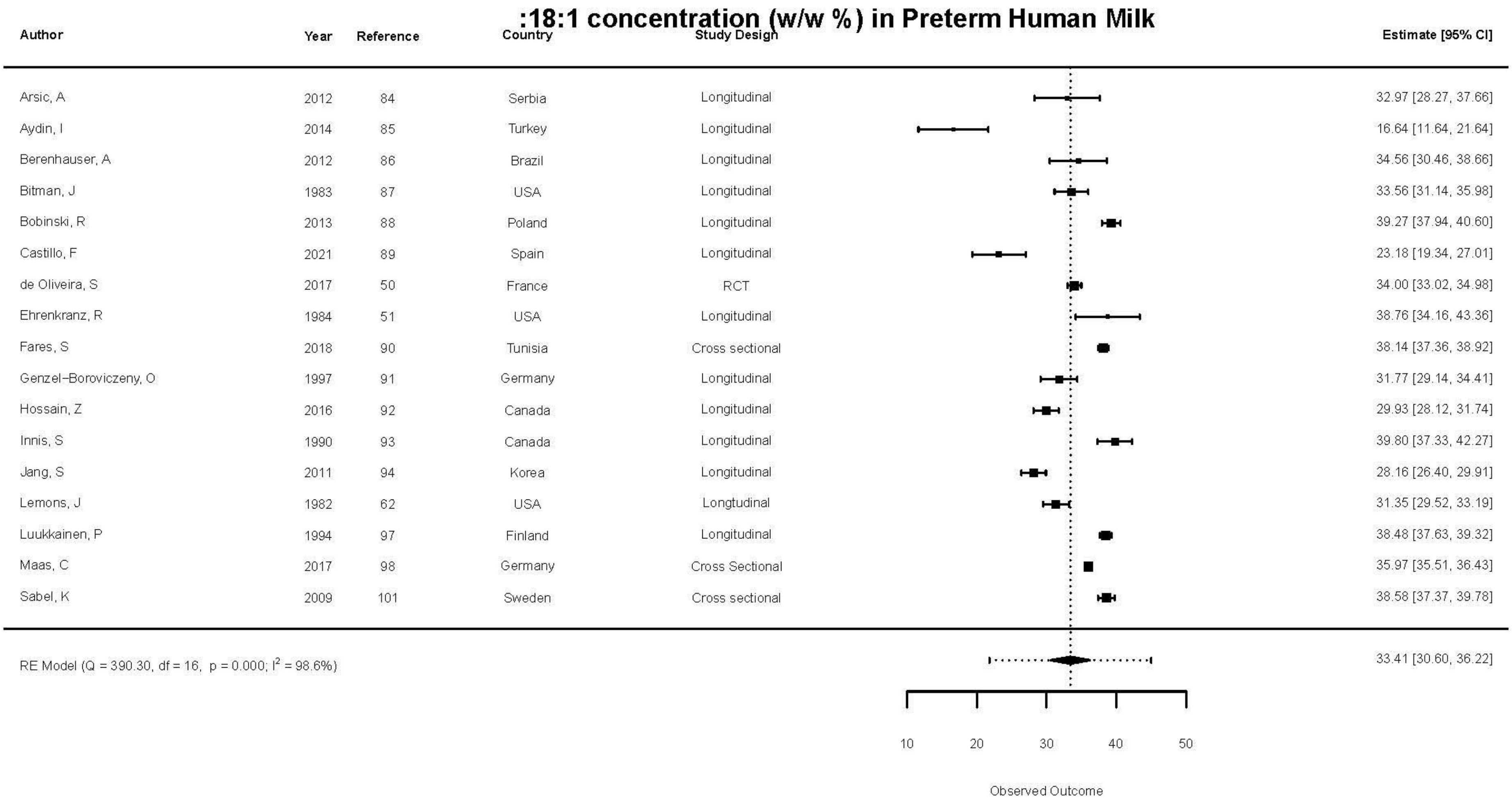
:18:1 concentration (w/w %) in Preterm Human Milk
Author
Year
Reference
Country
Study Design
Estimate [95% CI]
Arsic, A 2012 84 Serbia Longitudinal 32.97 [28.27, 37.66]
Aydin, I 2014 85 Turkey Longitudinal 16.64 [11.64, 21.64]
Berenhauser, A 2012 86 Brazil Longitudinal 34.56 [30.46, 38.66]
Bitman, J 1983 87 USA Longitudinal 33.56 [31.14, 35.98]
Bobinski, R 2013 88 Poland Longitudinal 39.27 [37.94, 40.60]
Castillo, F 2021 89 Spain Longitudinal 23.18 [19.34, 27.01]
de Oliveira, S 2017 50 France RCT 34.00 [33.02, 34.98]
Ehrenkranz, R 1984 51 USA Longitudinal 38.76 [34.16, 43.36]
Fares, S 2018 90 Tunisia Cross sectional 38.14 [37.36, 38.92]
Genzel−Boroviczeny, O 1997 91 Germany Longitudinal 31.77 [29.14, 34.41]
Hossain, Z 2016 92 Canada Longitudinal 29.93 [28.12, 31.74]
Innis, S 1990 93 Canada Longitudinal 39.80 [37.33, 42.27]
Jang, S 2011 94 Korea Longitudinal 28.16 [26.40, 29.91]
Lemons, J 1982 62 USA Longtudinal 31.35 [29.52, 33.19]
Luukkainen, P 1994 97 Finland Longitudinal 38.48 [37.63, 39.32]
Maas, C 2017 98 Germany Cross Sectional 35.97 [35.51, 36.43]
Sabel, K 2009 101 Sweden Cross sectional 38.58 [37.37, 39.78]
RE Model (Q = 390.30, df = 16, p = 0.000; I² = 98.6%) 33.41 [30.60, 36.22]
10
20
30
40
50
Observed Outcome

18:1n-7

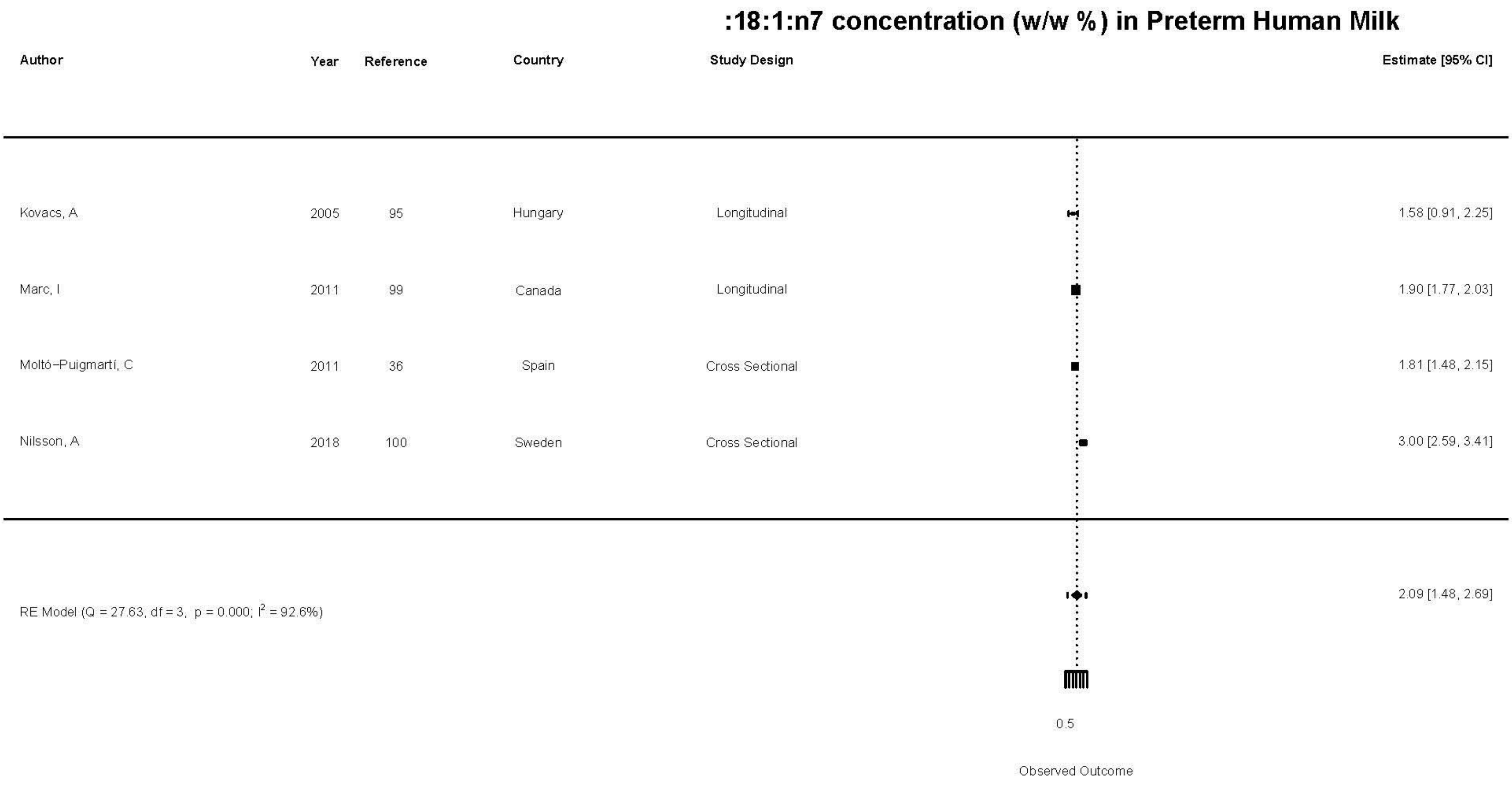
:18:1:n7 concentration (w/w %) in Preterm Human Milk
Author
Year
Reference
Country
Study Design
Estimate [95% CI]
Kovacs, A
2005
95
Hungary
Longitudinal
1.58 [0.91, 2.25]
Marc, I
2011
99
Canada
Longitudinal
1.90 [1.77, 2.03]
Moltó−Puigmartí, C
2011
36
Spain
Cross Sectional
1.81 [1.48, 2.15]
Nilsson, A
2018
100
Sweden
Cross Sectional
3.00 [2.59, 3.41]
RE Model (Q = 27.63, df = 3, p = 0.000; I² = 92.6%)
2.09 [1.48, 2.69]
0.5
Observed Outcome

18:1n-9

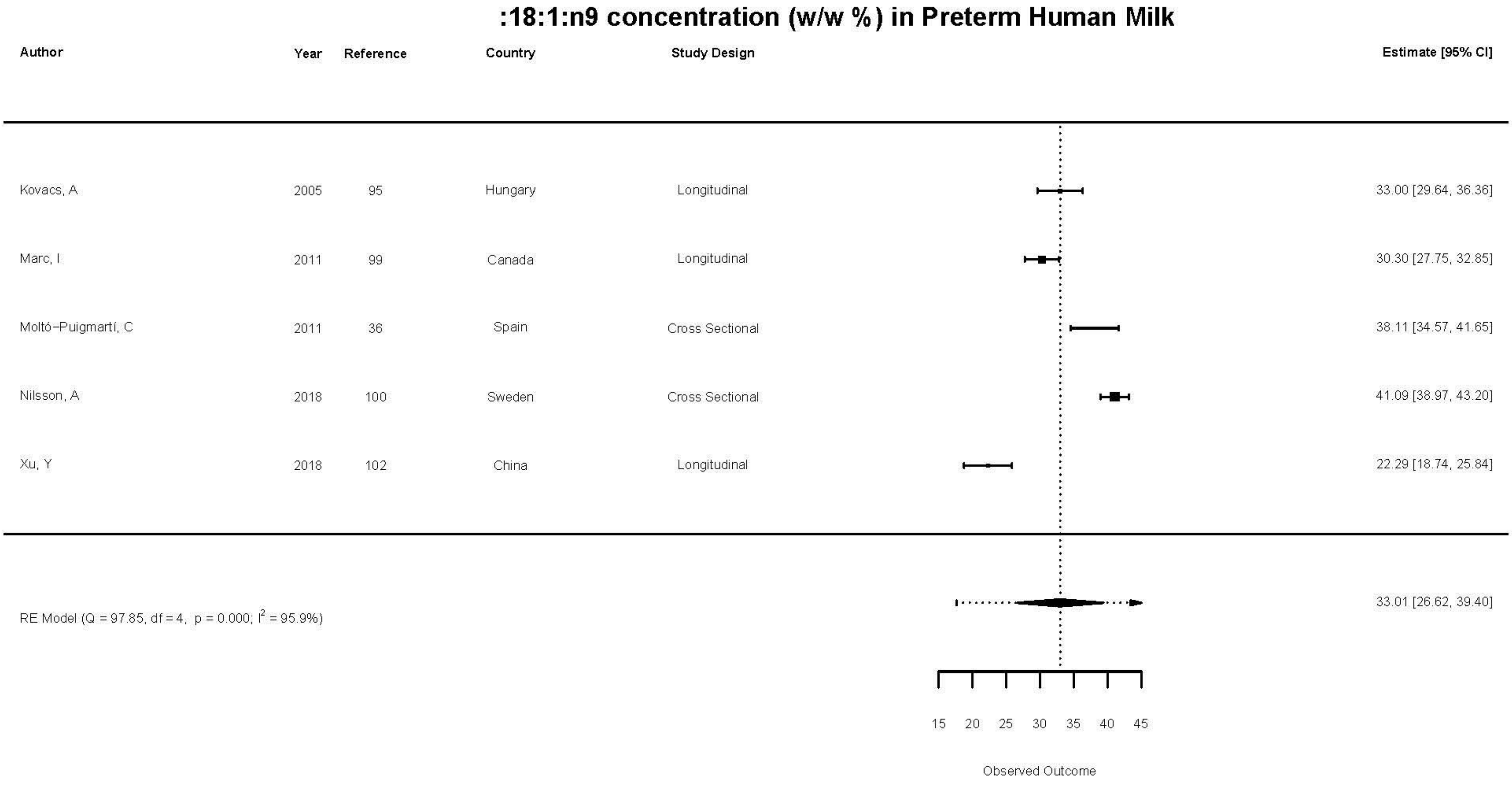
:18:1:n9 concentration (w/w %) in Preterm Human Milk
Author
Year
Reference
Country
Study Design
Estimate [95% CI]
Kovacs, A
2005
95
Hungary
Longitudinal
33.00 [29.64, 36.36]
Marc, I
2011
99
Canada
Longitudinal
30.30 [27.75, 32.85]
Moltó−Puigmartí, C
2011
36
Spain
Cross Sectional
38.11 [34.57, 41.65]
Nilsson, A
2018
100
Sweden
Cross Sectional
41.09 [38.97, 43.20]
Xu, Y
2018
102
China
Longitudinal
22.29 [18.74, 25.84]
RE Model (Q = 97.85, df = 4, p = 0.000; I² = 95.9%)
33.01 [26.62, 39.40]
15 20 25 30 35 40 45
Observed Outcome

18:2n-6

:18:2:n6 concentration (w/w %) in Preterm Human Milk

Author	Year	Reference	Country	Study Design	Estimate [95% CI]
Arsic, A	2012	84	Serbia	Longitudinal	15.74 [11.48, 20.01]
Aydin, I	2014	85	Turkey	Longitudinal	22.49 [18.37, 26.61]
Berenhauser, A	2012	86	Brazil	Longitudinal	20.30 [18.58, 22.03]
Bobinski, R	2013	88	Poland	Longitudinal	9.85 [8.31, 11.39]
Castillo, F	2021	89	Spain	Longitudinal	19.62 [18.89, 20.35]
de Oliveira, S	2017	50	France	RCT	9.40 [8.91, 9.89]
Ehrenkranz, R	1984	51	USA	Longitudinal	17.05 [15.11, 19.00]
Fares, S	2018	90	Tunisia	Cross sectional	15.54 [14.85, 16.22]
Genzel−Boroviczeny, O	1997	91	Germany	Longitudinal	9.95 [9.28, 10.63]
Hossain, Z	2016	92	Canada	Longitudinal	12.98 [11.28, 14.68]
Innis, S	1990	93	Canada	Longitudinal	11.10 [9.49, 12.71]
Jang, S	2011	94	Korea	Longitudinal	14.73 [13.43, 16.02]
Kovacs, A	2005	95	Hungary	Longitudinal	14.99 [10.50, 19.49]
Lemons, J	1982	62	USA	Longtudinal	11.22 [6.78, 15.66]
Luukkainen, P	1994	97	Finland	Longitudinal	9.73 [7.63, 11.84]
Maas, C	2017	98	Germany	Cross Sectional	12.77 [12.49, 13.05]
Marc, I	2011	99	Canada	Longitudinal	14.70 [11.98, 17.42]
Moltó−Puigmartí, C	2011	36	Spain	Cross Sectional	13.89 [12.28, 15.51]
Nilsson, A	2018	100	Sweden	Cross Sectional	9.84 [8.33, 11.35]
Sabel, K	2009	101	Sweden	Cross sectional	9.71 [9.15, 10.26]
Xu, Y	2018	102	China	Longitudinal	15.81 [13.41, 18.21]
RE Model (Q = 934.28, df = 20, p = 0.000; I^2 = 98.3%)					13.76 [12.14, 15.37]

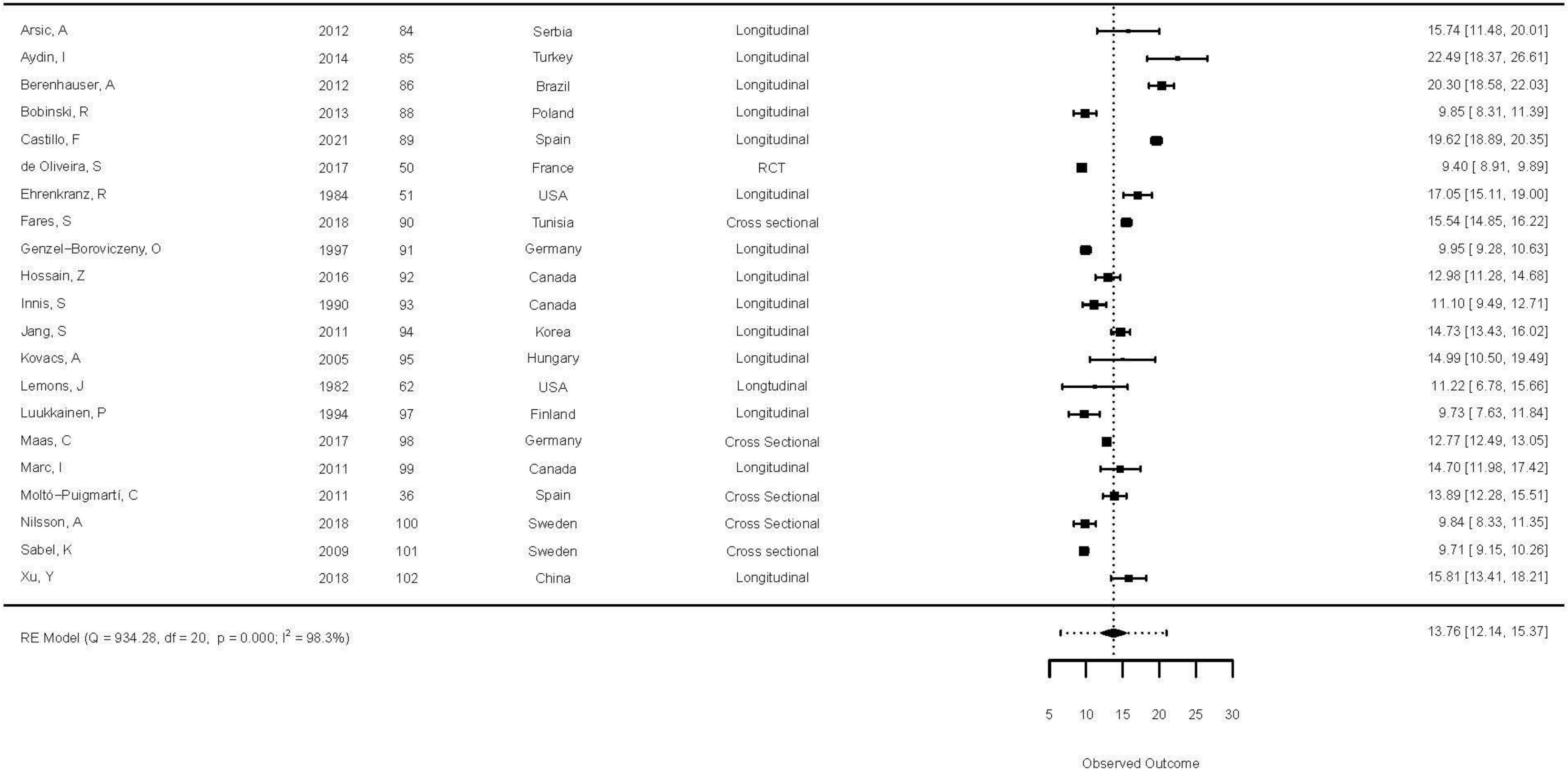

18:3n-3

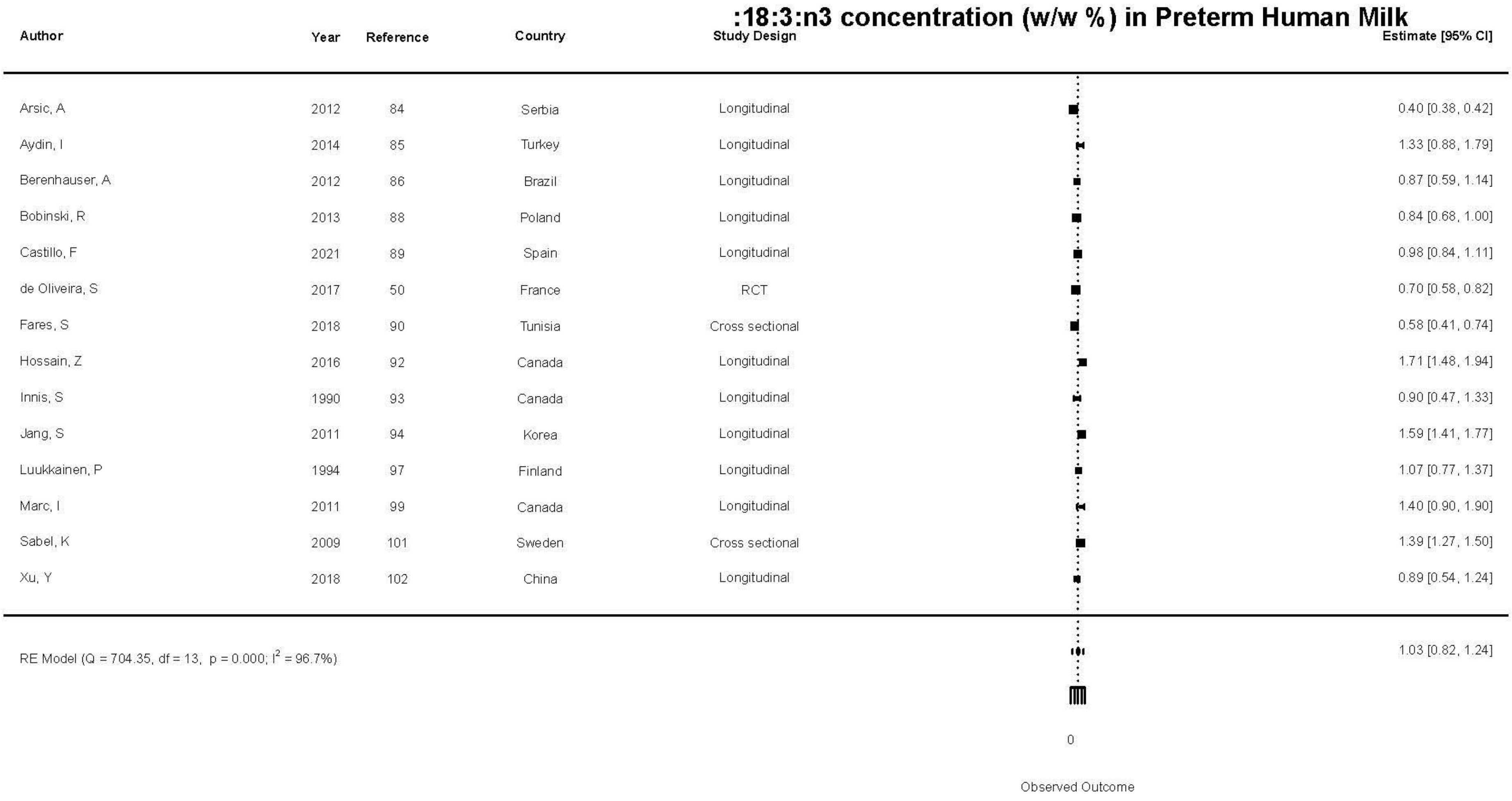
:18:3:n3 concentration (w/w %) in Preterm Human Milk
Author Year Reference Country Study Design Estimate [95% CI]
Arsic, A 2012 84 Serbia Longitudinal 0.40 [0.38, 0.42]
Aydin, I 2014 85 Turkey Longitudinal 1.33 [0.88, 1.79]
Berenhauser, A 2012 86 Brazil Longitudinal 0.87 [0.59, 1.14]
Bobinski, R 2013 88 Poland Longitudinal 0.84 [0.68, 1.00]
Castillo, F 2021 89 Spain Longitudinal 0.98 [0.84, 1.11]
de Oliveira, S 2017 50 France RCT 0.70 [0.58, 0.82]
Fares, S 2018 90 Tunisia Cross sectional 0.58 [0.41, 0.74]
Hossain, Z 2016 92 Canada Longitudinal 1.71 [1.48, 1.94]
Innis, S 1990 93 Canada Longitudinal 0.90 [0.47, 1.33]
Jang, S 2011 94 Korea Longitudinal 1.59 [1.41, 1.77]
Luukkainen, P 1994 97 Finland Longitudinal 1.07 [0.77, 1.37]
Marc, I 2011 99 Canada Longitudinal 1.40 [0.90, 1.90]
Sabel, K 2009 101 Sweden Cross sectional 1.39 [1.27, 1.50]
Xu, Y 2018 102 China Longitudinal 0.89 [0.54, 1.24]
RE Model (Q = 704.35, df = 13, p = 0.000; I2 = 96.7%) 1.03 [0.82, 1.24]
0
Observed Outcome

18:3n-6

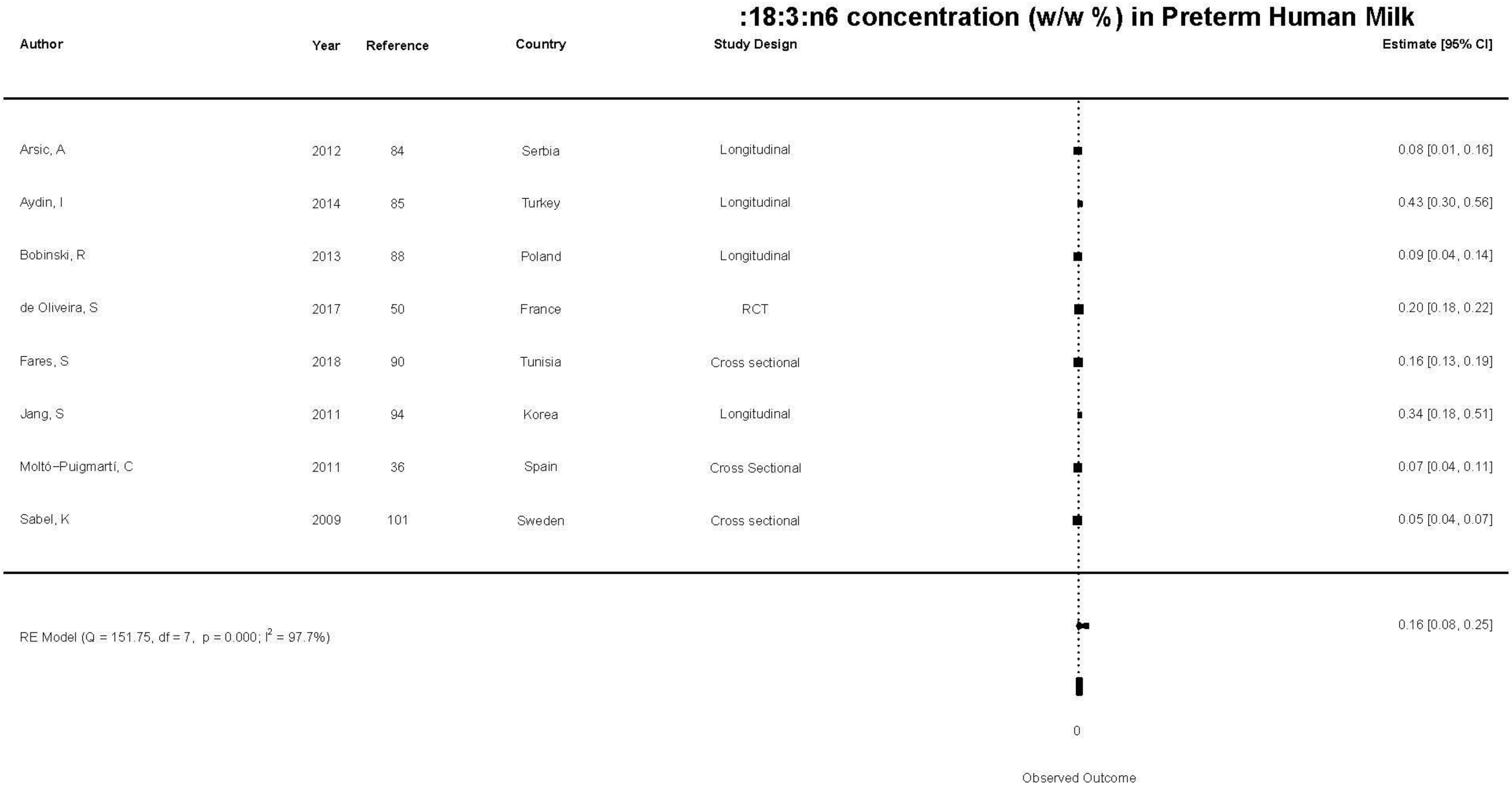
:18:3:n6 concentration (w/w %) in Preterm Human Milk
Author
Year
Reference
Country
Study Design
Estimate [95% CI]
Arsic, A 2012 84 Serbia Longitudinal 0.08 [0.01, 0.16]
Aydin, I 2014 85 Turkey Longitudinal 0.43 [0.30, 0.56]
Bobinski, R 2013 88 Poland Longitudinal 0.09 [0.04, 0.14]
de Oliveira, S 2017 50 France RCT 0.20 [0.18, 0.22]
Fares, S 2018 90 Tunisia Cross sectional 0.16 [0.13, 0.19]
Jang, S 2011 94 Korea Longitudinal 0.34 [0.18, 0.51]
Moltó−Puigmartí, C 2011 36 Spain Cross Sectional 0.07 [0.04, 0.11]
Sabel, K 2009 101 Sweden Cross sectional 0.05 [0.04, 0.07]
RE Model (Q = 151.75, df = 7, p = 0.000; I2 = 97.7%) 0.16 [0.08, 0.25]
0
Observed Outcome

20:0

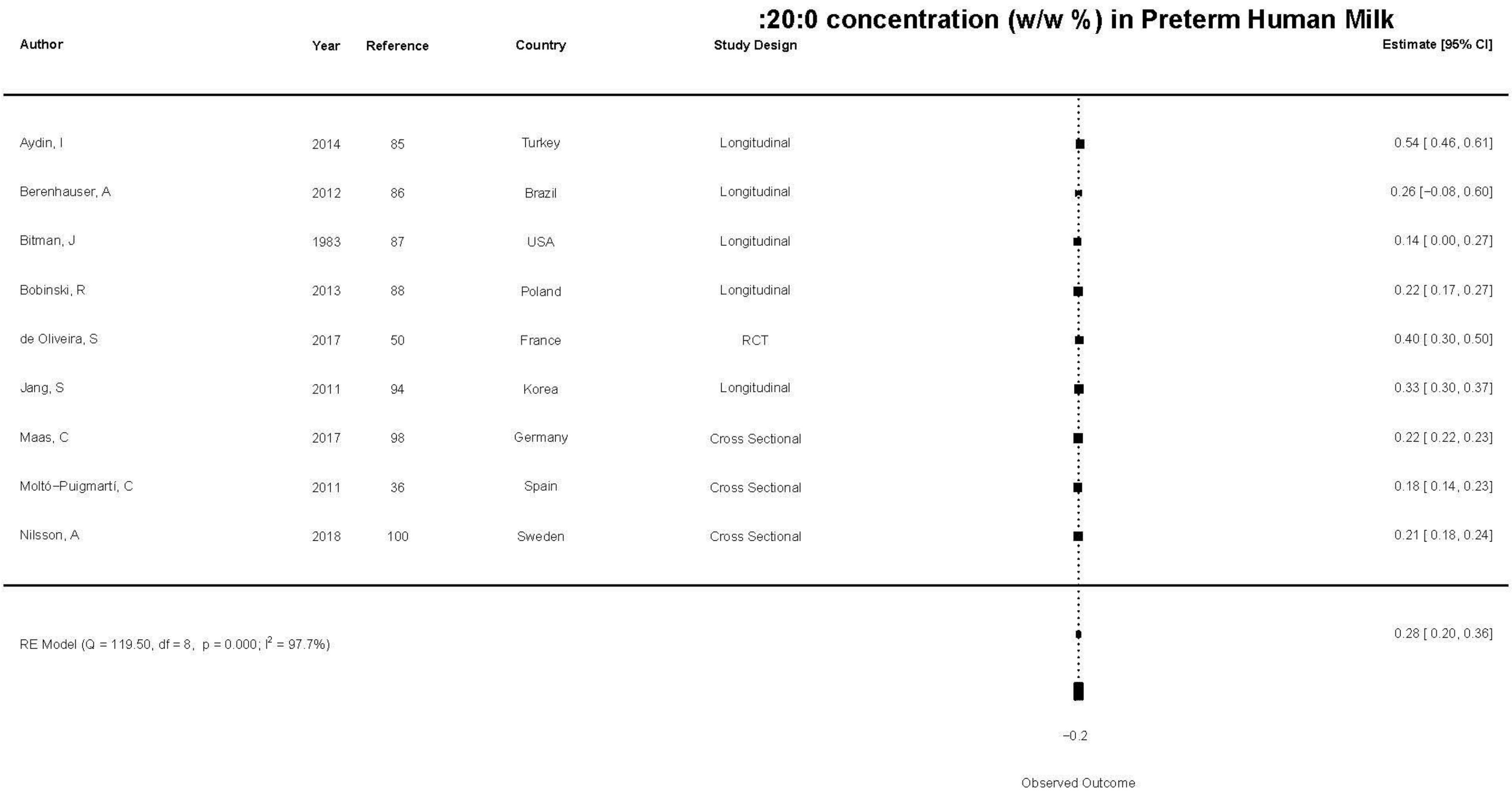
:20:0 concentration (w/w %) in Preterm Human Milk
Author
Year
Reference
Country
Study Design
Estimate [95% CI]
Aydin, I 2014 85 Turkey Longitudinal 0.54 [0.46, 0.61]
Berenhauser, A 2012 86 Brazil Longitudinal 0.26 [−0.08, 0.60]
Bitman, J 1983 87 USA Longitudinal 0.14 [0.00, 0.27]
Bobinski, R 2013 88 Poland Longitudinal 0.22 [0.17, 0.27]
de Oliveira, S 2017 50 France RCT 0.40 [0.30, 0.50]
Jang, S 2011 94 Korea Longitudinal 0.33 [0.30, 0.37]
Maas, C 2017 98 Germany Cross Sectional 0.22 [0.22, 0.23]
Moltó−Puigmartí, C 2011 36 Spain Cross Sectional 0.18 [0.14, 0.23]
Nilsson, A 2018 100 Sweden Cross Sectional 0.21 [0.18, 0.24]
RE Model (Q = 119.50, df = 8, p = 0.000; I² = 97.7%) 0.28 [0.20, 0.36]
−0.2
Observed Outcome

20:1

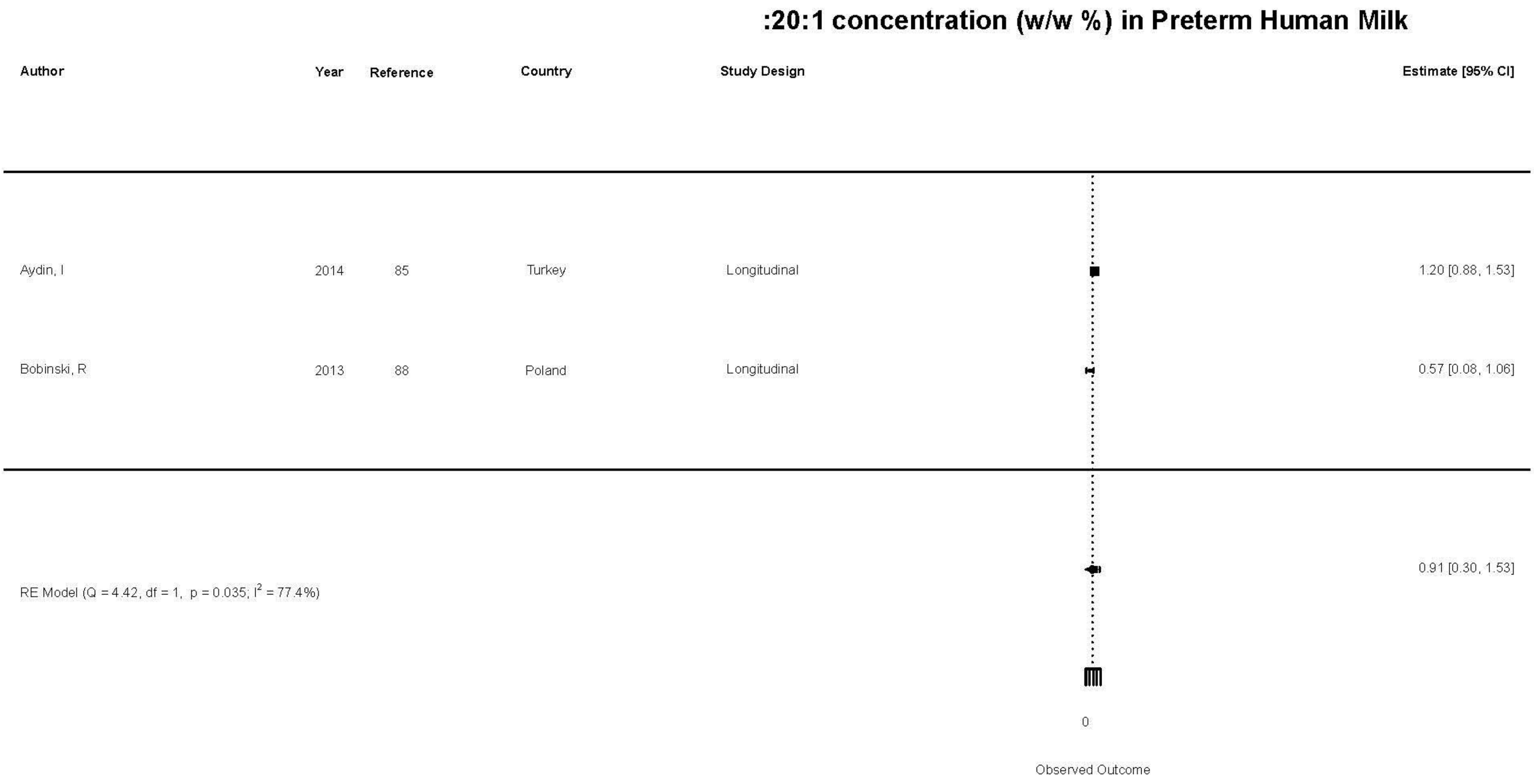
:20:1 concentration (w/w %) in Preterm Human Milk
Author
Year
Reference
Country
Study Design
Estimate [95% CI]
Aydin, I
2014
85
Turkey
Longitudinal
1.20 [0.88, 1.53]
Bobinski, R
2013
88
Poland
Longitudinal
0.57 [0.08, 1.06]
RE Model (Q = 4.42, df = 1, p = 0.035; I2 = 77.4%)
0.91 [0.30, 1.53]
0
Observed Outcome

20:1n-9

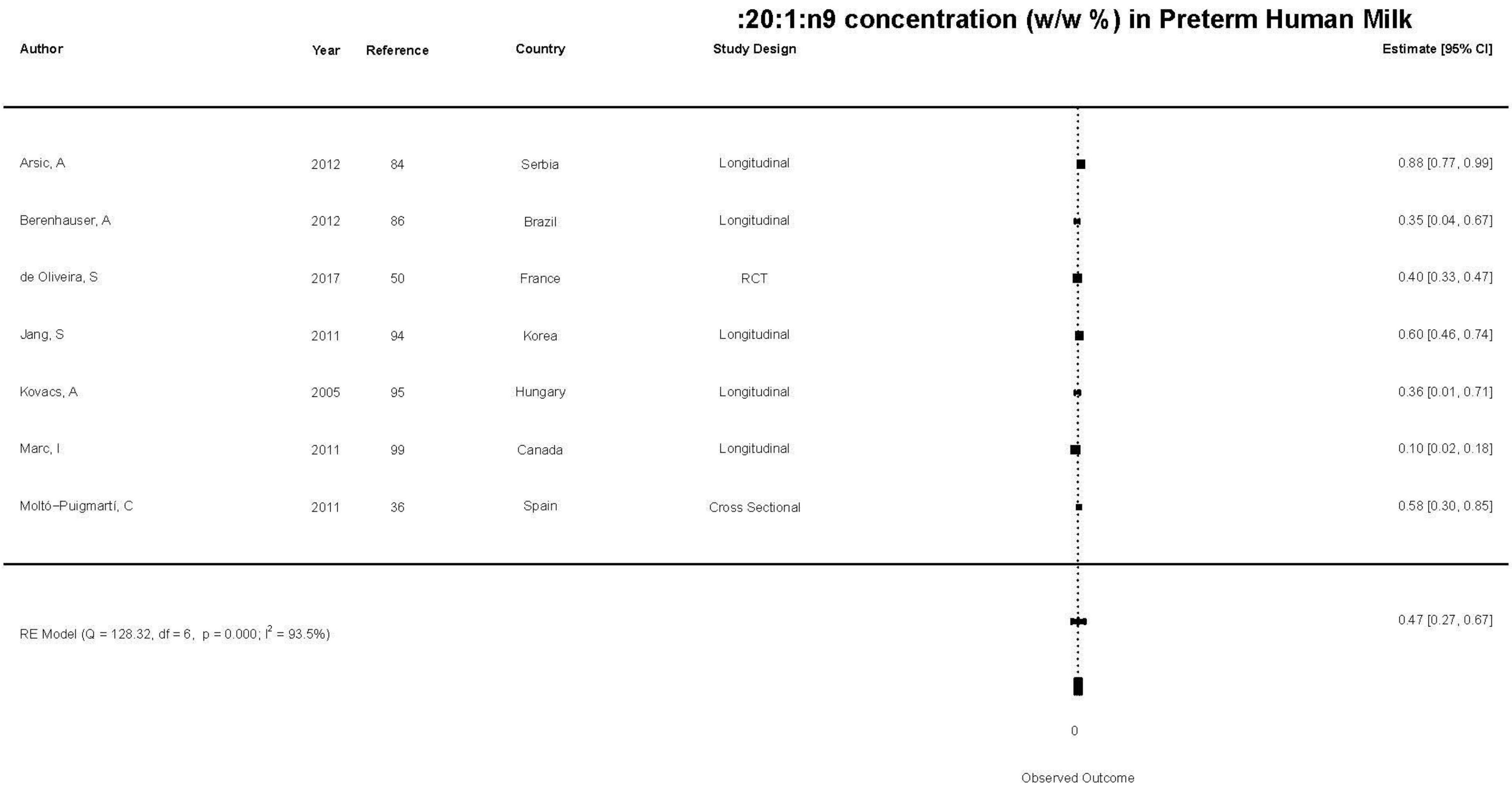
:20:1:n9 concentration (w/w %) in Preterm Human Milk
Author
Year
Reference
Country
Study Design
Estimate [95% CI]
Arsic, A 2012 84 Serbia Longitudinal 0.88 [0.77, 0.99]
Berenhauser, A 2012 86 Brazil Longitudinal 0.35 [0.04, 0.67]
de Oliveira, S 2017 50 France RCT 0.40 [0.33, 0.47]
Jang, S 2011 94 Korea Longitudinal 0.60 [0.46, 0.74]
Kovacs, A 2005 95 Hungary Longitudinal 0.36 [0.01, 0.71]
Marc, I 2011 99 Canada Longitudinal 0.10 [0.02, 0.18]
Moltó−Puigmartí, C 2011 36 Spain Cross Sectional 0.58 [0.30, 0.85]
RE Model (Q = 128.32, df = 6, p = 0.000; I2 = 93.5%) 0.47 [0.27, 0.67]
0
Observed Outcome

20:2

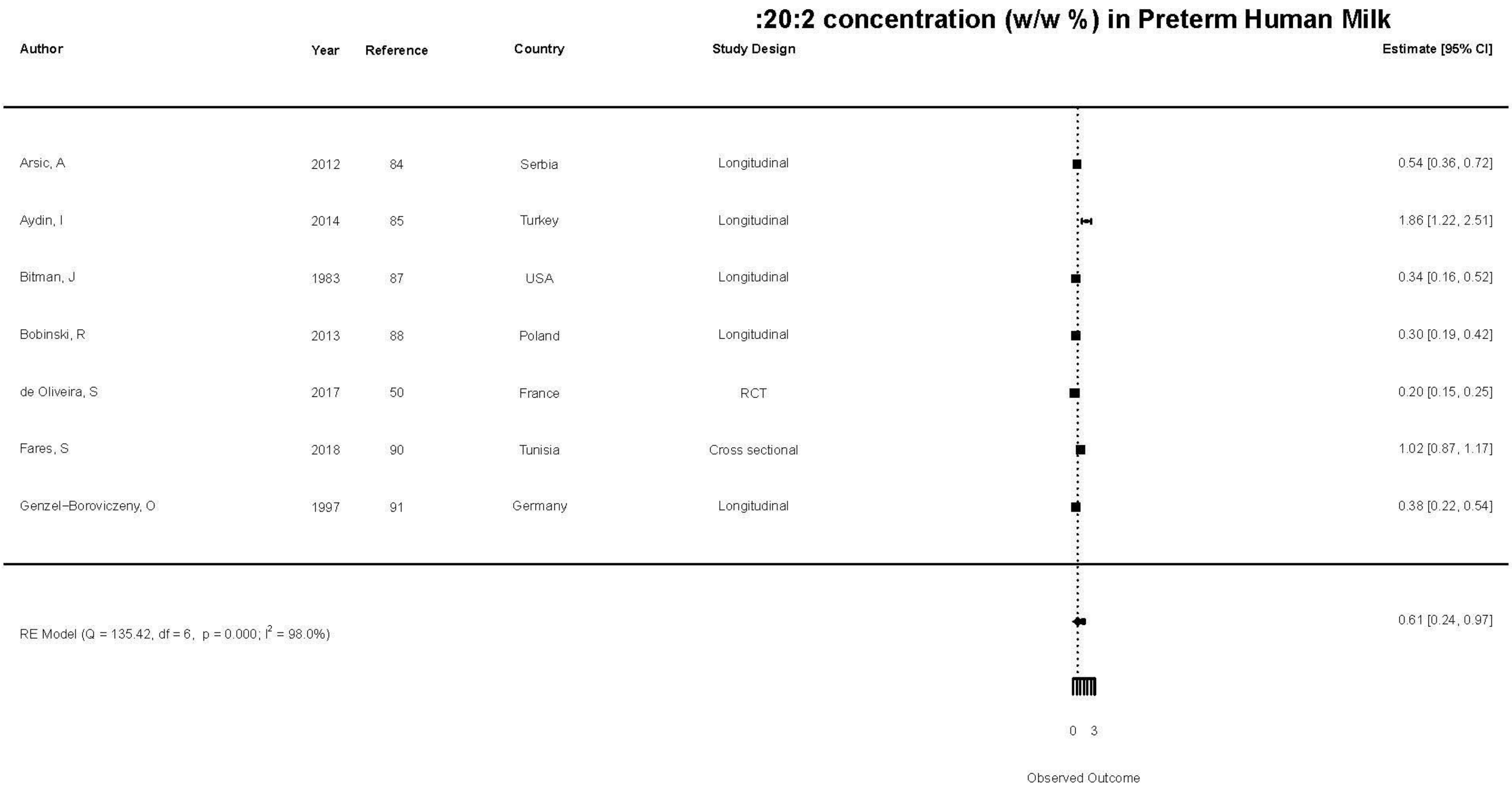
:20:2 concentration (w/w %) in Preterm Human Milk
Author
Year
Reference
Country
Study Design
Estimate [95% CI]
Arsic, A
2012
84
Serbia
Longitudinal
0.54 [0.36, 0.72]
Aydin, I
2014
85
Turkey
Longitudinal
1.86 [1.22, 2.51]
Bitman, J
1983
87
USA
Longitudinal
0.34 [0.16, 0.52]
Bobinski, R
2013
88
Poland
Longitudinal
0.30 [0.19, 0.42]
de Oliveira, S
2017
50
France
RCT
0.20 [0.15, 0.25]
Fares, S
2018
90
Tunisia
Cross sectional
1.02 [0.87, 1.17]
Genzel−Boroviczeny, O
1997
91
Germany
Longitudinal
0.38 [0.22, 0.54]
RE Model (Q = 135.42, df = 6, p = 0.000; I2 = 98.0%)
0.61 [0.24, 0.97]
0
3
Observed Outcome

Corresponding Author, Ariana Bailey

20:2 n-6

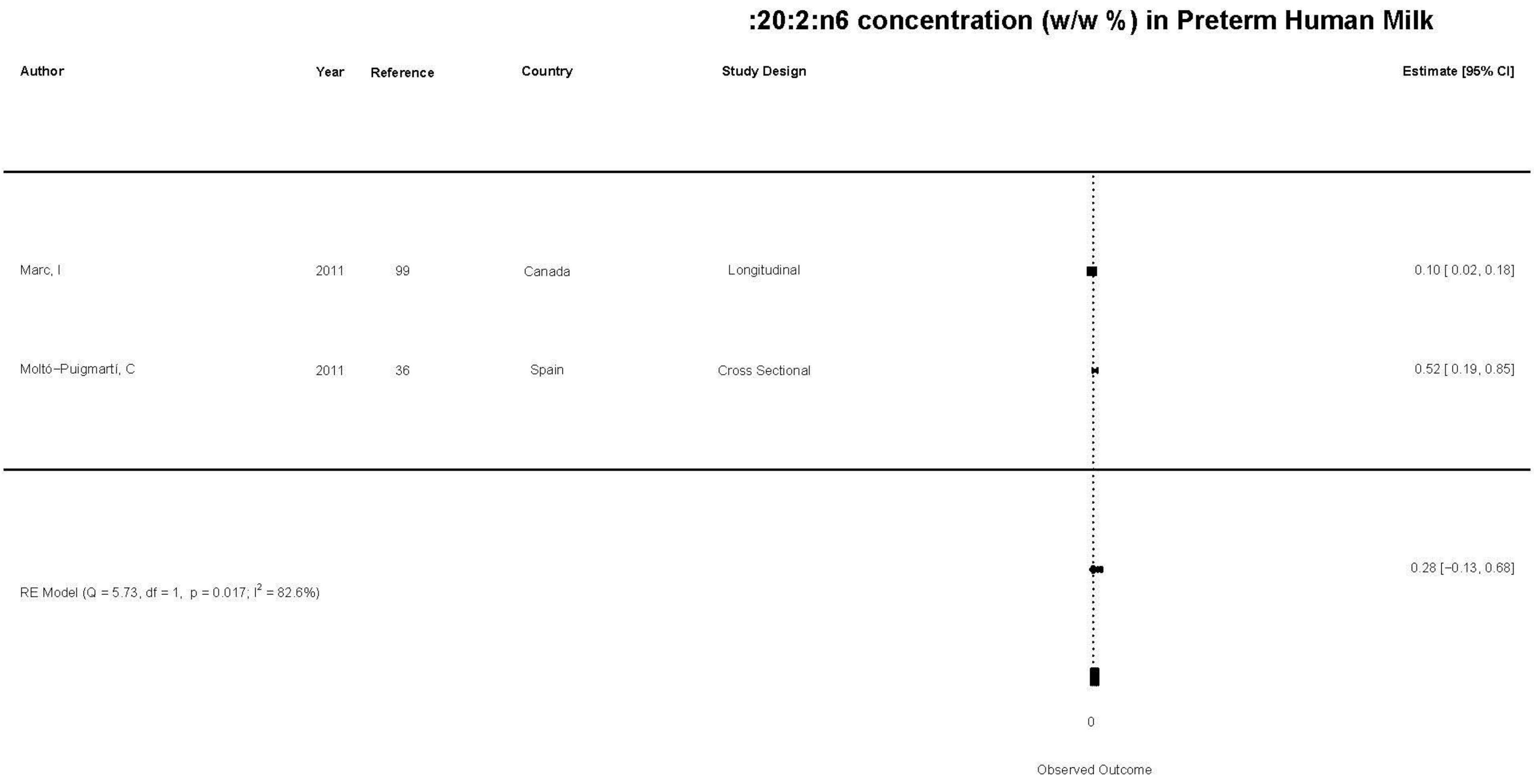

Total Fat and Fatty Acid Composition of Preterm Human Milk: A Systematic Review and Meta- Analysis

Corresponding Author, Ariana Bailey

20:3 n-3

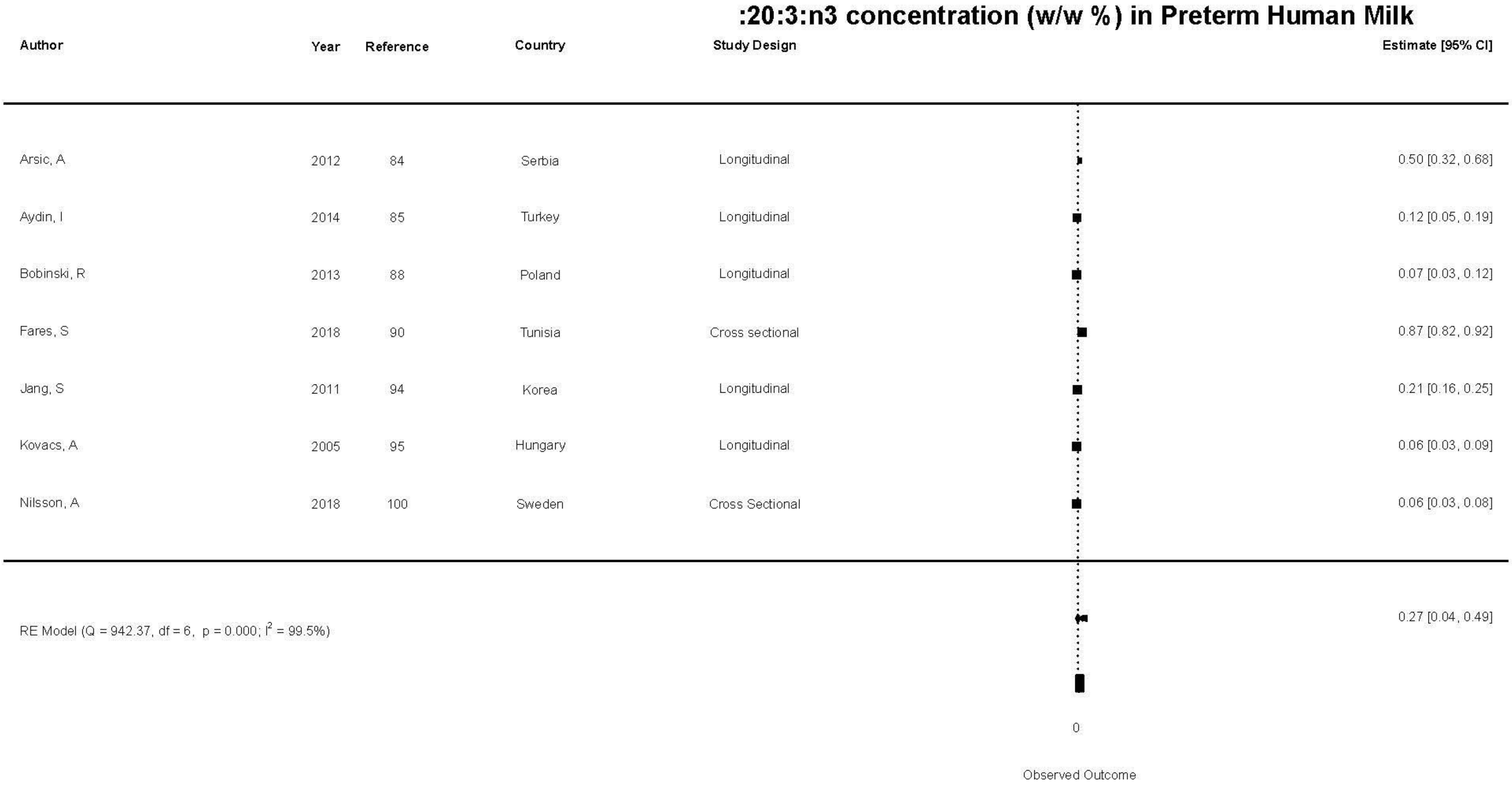

20:3 n-6

:20:3:n6 concentration (w/w %) in Preterm Human Milk

Author	Year	Reference	Country	Study Design	Estimate [95% CI]
Arsic, A	2012	84	Serbia	Longitudinal	0.50 [0.32, 0.68]
Bobinski, R	2013	88	Poland	Longitudinal	0.44 [0.30, 0.58]
de Oliveira, S	2017	50	France	RCT	0.30 [0.25, 0.35]
Fares, S	2018	90	Tunisia	Cross sectional	0.87 [0.82, 0.92]
Genzel−Boroviczeny, O	1997	91	Germany	Longitudinal	0.43 [0.36, 0.49]
Jang, S	2011	94	Korea	Longitudinal	0.57 [0.47, 0.66]
Kovacs, A	2005	95	Hungary	Longitudinal	0.47 [0.27, 0.67]
Luukkainen, P	1994	97	Finland	Longitudinal	0.30 [0.17, 0.43]
Maas, C	2017	98	Germany	Cross Sectional	0.83 [0.81, 0.85]
Marc, I	2011	99	Canada	Longitudinal	0.30 [0.17, 0.43]
Moltó−Puigmartí, C	2011	36	Spain	Cross Sectional	0.64 [0.39, 0.88]
Nilsson, A	2018	100	Sweden	Cross Sectional	0.26 [0.07, 0.44]
Sabel, K	2009	101	Sweden	Cross sectional	0.50 [0.46, 0.54]
RE Model ($Q = 752.86$, $df = 12$, $p = 0.000$; $I^2 = 97.4\%$)					0.50 [0.39, 0.61]

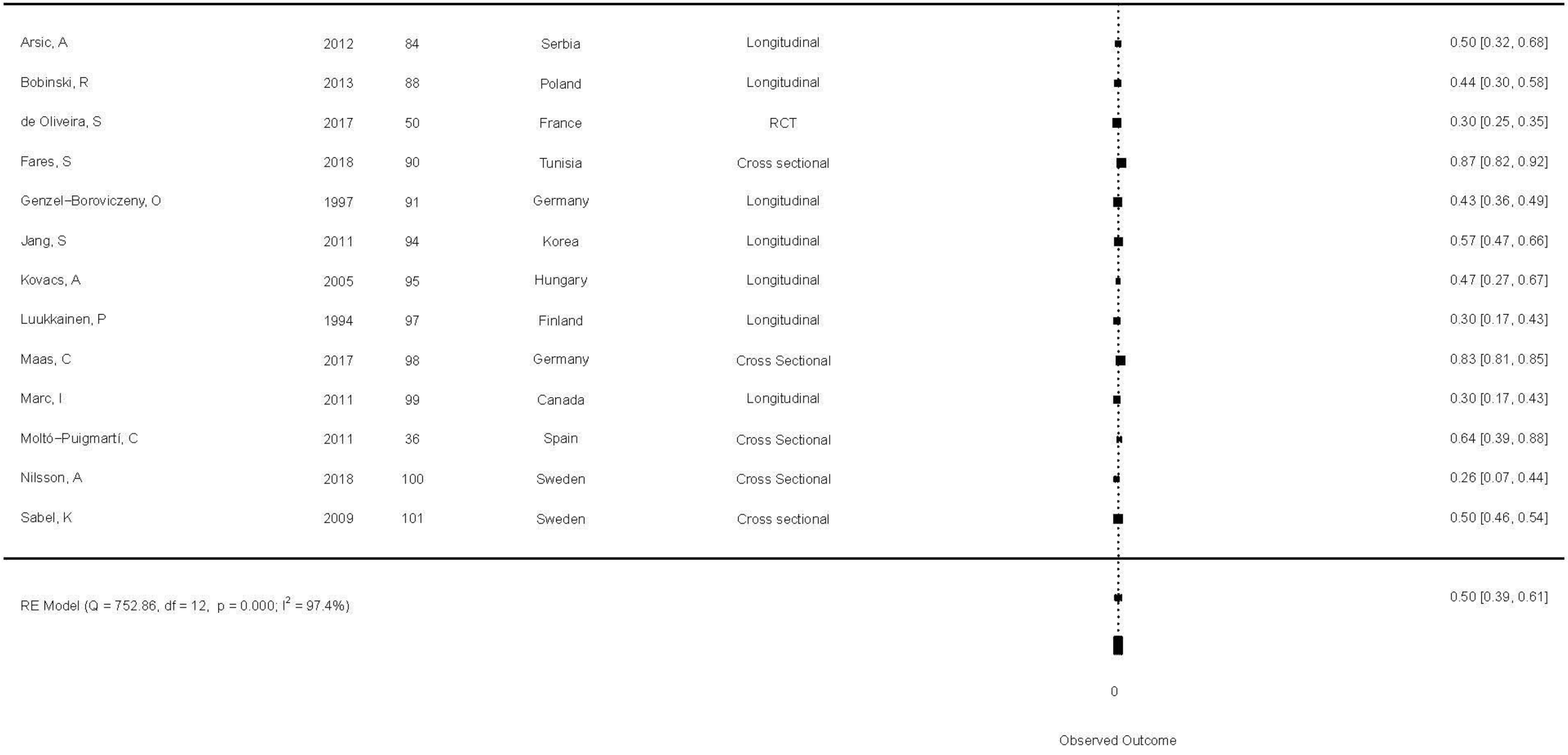

20:4 n-6

:20:4:n6 concentration (w/w %) in Preterm Human Milk

Author	Year	Reference	Country	Study Design	Estimate [95% CI]
Arsic, A	2012	84	Serbia	Longitudinal	0.52 [0.44, 0.60]
Aydin, I	2014	85	Turkey	Longitudinal	1.52 [1.08, 1.97]
Berenhauser, A	2012	86	Brazil	Longitudinal	0.55 [0.23, 0.86]
Bobinski, R	2013	88	Poland	Longitudinal	0.65 [0.43, 0.86]
Castillo, F	2021	89	Spain	Longitudinal	0.96 [0.64, 1.27]
de Oliveira, S	2017	50	France	RCT	0.10 [0.05, 0.15]
Fares, S	2018	90	Tunisia	Cross sectional	1.17 [1.07, 1.26]
Genzel−Boroviczeny, O	1997	91	Germany	Longitudinal	0.52 [0.35, 0.69]
Hossain, Z	2016	92	Canada	Longitudinal	0.50 [0.42, 0.58]
Innis, S	1990	93	Canada	Longitudinal	0.80 [0.56, 1.04]
Jang, S	2011	94	Korea	Longitudinal	0.75 [0.61, 0.89]
Kovacs, A	2005	95	Hungary	Longitudinal	0.64 [0.26, 1.02]
Luukkainen, P	1994	97	Finland	Longitudinal	0.40 [0.25, 0.55]
Maas, C	2017	98	Germany	Cross Sectional	0.82 [0.80, 0.83]
Marc, I	2011	99	Canada	Longitudinal	0.30 [0.17, 0.43]
Moltó−Puigmartí, C	2011	36	Spain	Cross Sectional	0.97 [0.69, 1.26]
Sabel, K	2009	101	Sweden	Cross sectional	0.61 [0.59, 0.64]
Xu, Y	2018	102	China	Longitudinal	0.31 [0.19, 0.43]
RE Model (Q = 1051.05, df = 17, p = 0.000; I^2 = 98.6%)					0.65 [0.50, 0.80]

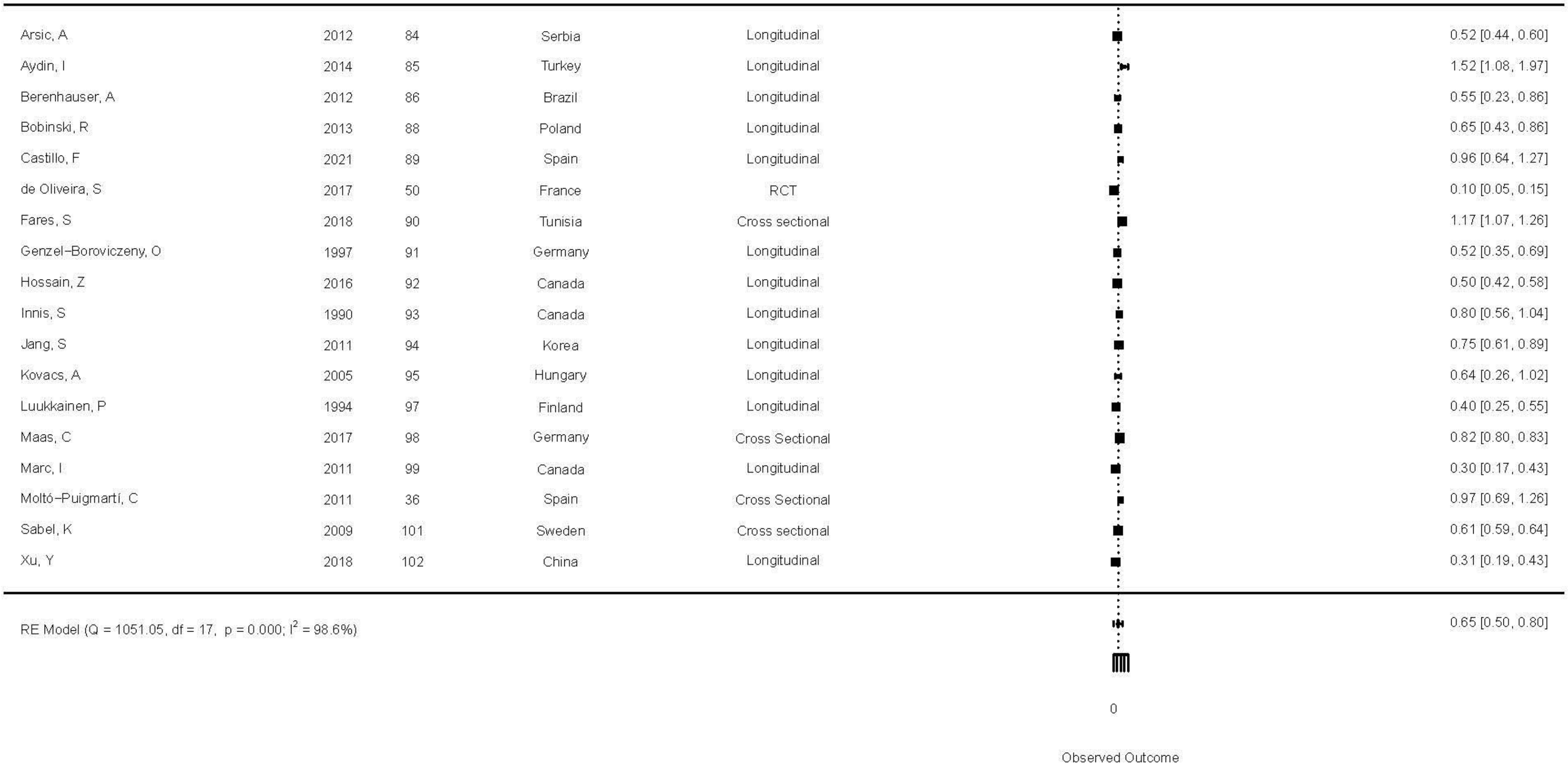

20:5 n-3

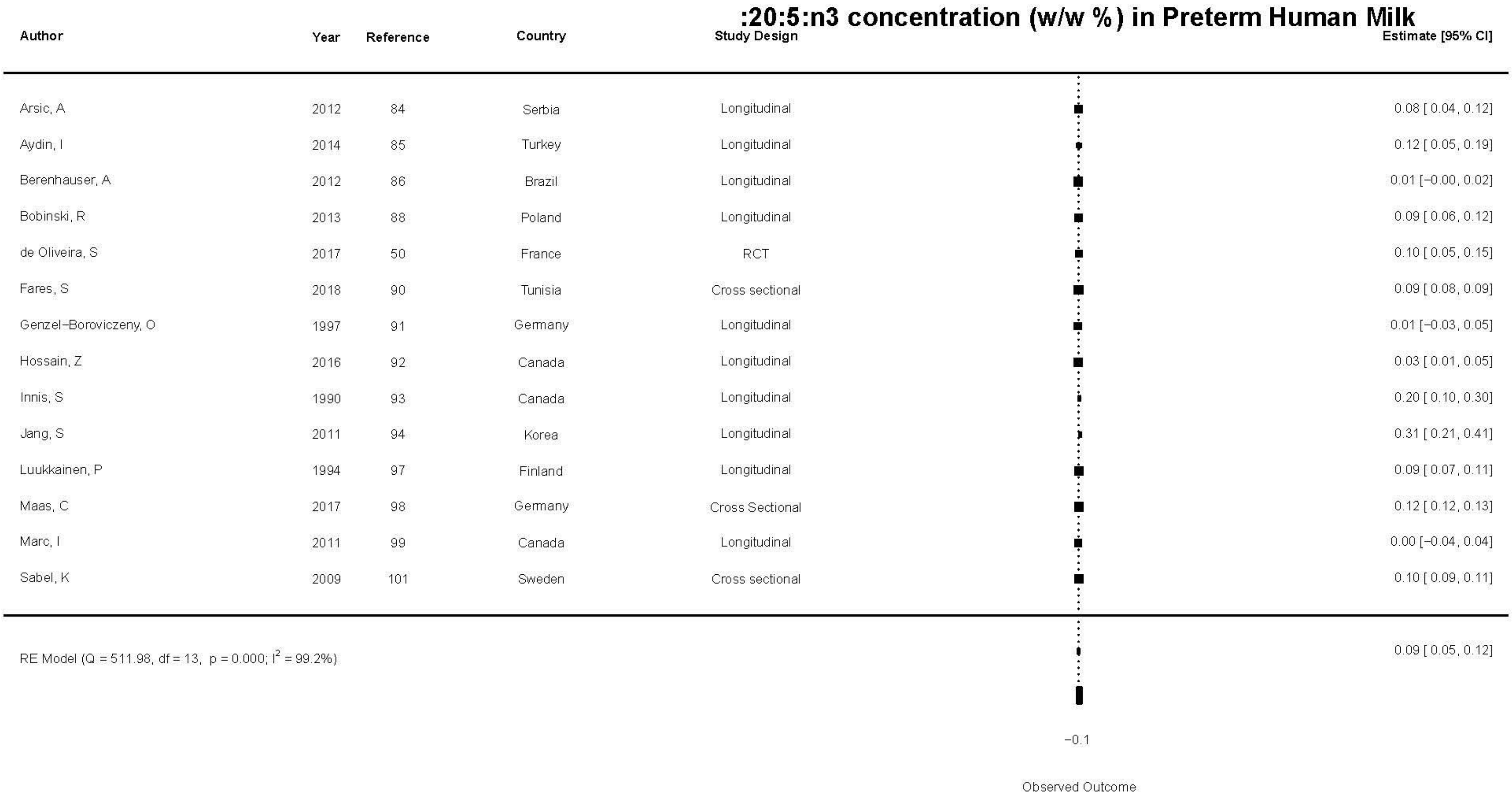
:20:5:n3 concentration (w/w %) in Preterm Human Milk
Author | Year | Reference | Country | Study Design | Estimate [95% CI]
Arsic, A | 2012 | 84 | Serbia | Longitudinal | 0.08 [0.04, 0.12]
Aydin, I | 2014 | 85 | Turkey | Longitudinal | 0.12 [0.05, 0.19]
Berenhauser, A | 2012 | 86 | Brazil | Longitudinal | 0.01 [−0.00, 0.02]
Bobinski, R | 2013 | 88 | Poland | Longitudinal | 0.09 [0.06, 0.12]
de Oliveira, S | 2017 | 50 | France | RCT | 0.10 [0.05, 0.15]
Fares, S | 2018 | 90 | Tunisia | Cross sectional | 0.09 [0.08, 0.09]
Genzel−Boroviczeny, O | 1997 | 91 | Germany | Longitudinal | 0.01 [−0.03, 0.05]
Hossain, Z | 2016 | 92 | Canada | Longitudinal | 0.03 [0.01, 0.05]
Innis, S | 1990 | 93 | Canada | Longitudinal | 0.20 [0.10, 0.30]
Jang, S | 2011 | 94 | Korea | Longitudinal | 0.31 [0.21, 0.41]
Luukkainen, P | 1994 | 97 | Finland | Longitudinal | 0.09 [0.07, 0.11]
Maas, C | 2017 | 98 | Germany | Cross Sectional | 0.12 [0.12, 0.13]
Marc, I | 2011 | 99 | Canada | Longitudinal | 0.00 [−0.04, 0.04]
Sabel, K | 2009 | 101 | Sweden | Cross sectional | 0.10 [0.09, 0.11]
RE Model (Q = 511.98, df = 13, p = 0.000; I^2 = 99.2%) | 0.09 [0.05, 0.12]
−0.1
Observed Outcome

22:0

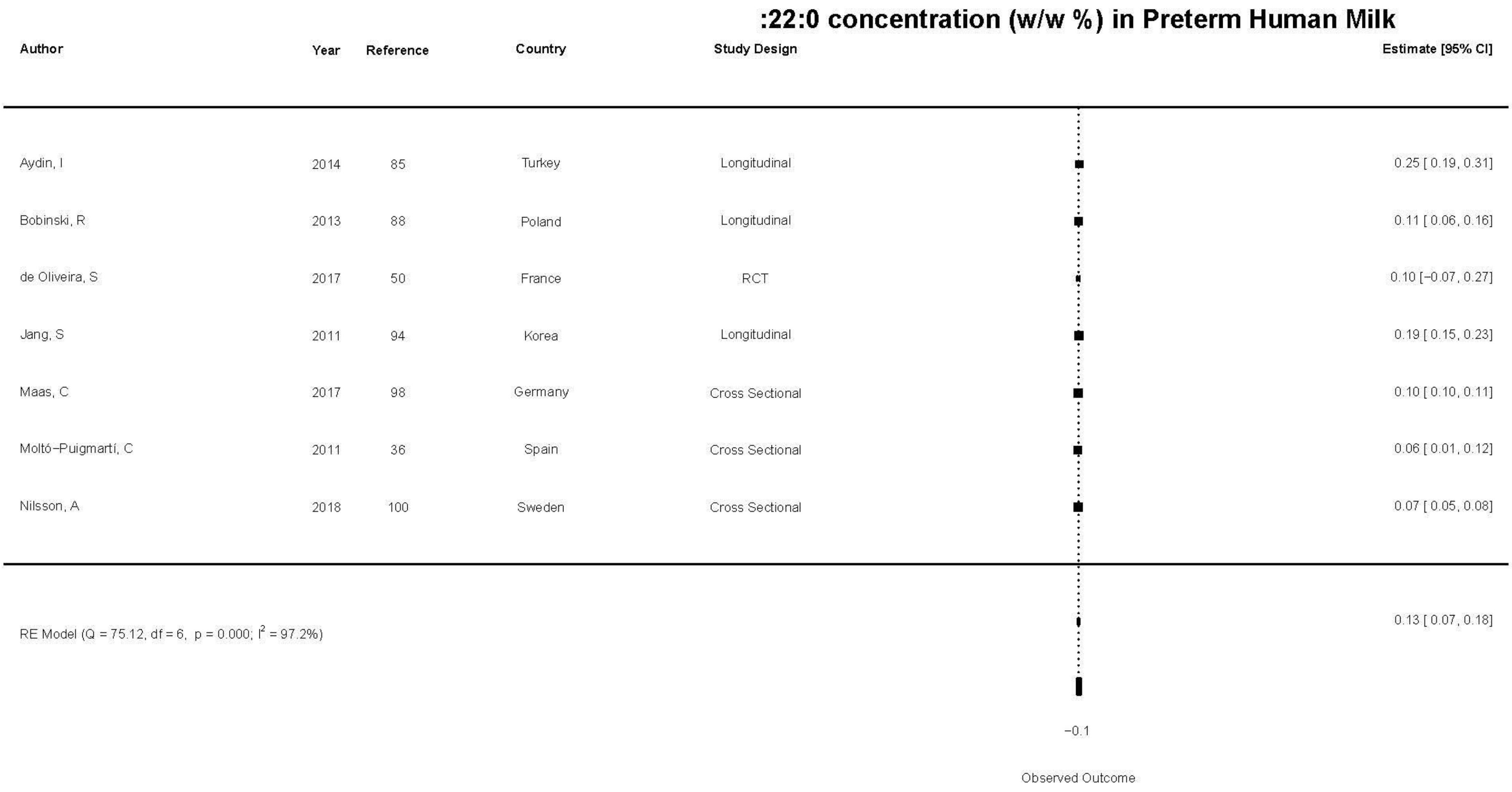
:22:0 concentration (w/w %) in Preterm Human Milk
Author | Year | Reference | Country | Study Design | Estimate [95% CI]
Aydin, I | 2014 | 85 | Turkey | Longitudinal | 0.25 [0.19, 0.31]
Bobinski, R | 2013 | 88 | Poland | Longitudinal | 0.11 [0.06, 0.16]
de Oliveira, S | 2017 | 50 | France | RCT | 0.10 [−0.07, 0.27]
Jang, S | 2011 | 94 | Korea | Longitudinal | 0.19 [0.15, 0.23]
Maas, C | 2017 | 98 | Germany | Cross Sectional | 0.10 [0.10, 0.11]
Moltó−Puigmartí, C | 2011 | 36 | Spain | Cross Sectional | 0.06 [0.01, 0.12]
Nilsson, A | 2018 | 100 | Sweden | Cross Sectional | 0.07 [0.05, 0.08]
RE Model (Q = 75.12, df = 6, p = 0.000; I² = 97.2%) | 0.13 [0.07, 0.18]
−0.1
Observed Outcome

22:1 n-9

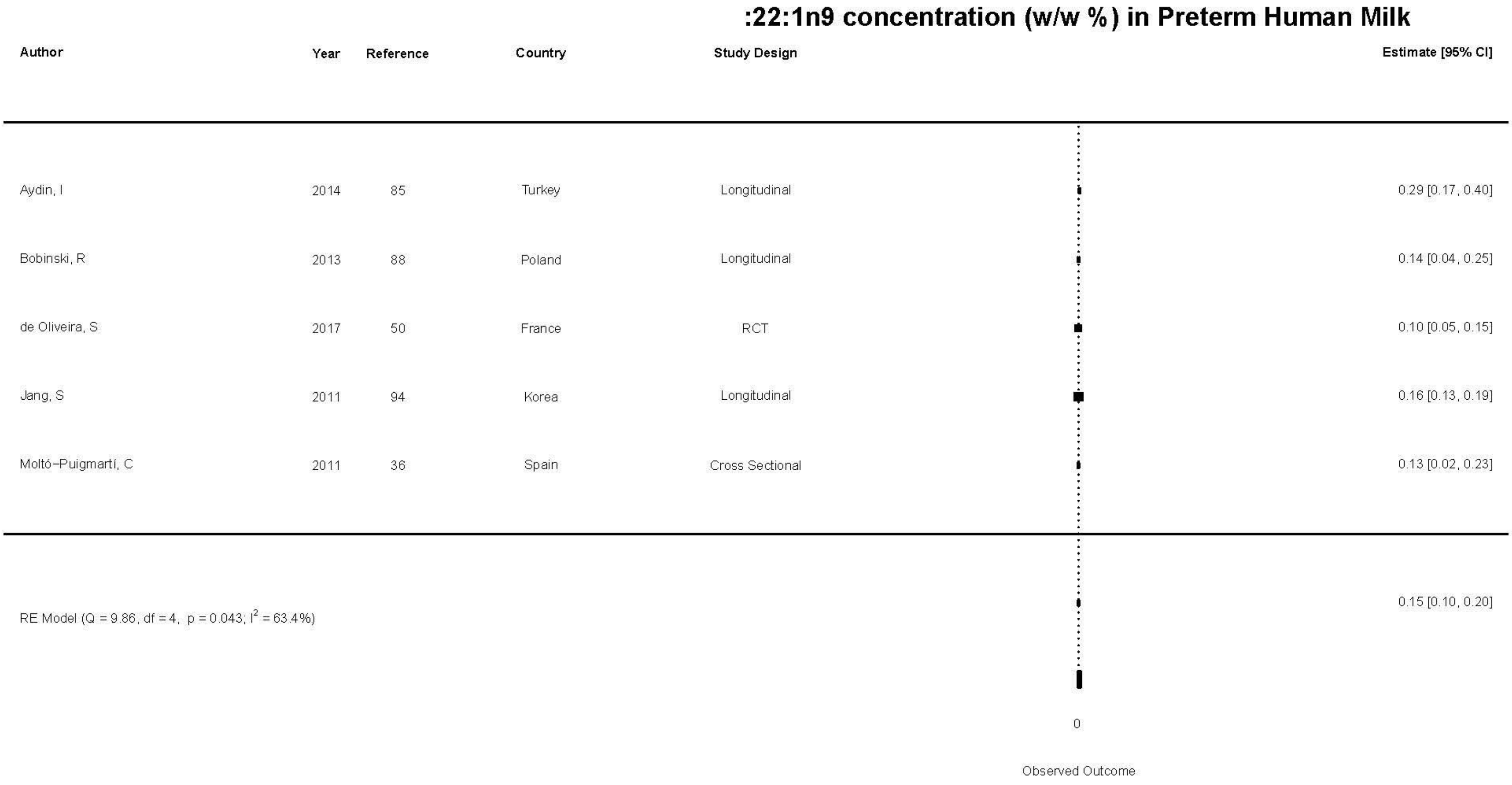
:22:1n9 concentration (w/w %) in Preterm Human Milk
Author
Year
Reference
Country
Study Design
Estimate [95% CI]
Aydin, I
2014
85
Turkey
Longitudinal
0.29 [0.17, 0.40]
Bobinski, R
2013
88
Poland
Longitudinal
0.14 [0.04, 0.25]
de Oliveira, S
2017
50
France
RCT
0.10 [0.05, 0.15]
Jang, S
2011
94
Korea
Longitudinal
0.16 [0.13, 0.19]
Moltó−Puigmartí, C
2011
36
Spain
Cross Sectional
0.13 [0.02, 0.23]
RE Model (Q = 9.86, df = 4, p = 0.043; I^2 = 63.4%)
0.15 [0.10, 0.20]
0
Observed Outcome

22:5 n-3

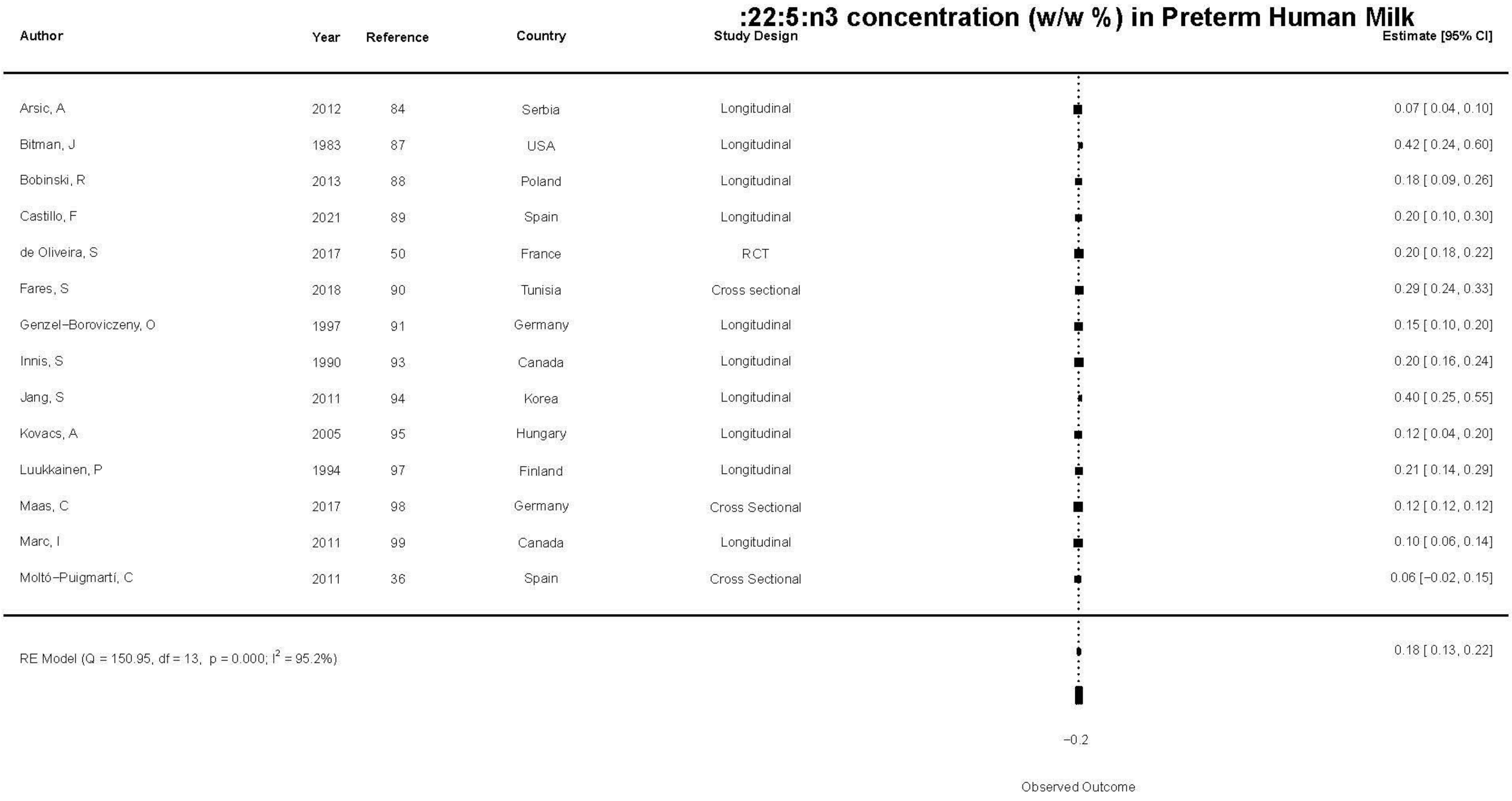
:22:5:n3 concentration (w/w %) in Preterm Human Milk
Author
Year
Reference
Country
Study Design
Estimate [95% CI]
Arsic, A 2012 84 Serbia Longitudinal 0.07 [0.04, 0.10]
Bitman, J 1983 87 USA Longitudinal 0.42 [0.24, 0.60]
Bobinski, R 2013 88 Poland Longitudinal 0.18 [0.09, 0.26]
Castillo, F 2021 89 Spain Longitudinal 0.20 [0.10, 0.30]
de Oliveira, S 2017 50 France RCT 0.20 [0.18, 0.22]
Fares, S 2018 90 Tunisia Cross sectional 0.29 [0.24, 0.33]
Genzel−Boroviczeny, O 1997 91 Germany Longitudinal 0.15 [0.10, 0.20]
Innis, S 1990 93 Canada Longitudinal 0.20 [0.16, 0.24]
Jang, S 2011 94 Korea Longitudinal 0.40 [0.25, 0.55]
Kovacs, A 2005 95 Hungary Longitudinal 0.12 [0.04, 0.20]
Luukkainen, P 1994 97 Finland Longitudinal 0.21 [0.14, 0.29]
Maas, C 2017 98 Germany Cross Sectional 0.12 [0.12, 0.12]
Marc, I 2011 99 Canada Longitudinal 0.10 [0.06, 0.14]
Moltó−Puigmartí, C 2011 36 Spain Cross Sectional 0.06 [−0.02, 0.15]
RE Model (Q = 150.95, df = 13, p = 0.000; I2 = 95.2%) 0.18 [0.13, 0.22]
−0.2
Observed Outcome

22:5 n-6

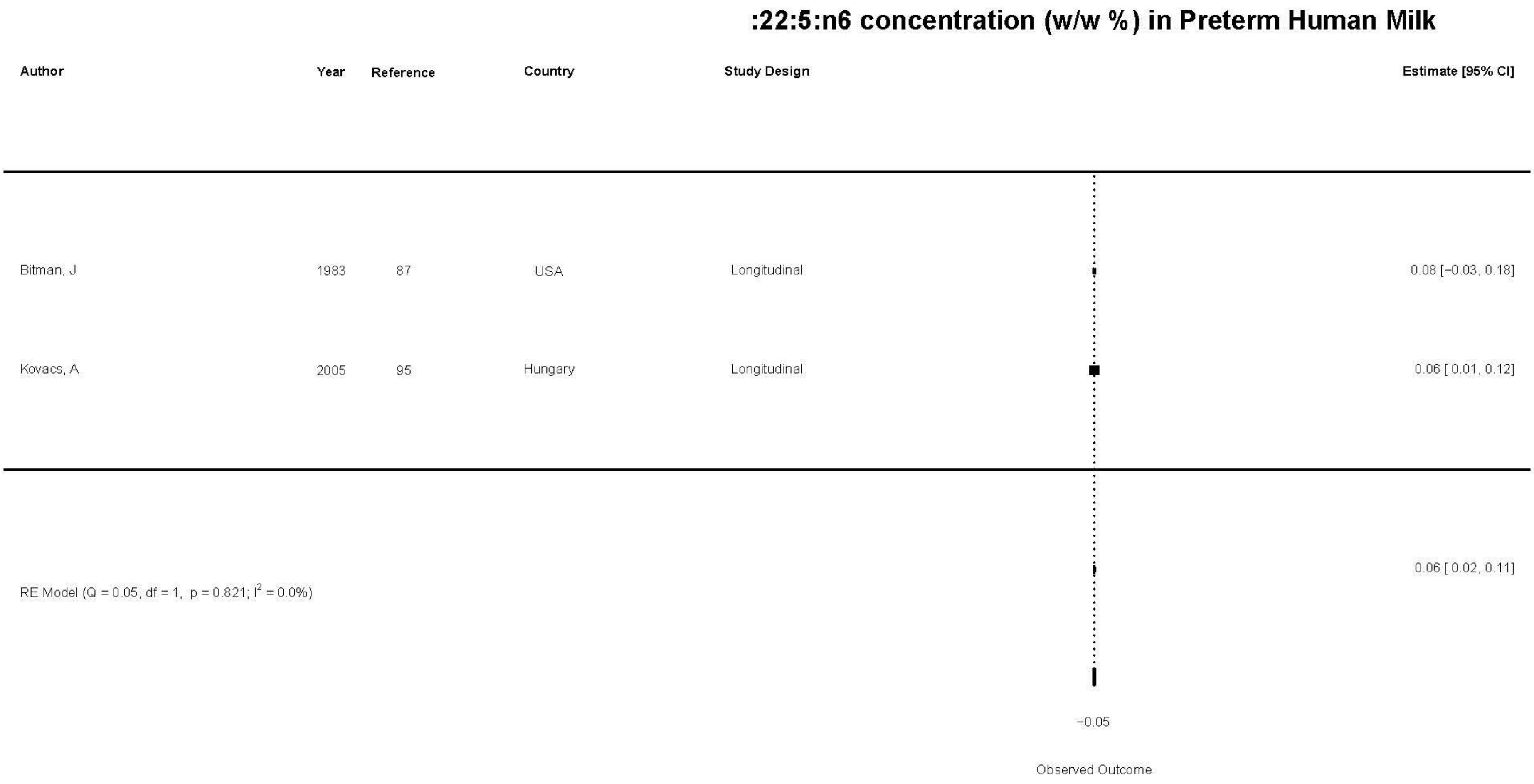
:22:5:n6 concentration (w/w %) in Preterm Human Milk
Author
Year
Reference
Country
Study Design
Estimate [95% CI]
Bitman, J
1983
87
USA
Longitudinal
0.08 [−0.03, 0.18]
Kovacs, A
2005
95
Hungary
Longitudinal
0.06 [0.01, 0.12]
RE Model (Q = 0.05, df = 1, p = 0.821; I2 = 0.0%)
0.06 [0.02, 0.11]
−0.05
Observed Outcome

22:6 n-3

:22:6:n3 concentration (w/w %) in Preterm Human Milk

Author	Year	Reference	Country	Study Design	Estimate [95% CI]
Arsic, A	2012	84	Serbia	Longitudinal	0.19 [0.15, 0.24]
Aydin, I	2014	85	Turkey	Longitudinal	0.89 [0.55, 1.23]
Berenhauser, A	2012	86	Brazil	Longitudinal	0.06 [0.01, 0.12]
Bobinski, R	2013	88	Poland	Longitudinal	0.39 [0.27, 0.52]
Castillo, F	2021	89	Spain	Longitudinal	0.75 [0.57, 0.93]
de Oliveira, S	2017	50	France	RCT	0.30 [0.25, 0.35]
Fares, S	2018	90	Tunisia	Cross sectional	0.58 [0.27, 0.89]
Hossain, Z	2016	92	Canada	Longitudinal	0.21 [0.15, 0.27]
Innis, S	1990	93	Canada	Longitudinal	0.50 [0.26, 0.74]
Jang, S	2011	94	Korea	Longitudinal	0.80 [0.60, 0.99]
Luukkainen, P	1994	97	Finland	Longitudinal	0.40 [0.26, 0.53]
Marc, I	2011	99	Canada	Longitudinal	0.10 [0.02, 0.18]
Sabel, K	2009	101	Sweden	Cross sectional	0.56 [0.51, 0.61]
Xu, Y	2018	102	China	Longitudinal	0.13 [0.09, 0.16]
RE Model (Q = 344.71, df = 13, p = 0.000; I^2 = 97.9%)					0.40 [0.26, 0.53]

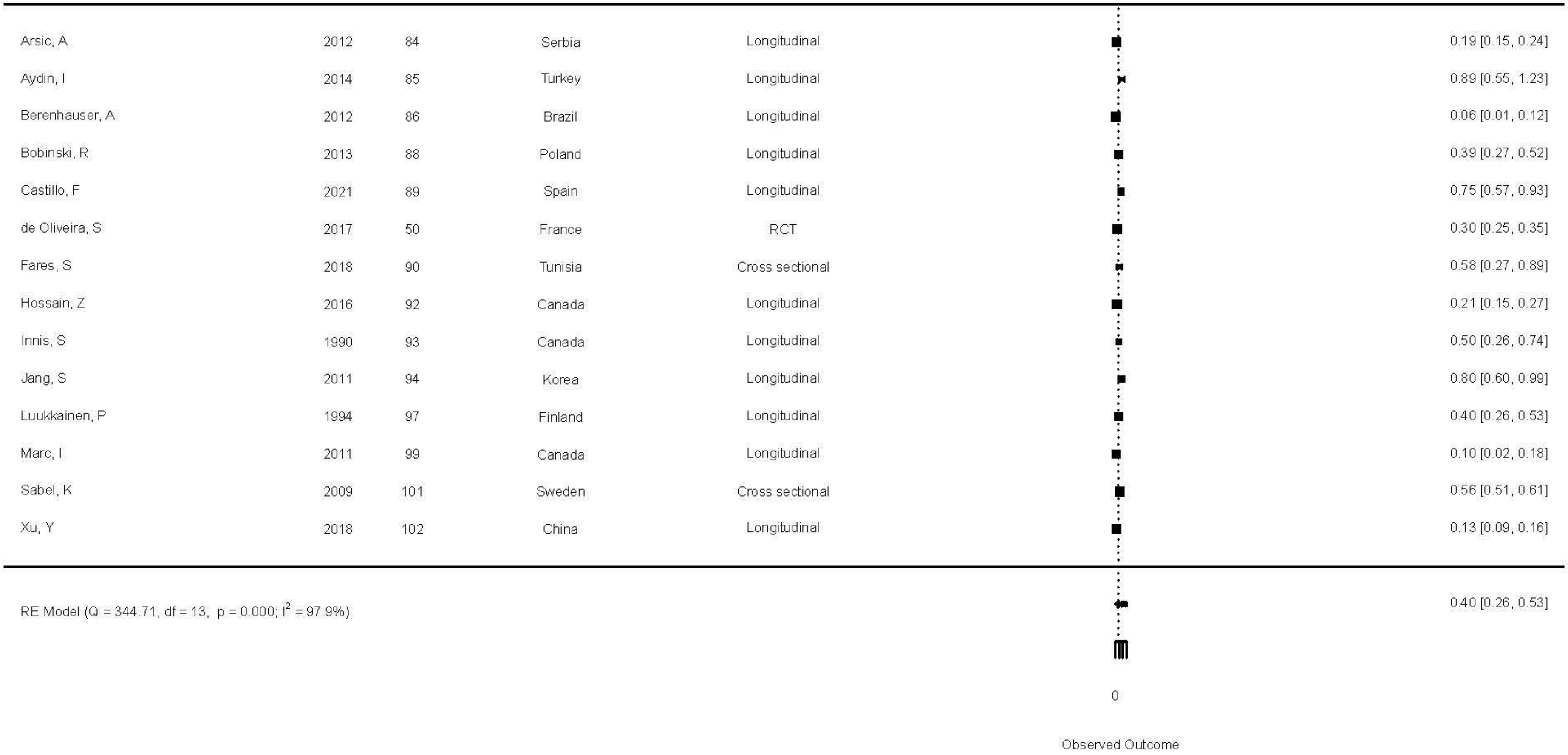

Corresponding Author, Ariana Bailey

23:0

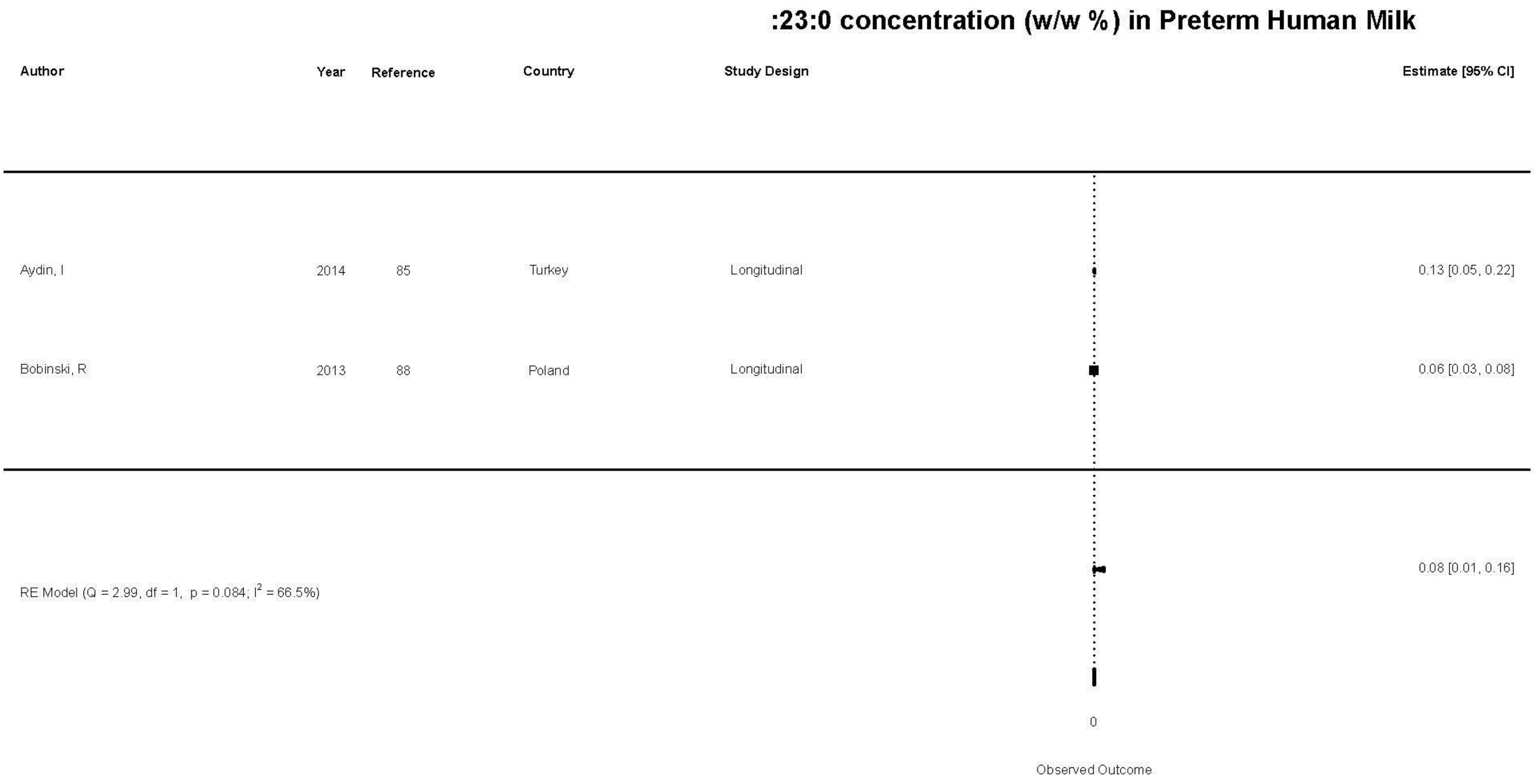

24:0

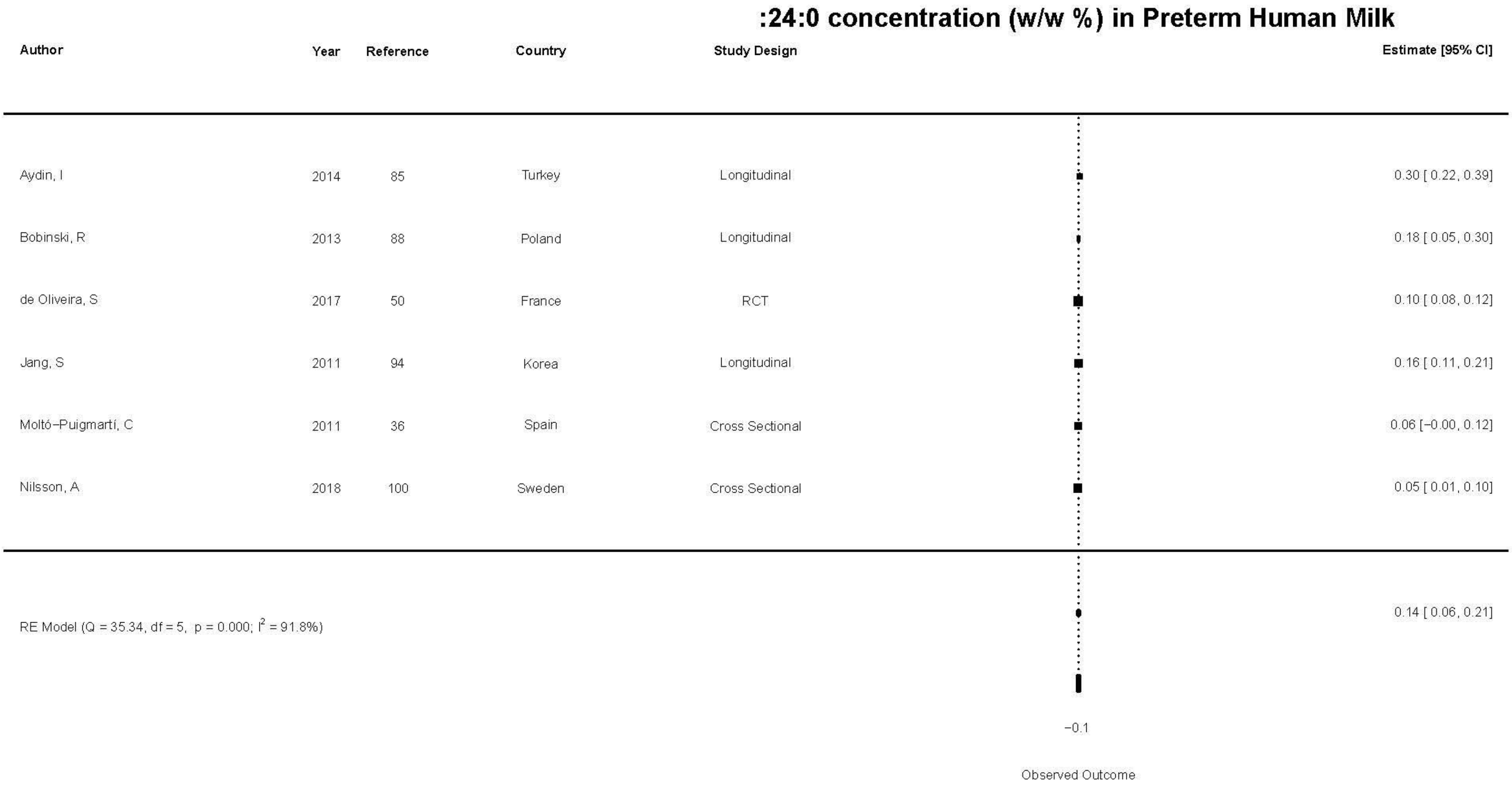
:24:0 concentration (w/w %) in Preterm Human Milk
Author
Year
Reference
Country
Study Design
Estimate [95% CI]
Aydin, I 2014 85 Turkey Longitudinal 0.30 [0.22, 0.39]
Bobinski, R 2013 88 Poland Longitudinal 0.18 [0.05, 0.30]
de Oliveira, S 2017 50 France RCT 0.10 [0.08, 0.12]
Jang, S 2011 94 Korea Longitudinal 0.16 [0.11, 0.21]
Moltó−Puigmartí, C 2011 36 Spain Cross Sectional 0.06 [−0.00, 0.12]
Nilsson, A 2018 100 Sweden Cross Sectional 0.05 [0.01, 0.10]
RE Model (Q = 35.34, df = 5, p = 0.000; I² = 91.8%) 0.14 [0.06, 0.21]
−0.1
Observed Outcome

24:1

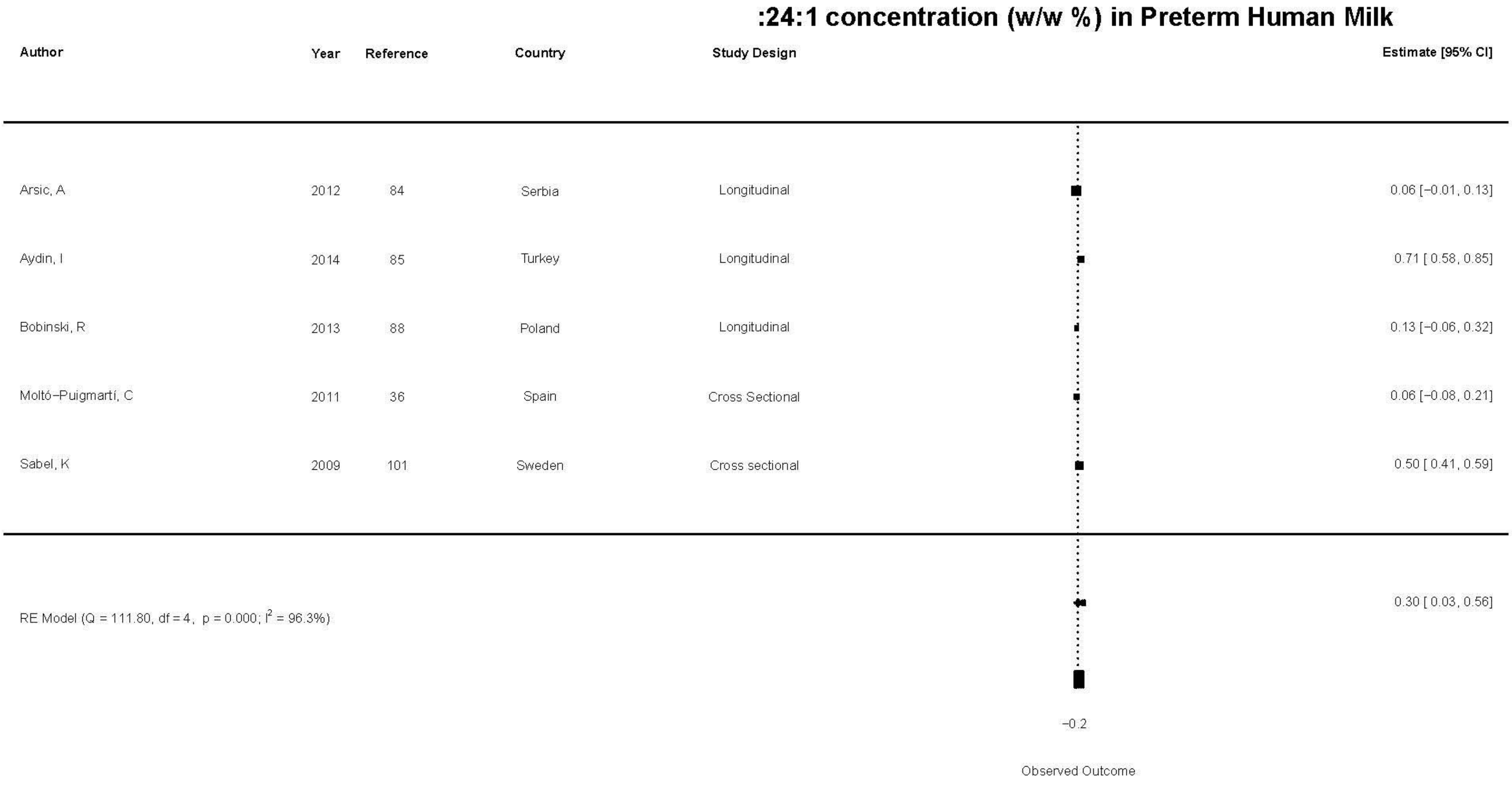
:24:1 concentration (w/w %) in Preterm Human Milk
Author
Year
Reference
Country
Study Design
Estimate [95% CI]
Arsic, A 2012 84 Serbia Longitudinal 0.06 [−0.01, 0.13]
Aydin, I 2014 85 Turkey Longitudinal 0.71 [0.58, 0.85]
Bobinski, R 2013 88 Poland Longitudinal 0.13 [−0.06, 0.32]
Moltó−Puigmartí, C 2011 36 Spain Cross Sectional 0.06 [−0.08, 0.21]
Sabel, K 2009 101 Sweden Cross sectional 0.50 [0.41, 0.59]
RE Model (Q = 111.80, df = 4, p = 0.000; I² = 96.3%) 0.30 [0.03, 0.56]
−0.2
Observed Outcome

24:1 n-9

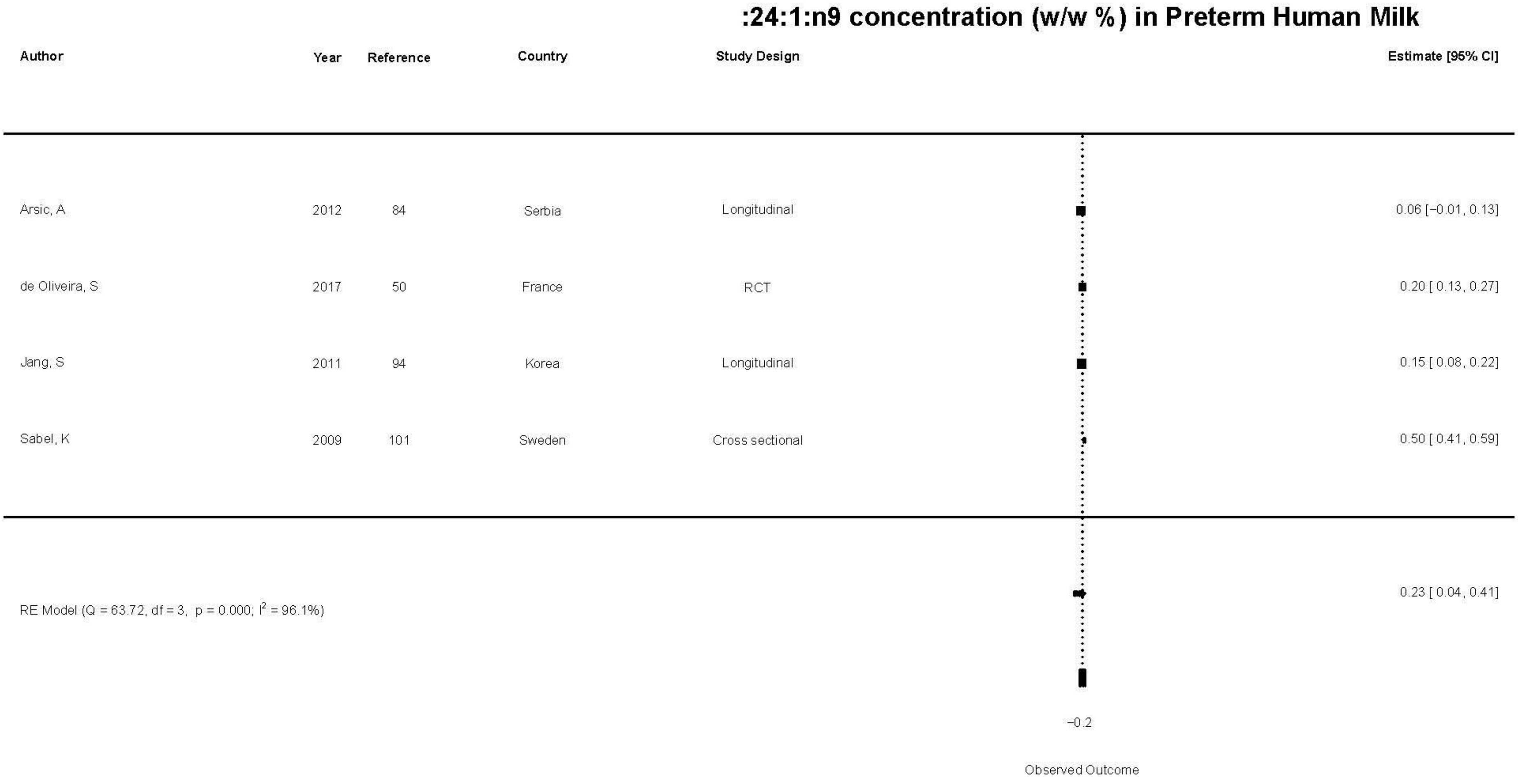
:24:1:n9 concentration (w/w %) in Preterm Human Milk
Author
Year
Reference
Country
Study Design
Estimate [95% CI]
Arsic, A
2012
84
Serbia
Longitudinal
0.06 [−0.01, 0.13]
de Oliveira, S
2017
50
France
RCT
0.20 [0.13, 0.27]
Jang, S
2011
94
Korea
Longitudinal
0.15 [0.08, 0.22]
Sabel, K
2009
101
Sweden
Cross sectional
0.50 [0.41, 0.59]
RE Model (Q = 63.72, df = 3, p = 0.000; I² = 96.1%)
0.23 [0.04, 0.41]
−0.2
Observed Outcome

Corresponding Author, Ariana Bailey

Supplementary Figure 6. Fat Analysis by Method, Creamatocrit

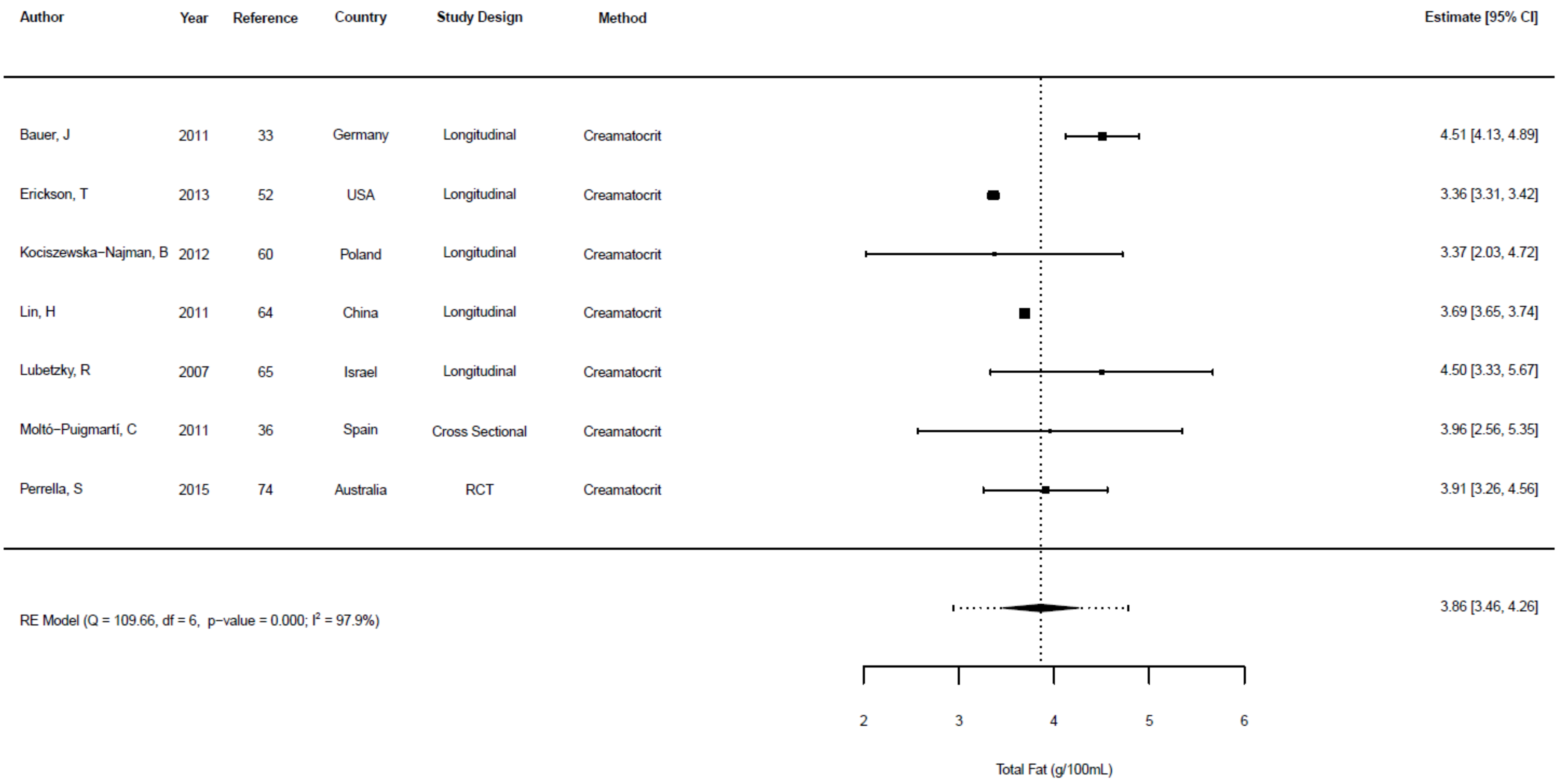

Supplementary Figure 7. Fat Analysis by Method, Gravimetric

Author	Year	Reference	Country	Study Design	Method	Estimate [95% CI]
Butte, N	1984	45	USA	Longitudinal	Gravimetric	3.86 [3.33, 4.39]
Campbell−Yeo, M	2010	46	Canada	RCT	Gravimetric	4.24 [3.93, 4.54]
Ehrenkranz, R	1984	51	USA	Longitudinal	Gravimetric	3.06 [1.59, 4.52]
Gates, A	2021	55	USA	Longitudinal	Gravimetric	3.36 [2.90, 3.81]
Groh−Wargo, S	2016	56	USA	Cross sectional	Gravimetric	3.13 [2.58, 3.68]
Gross, S	1980	57	USA	Longitudinal	Gravimetric	3.30 [1.64, 4.96]
Maas, Y	1998	66	Netherlands	Longitudinal	Gravimetric	3.54 [3.30, 3.77]
Meier, P	2002	69	USA	Longitudinal	Gravimetric	4.75 [2.44, 7.05]
Sauer, C	2017	79	USA	Longitudinal	Gravimetric	3.48 [3.08, 3.88]
Silber, G	1988	80	USA	Longitudinal	Gravimetric	3.94 [3.19, 4.69]
Smilowitz, J	2014	81	USA	Longitudinal	Gravimetric	3.10 [2.61, 3.59]
RE Model ($Q = 28.33$, $df = 10$, p−value = 0.002; $I^2 = 64.5\%$)						3.58 [3.31, 3.85]

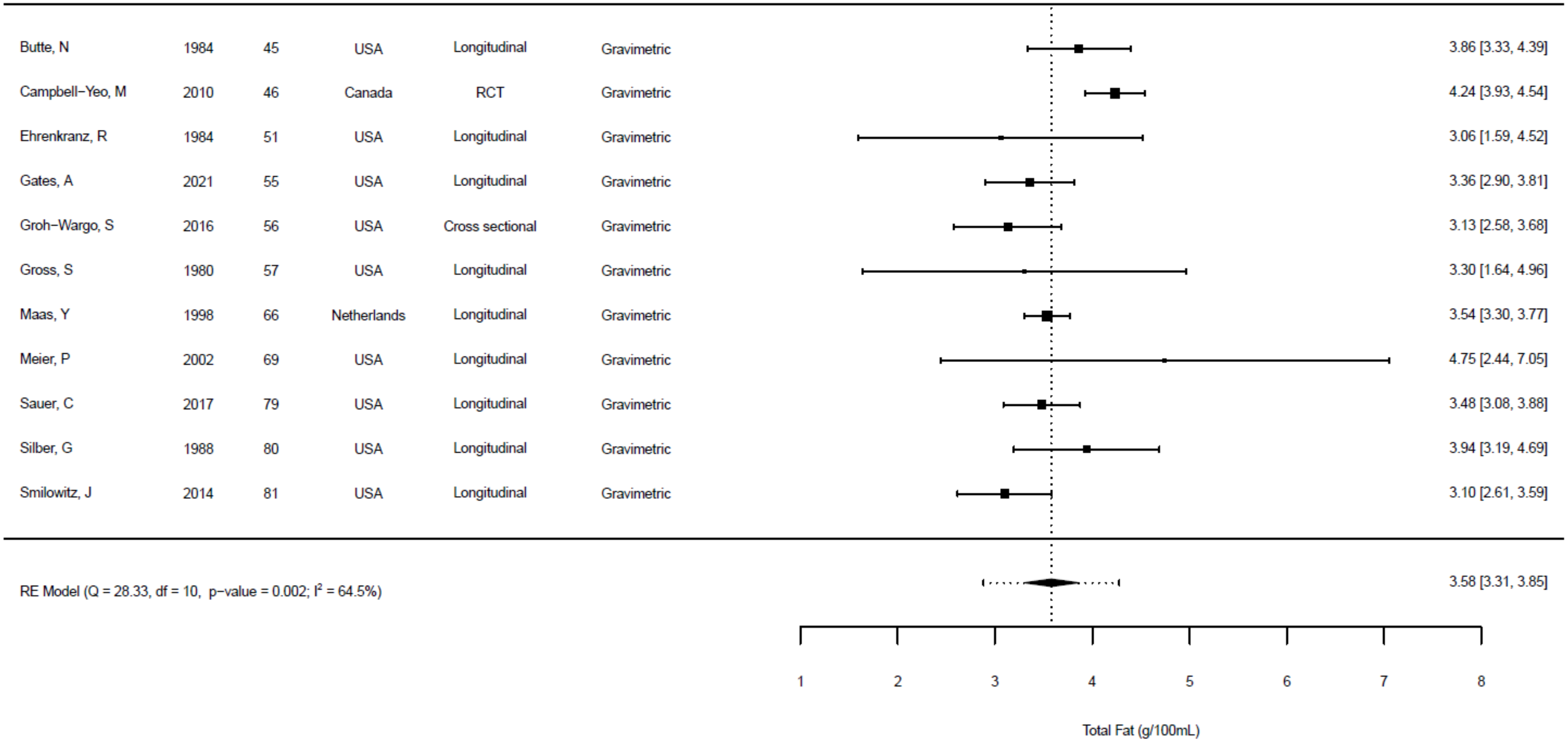

Corresponding Author, Ariana Bailey

Supplementary Figure 8. Fat Analysis by Method, Infrared

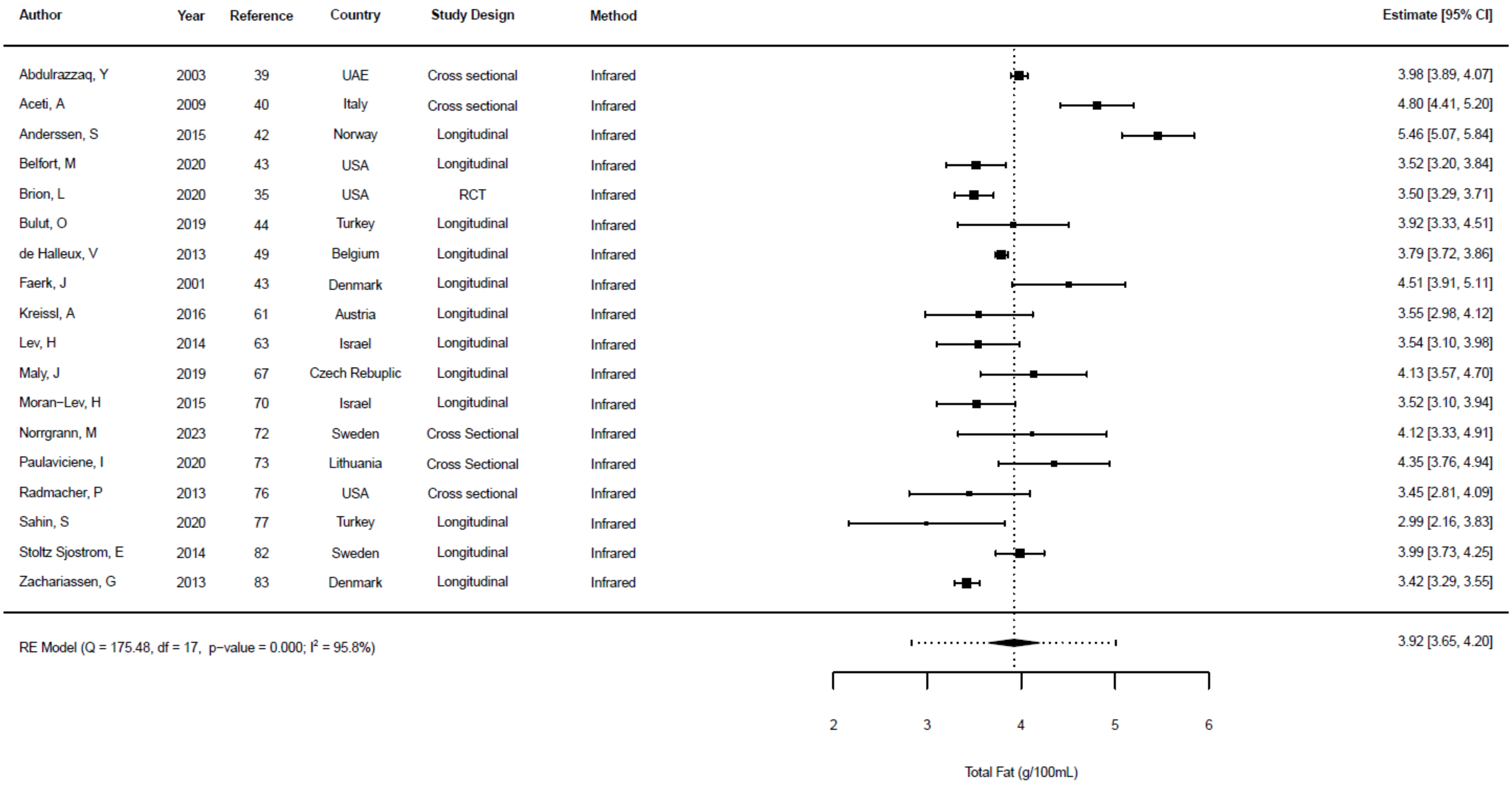

Supplementary Figure 9. Fat Analysis by Method, Other

Author	Year	Reference	Country	Study Design	Method	Estimate [95% CI]
Anderson, D	1983	41	USA	Longitudinal	Other	2.83 [0.95, 4.71]
Corvaglia, L	2008	47	Italy	Cross sectional	Other	3.63 [3.35, 3.91]
Darwish, A	1989	48	Egypt	Longitudinal	Other	3.01 [2.55, 3.47]
de Oliveira, S	2017	50	France	RCT	Other	4.03 [3.71, 4.35]
Gao, C	2020	54	Australia	Longitudinal	Other	3.11 [2.19, 4.03]
Guerrini, P	1981	58	Italy	Longitudinal	Other	2.95 [2.22, 3.67]
Heon, M	2016	59	Canada	RCT	Other	2.98 [2.18, 3.77]
Lemons, J	1982	62	USA	Longtudinal	Other	3.32 [3.07, 3.56]
McLeod, G	2013	68	Australia	Longitudinal	Other	4.64 [4.57, 4.71]
Morton, J	2012	71	USA	Longitudinal	Other	6.18 [5.49, 6.88]
Philip, R	2023	75	Ireland	Cross sectional	Other	3.43 [2.35, 4.51]
Sann, L	1981	78	France	Longitudinal	Other	2.59 [2.21, 2.96]
Thomas, M	1986	17	USA	Longitudinal	Other	3.92 [0.76, 7.08]
Whyte, R	1983	34	Canada	Longitudinal	Other	3.42 [3.11, 3.73]
RE Model (Q = 389.50, df = 13, p−value = 0.000; I^2 = 96.6%)						3.59 [3.07, 4.10]

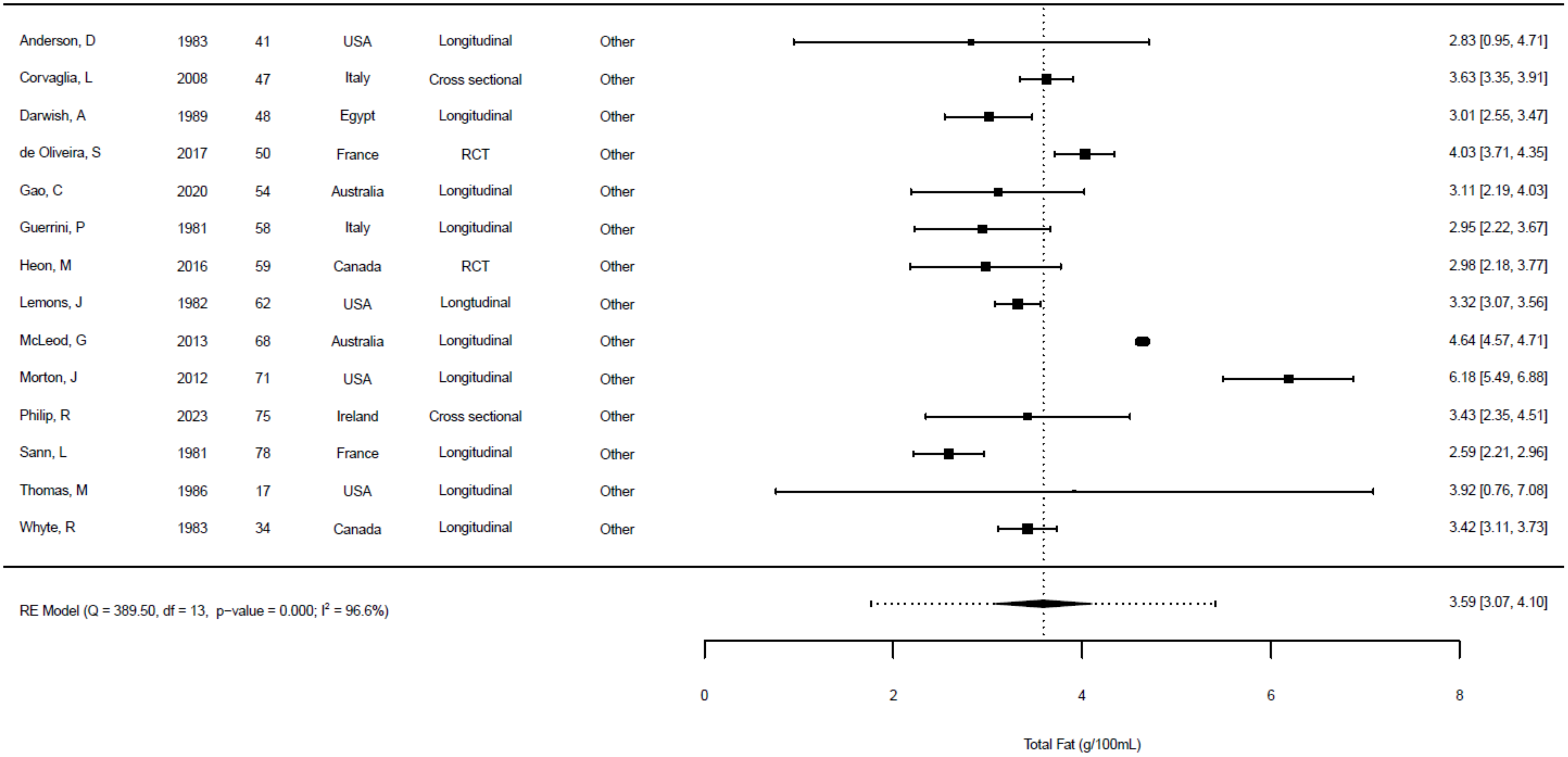

Supplement: Multimedia component 1 [file mmc1.pdf]
